# Supervised learning of a chemistry functional with damped dispersion

**DOI:** 10.1038/s43588-022-00371-5

**Published:** 2022-12-23

**Authors:** Yiwei Liu, Cheng Zhang, Zhonghua Liu, Donald G. Truhlar, Ying Wang, Xiao He

**Affiliations:** 1 Shanghai Engineering Research Center of Molecular Therapeutics and New Drug Development, Shanghai Frontiers Science Center of Molecule Intelligent Syntheses, School of Chemistry and Molecular Engineering, East China Normal University, Shanghai, China; 2 The National and Local Joint Engineering Laboratory of Animal Peptide Drug Development, College of Life Sciences, Hunan Normal University, Changsha, China; 3 Department of Chemistry, Chemical Theory Center, and Minnesota Supercomputing Institute, University of Minnesota, Minneapolis, MN USA; 4 New York University–East China Normal University Center for Computational Chemistry, New York University Shanghai, Shanghai, China

**Keywords:** Density functional theory, Chemical physics, Electronic structure, Electronic properties and materials, Chemical physics

## Abstract

Kohn–Sham density functional theory is widely used in chemistry, but no functional can accurately predict the whole range of chemical properties, although recent progress by some doubly hybrid functionals comes close. Here, we optimized a singly hybrid functional called CF22D with higher across-the-board accuracy for chemistry than most of the existing non-doubly hybrid functionals by using a flexible functional form that combines a global hybrid meta-nonseparable gradient approximation that depends on density and occupied orbitals with a damped dispersion term that depends on geometry. We optimized this energy functional by using a large database and performance-triggered iterative supervised training. We combined several databases to create a very large, combined database whose use demonstrated the good performance of CF22D on barrier heights, isomerization energies, thermochemistry, noncovalent interactions, radical and nonradical chemistry, small and large systems, simple and complex systems and transition-metal chemistry.

## Abstract (web summary)

A hybrid functional (CF22D) with higher across-the-board accuracy for chemistry than most existing non-doubly hybrid functionals is presented by using a large database and a performance-triggered iterative supervised training method.

## Main

The rapid advances of computer capability and the progress of theoretical methods have significantly increased the accuracy of theoretical predictions of chemical, physical, biological, material and atmospheric processes. Relative energies, obtained by electronic structure calculations, are the dominant property controlling molecular and material stability and rate processes, and they play a central role in chemical modelling. Kohn–Sham density functional theory^1,2^ (KS-DFT) has played a major role as the most popular electronic structure framework for modelling the relative energies of large molecules and materials. In principle, KS-DFT is exact, given an exact density functional. However, in practice, density functional approximations (DFAs) are necessary. By adding physical ingredients, enforcing relevant known constraints and optimizing against broader databases^3–7^, DFAs can be made more broadly accurate^8,9^, but existing functionals still leave much room for improvement^10^. Many functionals are accurate only for subsets of chemical properties, and only a few functionals (for example, the doubly hybrid functionals DSD-BLYP-D3(BJ)^11,12^, DSD-PBEP86-D3(BJ)^13^ and B2GPPLYP-D3(BJ)^11,14^) can be applied to make equally accurate predictions on diverse types of chemical systems, such as main-group molecules and transition-metal compounds, large and small systems, bonding and noncovalent (NC) interactions, stable molecules and transition states or radicals and closed-shell systems^3–7^.

An alternative approach to obtain relative energies is molecular mechanics (sometimes called force fields). In this approach, the relative potential energies are represented as functions of molecular coordinates and (optionally) partial atomic charges. This method has been used for more than 70 years, and additional examples are given in Supplementary Section 1.2.

A promising approach, mostly very recent, is to use Big Data and machine learning to improve energy functionals, of either the molecular mechanics or the density functional type. Another powerful development, also old but having advanced in recent years, is the addition of molecular mechanics terms to density functionals to form what one may call a combined quantum mechanical–molecular mechanical energy functional or, for brevity, an energy functional. This broadens the search for DFAs to a search for such more broadly defined energy functionals that can take advantage of both density functionals and molecular mechanics. The present article uses supervised learning to optimize such an energy functional. Supplementary Section 1.2 gives additional references regarding the use of Big Data and machine learning to improve energy functionals and the addition of molecular mechanics terms to density functionals.

In practice, most modern functionals contain parameters that are adjusted in whole or in part to obtain better agreement with experimental data (or, in limited amounts, high-level theoretical data), and the broad advances in the use of machine learning and Big Data now enable ways to train density functionals with larger and more complex data sets. There are functionals with a variety of different combinations of ingredients, and including different ingredients is one way to improve the accuracy. The work presented here differs from previous efforts in that we start with a functional form (the MN15 functional^15^) for a density functional that has already proved successful when optimized with smaller databases, combine it with a molecular mechanics term to account for long-range dispersion interactions and use supervised learning and a large database organized into multiple data sets to simultaneously learn optimum parameters for both components. The form of the MN15 functional was selected for its outstanding performance in early tests and its flexible functional form of nonseparable exchange–correlation energy.

**Fig. 1:**
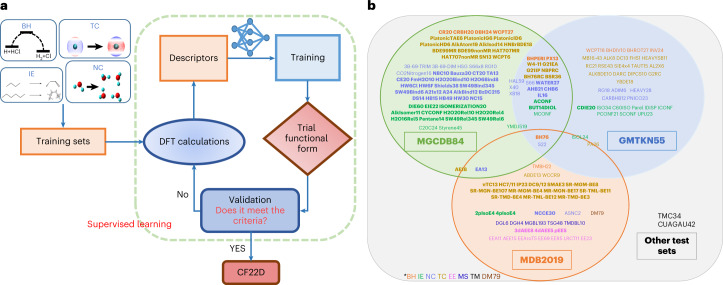
Method and database for the development of CF22D. **a**, The workflow of the development of CF22D. The criterion in the validation step is that, if the MUE of the trial functional for one data set in the validation set is 30% higher than the average MUE of the top five functionals for this data set on the basis of results from ref. ^5^, then this data set is moved from the validation set to the training set based on supervised learning. A new training database is thereby obtained, and the optimization procedure then goes back to the training step. If the MUE of the current training database is converged, and there is no new validation set to be moved into the training set, the procedure ends. **b**, The DDB22 database. Bold text indicates data sets belonging to the training set (see Supplementary Data 1 and Supplementary Table 2 for more details). The orange data sets contain barrier heights (BH), the green data sets contain isomerization energies (IE), the purple data sets contain noncovalent interactions (NC), the golden data sets contain thermochemical properties (TC), the pink data sets contain excitation energies (EE), the dark-blue data sets contain molecular structural data (MS), the brown data set contains dipole moments (DM) and the others are coloured black (transition metals, TM).

The input to a machine-learning algorithm is a set of physical descriptors, and the output is the set of parameters determining the energy as a function of the descriptors. In the approach used here, each term in the MN15 functional is regarded as a physical descriptor, and we also use the molecular geometry as a descriptor. Consequently, the input is a set of integrals of various functionals of the electron density for a set of molecules and the geometries of these molecules, and the parameters are coefficients in a multi-term energy functional that minimizes a loss function. The loss function used here has two components: one measuring errors on a large database of molecular properties, which are mainly relative energies, and a second, regularization term that promotes the smoothness of the resulting energy functional. Supervised learning is used as a key part of the optimization process. The final energy functional obtained from this work is called Chemistry Functional 2022 with damped Dispersion (CF22D). Our workflow is summarized schematically in Fig. 1a.

## Results

The functional form of CF22D and a discussion of how we optimized the functional are presented in Methods section with additional details in Supplementary Section 1. The parameters of the CF22D functional are given in Supplementary Table 1. To assess the performance of the CF22D functional, we compare the results of CF22D against those obtained with other representative functionals on several well-known databases, namely GMTKN55 (ref. 4), Minnesota DataBase 2019 (MDB2019)^3^, MGCDB84 (ref. 5) and the transition-metal data sets of CUAGAU42 (ref. 6) and TMC34 (ref. 7). The consolidated database DDB22 proposed in this work is also used for the assessment. All component data sets of DDB22 are shown in Fig. 1b with detailed explanations given in Supplementary Data 1.

The functionals against which we compare are listed with references in Supplementary Table 3, where they are separated into groups on the basis of their ingredients. We especially note the category of doubly hybrid functionals^16,17^, which include correlation contributions based on unoccupied orbitals. This can add accuracy but also increases the cost. The functionals considered for each sub-database are specified in Tables  1 and 2. Since the doubly hybrid functionals are more expensive than the other functionals and the recent deep learning functional DM21 is quite different from the other functionals, we first compare only the 29 other functionals in Supplementary Table 4. For brevity, we call these ordinary functionals.

**Table 1:** The 25 density functionals compared for all sub-databases

PBE^a^	PBE-D3(BJ)^a^	TPSS^a^	TPSS-D3(BJ)^a^	M06-L^a^
M06-L-D3(0)^a^	MN15-L^a^	ωB97X-D^b^	M11^b^	M11-D3(BJ)^b^
B3LYP^c^	B3LYP-D3(BJ)^c^	PBE0^c^	PBE0-D3(BJ)^c^	M05-2X^c^
M05-2X-D3(0)^c^	PW6B95-D3(BJ)^c^	M06-2X^c^	M06-2X-D3(0)^c^	M06^c^
M06-D3(0)^c^	M08-HX^c^	MN15^c^	MN15-D3(BJ)^c^	CF22D^c^

^a^Local functionals

^b^Range-separated hybrid functionals

^c^Global hybrid functionals

### Performance on the GMTKN55 database

The GMTKN55 database, consolidated by Grimme and coworkers^4^, covers thermochemistry (TC), kinetics and NC interactions of main-group elements. Morgante and Peverati^18^ pointed out that GMTKN55 has more accurate reference values than MGCDB84, because the latter was mainly built based on GMTKN30, which is a predecessor version of GMTKN55. Therefore, the GMTKN55 database was selected to benchmark the performance of CF22D for general chemical properties of main-group elements.

The 1,505 data of GMTKN55 can be partitioned into five sub-databases, namely basic properties and reaction energies for small systems (the ‘small’ sub-database, comprising 18 data sets with 473 data), reaction energies for large systems and isomerization reactions (‘large’, comprising 9 data sets with 243 data), reaction BHs (‘BH’, comprising 7 data sets with 194 data), intermolecular NC interactions (‘inter-NC’, comprising 12 data sets with 304 data) and intramolecular NC interactions (‘intra-NC’, comprising 9 data sets with 291 data). Another classification is to divide the 55 data sets into two sub-databases: Radical7 and Nonradical48 (refs. ^4,19^). The former includes the G21EA, G21IP, SIE4x4, ALKBDE10, HEAVYSB11, RC21 and RSE43 data sets, while the latter includes the rest of the data sets in GMTKN55.

Goerigk et al. introduced the weighted total mean absolute deviation (WTMAD) measures WTMAD-1 and WTMAD-2 (refs. ^4,20^) for comparison of the performance of density functionals on GMTKN55. Explanations of WTMAD-1 and WTMAD-2 are given in Supplementary Section 2.2. Supplementary Table 6 and Supplementary Data 2 give the resulting WTMAD results. Supplementary Table 4 provides the mean unsigned error (MUE), and Supplementary Table 7 provides the mean of the mean absolute error (MoM). CF22D gives the lowest MUE and the second lowest WTMAD-1 and WTMAD-2 among the 29 ordinary functionals, whereas ωB97M-V gives the second lowest MUE and the lowest WTMAD-1 and WTMAD-2. We conclude that the CF22D and ωB97M-V functionals perform similarly well on the GMTKN55 benchmark data for main-group chemistry.

Using the WTMAD-1 and WTMAD-2 measures, we find that CF22D is among the five best-performing functionals for each category in the five-category partition (small, large, BH, inter-NC and intra-NC) among the 29 selected ordinary functionals. In some cases, CF22D even shows better results than some doubly hybrid functionals and the DM21 functional. For example, for the overall WTMAD-1 results, following DSD-BLYP-D3(BJ), B2GPPLYP-D3(BJ), DM21 and ωB97M-V, CF22D does better (2.15 kcal mol^−1^) than B2PLYP-D3(BJ) and MPW2PLYP-D3(BJ) (2.30 and 2.36 kcal mol^−1^, respectively). CF22D outperforms all five doubly hybrid functionals for the BH category with a WTMAD-1 of 1.43 kcal mol^−1^ and is only slightly inferior to DM21 with a WTMAD-1 of 1.35 kcal mol^−1^.

**Fig. 2:**
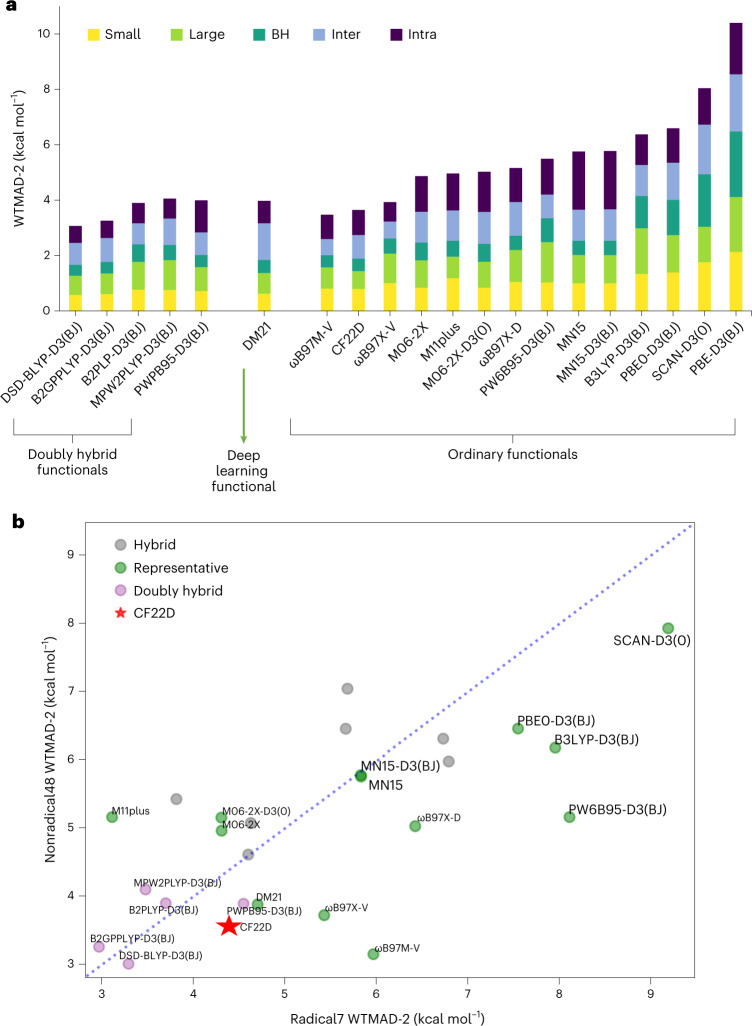
Performance on the GMTKN55 database. **a**, WTMAD-2 of selected functionals for the GMTKN55 database and its sub-databases. **b**, WTMAD-2 for the Radical7 sub-database versus WTMAD-2 for the Nonradical48 sub-database as calculated by CF22D and selected other functionals. The definitions of the sub-databases are similar to those in Supplementary Table 4 but for WTMAD-2. Source data

For the overall WTMAD-2 analysis, DSD-BLYP-D3(BJ), B2GPPLYP-D3(BJ), ωB97M-V and CF22D give the four best results among the 35 compared functionals (the 29 selected ordinary functionals, DM21 and five doubly hybrid functionals) with WTMAD-2 of 3.07, 3.26, 3.47 and 3.64 kcal mol^−1^, respectively. These four functionals outperform B2PLYP-D3(BJ), ωB97X-V, DM21, PWPB95-D3(BJ) and MPW2PLYP-D3(BJ) (with WTMAD-2 of 3.90, 3.93, 3.97, 3.99 and 4.06 kcal mol^−1^, respectively). In the WTMAD-2 analysis (Fig. 2a), the results for the DM21 functional in the Large, BH and Inter categories are not as good as those of CF22D.

For the Large category, CF22D is the best-performing functional and in particular outperforms all five doubly hybrid functionals and the DM21 functional. In this category, it is especially interesting to discuss the MB16-43 data set in GMTKN55 (ref. ^4^). MB16-43 was proposed in the spirit of ‘mindless benchmarking’^21^ and contains the energies of decomposition of 43 artificial molecules. Among the 29 selected ordinary functionals, the average MUE of MB16-43 is 26.77 kcal mol^−1^, and 26 out of those 29 functionals have MUEs that exceed 15 kcal mol^−1^ (Supplementary Data 3). The top performing functionals for MB16-43 are (in order of performance) DM21, PWPB95-D3(BJ), DSD-BLYP-D3(BJ), PW6B95-D3(BJ), B2GPPLYP-D3(BJ), CF22D and ωB97M-V, with MUEs in the range of 6.65–14.82 kcal mol^−1^. It is especially notable that CF22D (10.99 kcal mol^−1^) shows better performance than ωB97M-V (14.82 kcal mol^−1^), two of the doubly hybrid functionals (B2PLYP-D3(BJ) with 16.62 kcal mol^−1^ and MPW2PLYP-D3(BJ) with 22.08 kcal mol^−1^) and the Minnesota functionals.

The results when using the doubly hybrid functionals and the deep learning functional for Radical7 and Nonradical48 are compared with ordinary functionals in another way in Fig. 2b. We see that doubly hybrid functionals are mostly located in the lower left corner of the graph. B2GPPLYP-D3(BJ) and DSD-BLYP-D3(BJ) are the best-performing doubly hybrid functionals for the Radical7 and Nonradical48 sub-databases, respectively. We also see that CF22D is the only functional without doubly hybrid character that lies in the lower left corner, again demonstrating its excellent and balanced performance for both radical and nonradical systems. In fact, the performance of CF22D is comparable to some of the doubly hybrid functionals. For instance, CF22D gives lower MUEs for both Radical7 and Nonradical48 as compared with PWPB95-D3(BJ). For Nonradical48, CF22D also performs better than the other two examined doubly hybrid functionals (B2PLYP-D3(BJ) and MPW2PLYP-D3(BJ)). In addition, as compared with the state-of-the-art deep learning functional DM21, CF22D gives better performance for both Radical7 and Nonradical48. We conclude that the performance of CF22D is competitive with DM21 and CF22D shows high accuracy across diverse types of chemical properties.

### Performance on the AME418 sub-database

**Fig. 3:**
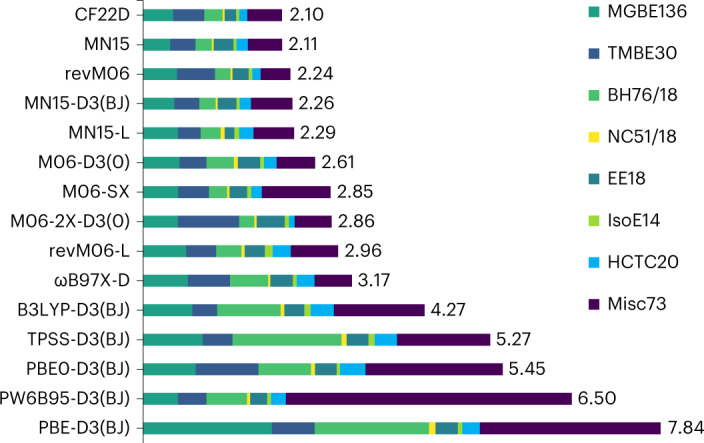
Performance on the AME418 database. The MUEs (kcal mol^−1^) for AME418 and its sub-databases: main-group bond energies (MGBE136), transition-metal bond energies (TMBE30), BHs (BH76/18), NC interactions (NC51/18), excitation energies (EE18), IEs (IsoE14), hydrocarbon TC (HCTC20) and miscellaneous (Misc73). See Supplementary Section 2.3 for the classification of sub-databases. Source data

The AME418 sub-database of MDB2019 is in the training set for optimization of the CF22D functional. As shown in Fig. 3, CF22D gives the lowest MUE (2.10 kcal mol^−1^), followed by MN15, revM06, MN15-D3(BJ) and MN15-L (all with MUE values <2.30 kcal mol^−1^). The results of the comparisons of CF22D against 28 other functionals on AME418 are shown in Supplementary Data 4.

A portion (350 data points) of AME418 was divided into single-reference systems (SR297) and multi-reference systems (MR53)^15,22^. For SR297, CF22D performs the best (MUE of 1.57 kcal mol^−1^). For MR53, the performance of CF22D ranks eighth with an MUE (5.37 kcal mol^−1^) that is much better than the average (7.97 kcal mol^−1^) but not as good as the best (3.96 kcal mol^−1^ for MN15-L).

The full AME418 database is next subdivided into eight sub-databases: main-group bond energies (MGBE136), transition-metal bond energies (TMBE30), BHs (BH76/18), NC interactions (NC51/18), excitation energies (EE18), isomerization energies (IEs) (IsoE14), hydrocarbon TC (HCTC20) and miscellaneous (Misc73). As shown in Supplementary Data 4, CF22D gives the second best results for the MGBE136, EE18 and IsoE14 sub-databases, the third best results for the NC51/18 and HCTC20 sub-databases and the fourth best result for the Misc73 sub-database. On the remaining two sub-databases (TMBE30 and BH76/18) it does not rank in the top nine, but its MUEs of 6.61 and 1.53 kcal mol^−1^ are still considerably better than the average for these sub-databases of 8.79 and 3.31 kcal mol^−1^, respectively.

### Performance on the MGCD84 database

The MGCDB84 database^23^ has 4,986 data. The data for NC interactions and thermochemical properties account for 41.7% and 24.2% of the database, respectively. Portions of MGCDB84 were used in training ωB97M-V and CF22D. For MGCDB84, CF22D has an MUE of 0.80 kcal mol^−1^, behind only ωB97M-V (with an MUE of 0.71 kcal mol^−1^). The MN15 and MN15-D3(BJ) functionals are the seventh and eighth best in our comparison, with MUEs of 1.18 and 1.20 kcal mol^−1^, respectively. The improvement of CF22D with respect to MN15 and MN15-D3(BJ), which share the same functional form for the density functional form, is a measure of the improvement made by the present supervised learning optimization.

The MGCDB84 database is divided^5^ into eight sub-databases: NC ‘easy’ dimers (NCED, 1,744 data), NC ‘easy’ clusters (NCEC, 243 data), NC ‘difficult’ interactions (NCD, 91 data), ‘easy’ IEs (EIE, 755 data), ‘difficult’ IEs (DIE, 155 data), TC ‘easy’ (TCE, 947 data), TC ‘difficult’ (TCD, 258 data) and BHs (BH, 206 data). The RG10 (569 data) and AE18 (18 data) data sets do not fall into any of these sub-databases. Supplementary Table 8 presents the MGCDB84 results for 27 functionals (see Table 2 for details about the compared functionals) listed in order of their overall MUEs. CF22D gives the best performance on the NCD, TCE and TCD sub-databases and the second lowest MUE for the DIE and BH sub-databases. CF22D is among the five best-performing functionals for all eight sub-databases.

**Table 2:** Additional functionals compared for selected sub-databases

DDB22 (25 functionals)	GMTKN55 (35 functionals)		MDB2019 (29 functionals)	MGCDB84 (27 functionals)	CUAGAU42 + TMC34 (30 functionals)
None	SCAN-D3(0)^a^	DSD-BLYP-D3(BJ)^d^	revM06-L^a^	ωB97X-V^b^	revM06-L^a^
	M11plus^b^	B2GPPLYP-D3(BJ)^d^	revM11^b^	ωB97M-V^b^	ωB97X-V^b^
	ωB97X-V^b^	B2PLYP-D3(BJ)^d^	M06-SX^b^		ωB97M-V^b^
	ωB97M-V^b^	MPW2PLYP-D3(BJ)^d^	revM06^c^		M06-SX^b^
	DM21^b^	PWPB95-D3(BJ)^d^			revM06^c^

^a^Local functionals

^b^Range-separated hybrid functionals

^c^Global hybrid functionals

^d^Doubly hybrid functionals

### Performance on the GSE6075 database

Next consider the 6,075 ground-state energies (GSE6075) in DDB22. Supplementary Table 9 shows that CF22D outperforms the 24 compared functionals (see Table 1 for details about the compared functionals) with an MUE of 1.03 kcal mol^−1^. MN15, MN15-D3(BJ), ωB97X-D and M06-2X-D3(0) are the next in the ranking (with MUEs of 1.45–1.52 kcal mol^−1^). Comparing CF22D with MN15-D3(BJ) reveals the huge improvement due to the supervised learning optimization.

Of the 6,075 ground-state energies, 2,866 were used for training and 3,209 were used only for testing. Supplementary Table 9 shows that CF22D is the best-performing functional for both the training and the non-training (testing) sub-databases, with MUEs of 1.34 and 0.75 kcal mol^−1^, respectively. The smaller MUE for the non-training data as compared with the training data apparently arises because the non-training data are easier to predict. The MUE averaged over 25 functionals is 45% smaller for the non-training data, and the corresponding percentage for CF22D is 44%. The commensurate performance across the two subsets provides evidence that the training does not suffer from overfitting and indicates good transferability of the prediction accuracy for the ground-state chemical properties.

The data in GSE6075 can be classified into four types of four chemical properties: BH, NC, IE and TC. Supplementary Table 10 shows that, among the 25 functionals compared (see Table 1 for details about the compared functionals), CF22D demonstrates the best performance for all four classes. For the IE1119 sub-database, CF22D, ωB97X-D and PW6B95-D3(BJ) give the top three performances with MUEs of 0.54, 0.79 and 0.80 kcal mol^−1^, respectively. For the TC1833 sub-database, the three best-performing functionals are CF22D, MN15 and MN15-D3(BJ) with MUEs of 2.44, 3.50 and 3.57 kcal mol^−1^, respectively. For the BH318 sub-database, the top five performing functionals are CF22D, M08-HX, MN15, MN15-D3(BJ) and ωB97X-D, with CF22D having an MUE of 1.31 kcal mol^−1^ and the other four having MUEs in the range of 1.50–1.69 kcal mol^−1^. For the NC2805 category, the two top-performing functionals are CF22D and ωB97X-D with MUEs of 0.27 and 0.29 kcal mol^−1^, respectively.

We can divide each of the four classes into training and testing data, and Supplementary Table 10 shows that CF22D has the best performance for six of them (BH_test112, NC_training936, IE_training293, IE_test826, TC_training1431 and TC_test402 categories), the second best for the BH_training206 category and the fifth best for the NC_test1869 category. The comparisons presented in Supplementary Tables 9 and 10 show that CF22D gives excellent performance for various types of properties and demonstrate that the predictive accuracy of the CF22D functional is highly transferable to properties that are not in the training set.

**Fig. 4:**
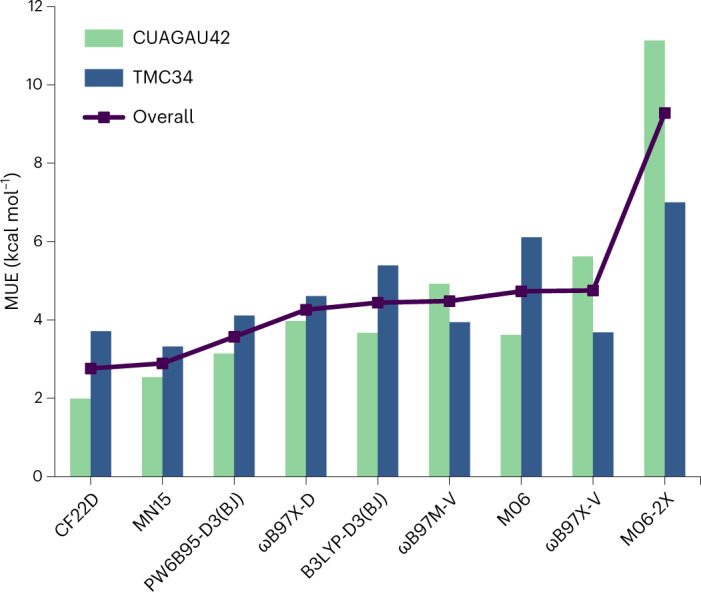
MUEs for CUAGAG42 and TMC34. Results for the ωB97M-V and ωB97X-V functionals are taken from refs. ^6,7^, while all the other results are calculated in this study. The label ‘overall’ denotes the overall MUE on the 76 data in the CUAGAU42 and TMC34 data sets. Source data

For the ground-state energies in DDB22, CF22D is not only the best-performing functional for the full set of 6,075 data among the 25 selected representative functionals but also the best functional for each of the four sub-databases NC, TC, BH and IE. We found that CF22D also shows excellent transferability on the diverse non-training test sets of transition-metal chemistry, including CUAGAU42, TMC34, TMBH22 and WCCR9. The MUE of CF22D for the whole set of 107 testing transition-metal data (that were not used for training) is 2.77 kcal mol^−1^ (Supplementary Table 11), which is the best among all the tested functionals. Especially for the CUAGAU42 and TMC34 data sets, we can compare the performance with ωB97M-V (Fig. 4). CF22D gives the best performance on the CUAGAU42 data set and also the best results overall. Detailed results for CF22D’s performance on the CUAGAU42 and TMC34 databases can be found in Supplementary Section 2.5.1.

CF22D is demonstrated to be an excellent energy functional for ‘complex’ systems with an MUE of 2.84 kcal mol^−1^ for the 886 data classified as complex (Supplementary Table 14).

### Dispersion interactions

**Fig. 5:**
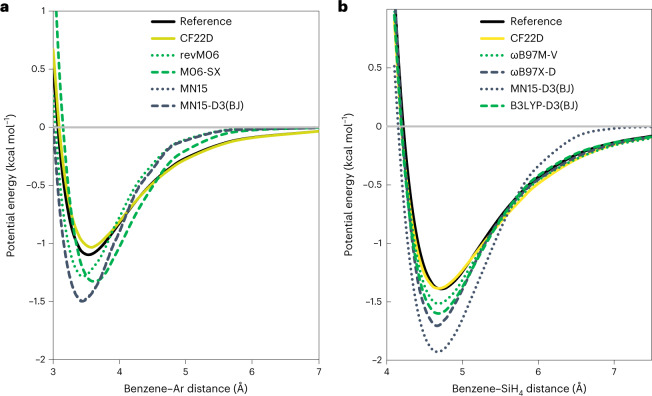
Benzene–Ar and Benzene–SiH_4_ potential energy curves. **a**,**b**, Potential energy curves for benzene–Ar (**a**) and benzene–SiH_4_ (**b**) calculated using the CF22D and other functionals with the (99, 590) integration grid and the def2-QZVPPD basis set, as compared with reference results (black curves) from coupled cluster theory with single and double excitations and a quasiperturbative treatment of connected triple excitations (CCSD(T)) calculations. The geometries and reference energies of benzene–Ar and benzene–SiH_4_ are obtained from ref. ^27^. Source data

Figure 5a shows the potential energy curves of benzene–Ar calculated by three DFAs (M06-SX, revM06 and MN15) and two energy functionals with molecular mechanics (MN15-D3(BJ) and CF22D). The former DFAs, because they do not have nonlocal correlation and hence do not have long-range dispersion^24^, give curves that decay to zero quickly from 4.5 to 6.0 Å. The dispersion-corrected functional MN15-D3(BJ) shows a negligible long-range tail because the damped dispersion term for MN15 was added without re-optimizing the functional form. Since MN15 gives reasonably good results in the van der Waals region (because it contains a medium-range correlation energy^25,26^), only a small, damped dispersion term was added. CF22D shows good agreement with the reference values both at the equilibrium position and in the long-range region in Fig. 5a.

In Fig. 5b, CF22D shows similar good results for benzene–SiH_4_. This figure also shows that B3LYP-D3(BJ) provides a reliable long-range van der Waals tail but that, at the equilibrium position, it overestimates the benzene–SiH_4_ binding energy by about 0.21 kcal mol^−1^. The geometries and reference energies of benzene–Ar and benzene–SiH_4_ are obtained from ref. ^27^. Overall, CF22D provides generally reliable predictions for NC interactions, not only for the binding energies near the equilibrium distance but also for the weak interactions at long distance.

### Other results

Results for electronic excitation energies, dipole moments, molecular structures, basis set superposition errors and grid errors, binding energies of extra-large complexes (ExL7)^25^, reactions of open-shell single-reference transition metal complexes (ROST61)^28^ and the CUAGAU-2 (ref. ^29^) data set are presented in Supplementary Tables 16–22. CF22D outperforms the selected non-doubly hybrid functionals, especially for ExL7 and the CUAGAU-2 data sets (Supplementary Tables 20 and 22). For the ROST61 data set, the MUE results for the doubly hybrid functionals with a molecular-mechanics damped-dispersion term are listed in Supplementary Table 21, all being lower than 3 kcal mol^−1^. The average value of the results for the functionals with a molecular-mechanics damped-dispersion term is 3.36 kcal mol^−1^, whereas the average value of the results of non-doubly hybrid functionals is 4.64 kcal mol^−1^. CF22D performs well with an MUE of 4.03 kcal mol^−1^, which is better than the average MUE of the non-doubly hybrid functionals.

## Discussion

Density functional theory (DFT) is the most popular electronic structure method, but many functionals are optimized only against limited specific groups of chemical properties, and few functionals can be applied to accurately predict all the properties required for complex chemical applications. We used physical descriptors, broad databases and supervised learning for the systematic optimization of a flexible functional form including the simultaneous optimization of a molecular-mechanics damped-dispersion term. As shown in Results section, CF22D can be recommended for applications involving a broad range of bonding and NC interactions of both main-group and transition-metal compounds, which makes it appropriate for studies of catalysis, functional materials, biochemistry and environmental chemistry. However, as a global hybrid functional, CF22D has limitations because it contains Hartree–Fock (HF) exchange, even at long range: (1) it is not economical for plane wave codes because the treatment of long-range HF exchange in plane wave codes requires a fine mesh for integration over the Brillouin zone^30^, (2) long-range HF exchange causes a divergence of the group velocity at the Fermi level for solid-state systems (such as metals) that do not have a gap^31,32^ and (3) HF exchange is known to cause a static correlation error^33^, although this is ameliorated in the present functional by parameterization to a training set with a high representation of strongly correlated systems. Another limitation is that the long-range dispersion terms do not take account of the partial atomic charge distributions in the interacting subsystems.

Equation (1) is an energy functional based on seven features: spin-up and spin-down electron density, spin-up and spin-down electron density gradient, spin-up and spin-down kinetic energy density and the set of internuclear distances (which are the geometries of the dimers embedded in the molecule). In the future, one may envision more general energy functionals in which the energy also depends on other variables such as the geometries of the trimers embedded in the molecule or other features (for example, ionization potentials) of the atoms, dimers and/or trimers embedded in the molecule. Thus, the energy functional considered here may be considered to be just an example of a move toward more complex energy functionals with a greater variety of features. It has been stated that “Feature selection methods provides us a way of reducing computation time, improving prediction performance, and a better understanding of the data in machine learning”^34^. Therefore, we see a future for density functional theory that may involve combining traditional functionals with functionals of other variables to produce machine learning functionals with even better combinations of accuracy and efficiency.

## Methods

Basing the loss function and the additional testing of the output functional on broad and diverse databases is a key aspect in the present work. We train the functional with a database including nearly 3,000 data. The training data are organized into a variety of energetic data sets for different categories of energies, and we also consider sub-databases encompassing subsets of the data sets. An additional set of about 3,800 data not used for training are used as a testing set. The testing set includes BHs, NC interactions, TC, IEs, excitation energies, bond lengths and dipole moments.

## The density functional term

Our energy functional has two kinds of terms: a DFA and a molecular-mechanics term representing damped dispersion. The functional form is

1 $$E^{{{{\mathrm{CF}}}}22{{{\mathrm{D}}}}} = E_{{{{\mathrm{DF}}}}} + E_{{{{\mathrm{disp}}}}},$$

where the *E*_DF_ is an exchange–correlation term with the functional form of the successful MN15 functional and *E*_disp_ is a molecular-mechanics term that is conventionally called a damped dispersion term. Note that the damped dispersion term accounts for more than dispersion at short range, and dispersion is not uniquely defined for geometries where there is overlap of the wave functions of interacting subsystems.

The parameters in *E*_DF_ were optimized simultaneously with a parameter in *E*_disp_. For *E*_DF_, we chose the form of the previously successful MN15 functional^15^. This is a linear combination of the nonlocal single-determinant exchange energy $E_{{{\mathrm{x}}}}^{{{{\mathrm{HF}}}}}$, a local nonseparable exchange–correlation energy *E*_nxc_ and an additional correlation energy *E*_c_:

2 $$E_{\mathrm{DF}} = \frac{X}{{100}}E_{{{\mathrm{x}}}}^{{{{\mathrm{HF}}}}} + E_{{{{\mathrm{nxc}}}}} + E_{{{\mathrm{c}}}},$$

3 $$\begin{array}{l}E_{{{{\mathrm{nxc}}}}} =\\ {\int} {{\mathrm{d}}{{{\mathbf{r}}}}\mathop {\sum}\limits_{\sigma = \alpha }^\beta {\rho _\sigma \left\{ {\varepsilon _{{{{\mathrm{x}}}}\sigma }^{{{{\mathrm{LSDA}}}}}\left( {\rho _\sigma } \right)\mathop {\sum}\limits_{i = 0}^3 {\mathop {\sum}\limits_{j = 0}^{3 - i} {\mathop {\sum}\limits_{k = 0}^{5 - i - j} {a_{ijk}\{ v_{{{{\mathrm{x}}}}\sigma }(\rho _\sigma )\} ^i\{ u_{{{{\mathrm{x}}}}\sigma }(s_\sigma )\} ^j\{ w_\sigma (\rho _\sigma ,\tau _\sigma )\} ^k} } } } \right\}} },\end{array}$$

4 $$\begin{array}{l}E_{{{\mathrm{c}}}} = {\int} {{\mathrm{d}}{{{\mathbf{r}}}}\,\rho \,\varepsilon _{\mathrm{C}}^{{{{\mathrm{LSDA}}}}}\left( {\rho _\alpha ,\rho _\beta } \right)\left( {\mathop {\sum }\limits_{i = 0}^8 b_i\left\{ {w\left( {\rho ,\tau } \right)} \right\}^i} \right)}\\ \qquad + {{\int} {{\mathrm{d}}{{{\mathbf{r}}}}\,\rho H^{{{{\mathrm{PBE}}}}}(\rho _\alpha ,\rho _\beta ,s)\left( {\mathop {\sum}\limits_{i = 0}^8 {c_i\left\{ {w(\rho ,\tau )} \right\}^i} } \right)} },\end{array}$$

where

5 $$s_\sigma = \frac{{\left| {\nabla \rho _\sigma } \right|}}{{\rho _\sigma ^{4/3}}}.$$

*X* is the percentage of HF exchange $E_{{{\mathrm{x}}}}^{{{{\mathrm{HF}}}}}$, *ρ*_*α*_ and *ρ*_*β*_ are the up-spin and down-spin electron densities at the spatial point ***r***, *ρ* is their sum, *τ*_*α*_ and *τ*_*β*_ are the spin-up and spin-down kinetic energy density and the functions *v*_x*σ*_, *u*_x*σ*_, *w*_*σ*_, $\varepsilon _{{{{\mathrm{x}}}}\sigma }^{{{{\mathrm{LSDA}}}}}$, $\varepsilon _{\mathrm{C}}^{{{{\mathrm{LSDA}}}}}$ and *H*^PBE^ are the same as used in the MN15 functional^15^ and are therefore not re-explained here. The parameters *X, a*_*ijk*_*, b*_*i*_ and *c*_*i*_ in equations (2–4) of CF22D are shown in Supplementary Table 1.

### Damped dispersion

The DFT-D3(0) model^35^ is the starting point for the molecular-mechanics term used here. The D3(0) treatment has *r*_*AB*_^–6^ and *r*_*AB*_^–8^ terms, where *r*_*AB*_ is the distance between atoms A and *B*, but only the *r*_*AB*_^−^^6^ term is used in the present work because our goal is to obtain only the longest-range dispersion term by molecular mechanics. The term we use has the unscaled form

6 $$E_{{{{\mathrm{disp}}}}} = - \frac{1}{2}\mathop {\sum}\nolimits_{AB} {\frac{{C_6^{AB}}}{{r_{AB}^6}}} f_{d,6}\left( {r_{AB}} \right),$$

where the sum includes all the atom pairs in the system, $C_6^{AB}$ is the D3(0) dispersion coefficient that depends on the atomic coordination numbers CNA and CN^*B*^, which depend on the system’s geometry, and

7 $$f_{d,6}\left( {r_{AB}} \right) = \frac{1}{{1 + 6\left( {r_{AB}/(s_{r,6}R_0^{AB})} \right)^{ - 14}}},$$

where *s*_*r,*6_ is a scaling parameter optimized in the present work and $R_0^{AB}$ is the pair-specific cut-off radius parameterized in DFT-D3(0) for the 4,465 values of all atom pairs *AB* composed of the first 94 elements of the Periodic Table^35^. The optimization method of *s*_*r,*6_ for CF22D is presented in Supplementary Section 1, and the resulting value of *s*_*r,*6_ is provided in Supplementary Table 1.

### The loss function

The loss function is

8 $$L = \mathop {\sum}\limits_{n = 1}^K {R_n/I_n + \lambda (a + b + c)},$$

in which

9 $$a = \mathop {\sum}\limits_{i = 0}^3 {\mathop {\sum}\limits_{j = 0}^{3 - i} {\mathop {\sum}\limits_{k = 0}^{4 - i - j} {(a_{i,j,k} - a_{i,j,k + 1})^2} } },$$

10 $$b = \mathop {\sum}\limits_{i = 0}^7 {(b_i - b_{i + 1})^2},$$

11 $$c = \mathop {\sum}\limits_{i = 0}^7 {(c_i - c_{i + 1})^2},$$

and *K* is the number of training data sets, *R*_*n*_ is the r.m.s. error (RMSE) for data set *n* in Supplementary Data 5, *I*_*n*_ is the inverse weight of subset *n*, *λ*(*a* *+* *b* *+* *c*) is an *L*_2_ regularization term that serves as a smoothness restraint^36,37^ and *λ* is a smoothing coefficient^37^ that was set to 0.01 for CF22D.

The value of the loss function depends on the inverse weights. Our goal in training the energy functional was to obtain small errors across the board, that is, relatively small errors for as many data sets and sub-databases as possible, not to simply reduce the overall mean unsigned error for the entire training data set or the absolute value of the loss function. The final selection of the inverse weights was therefore determined iteratively by substantial trial and error to obtain uniformly good performance across the full collection of data sets, as discussed below.

### The DDB22 database

In this work, we built a combined database called the Diverse Database 2022 (DDB22), which includes 155 data sets made up of a total of 6,572 data. All the component data sets are shown in Fig. 1b, with detailed explanations given in Supplementary Data 1. The data sets of the DDB22 database come from five sources:

- The Minnesota DataBase 2019 (MDB2019), a composite and update by Verma et al.^3,10,38^ of an earlier Minnesota database. It contains energetic data, geometric data and dipole moments. The energetic data include bond energies, reaction energies, proton affinities, electron affinities, ionization potentials, NC interaction energies and reaction BHs for main-group compounds and transition-metal compounds plus total atomic energies and electronic excitation energies. The geometric data consist of bond lengths, which are equilibrium interatomic distances between bonded atoms. The present study omitted the lattice constants in MDB2019 because we only consider gas-phase data in the present development. A subset, called AME418, of MDB2019 is a set of 418 atomic and molecular energies used as components of the training sets for the revM11 (ref. ^38^) and M06-SX^39^ functionals.
- The Main-Group Chemistry Database MGCDB84 database, compiled by Mardirossian and Head-Gordon^5^ “from the benchmarking activities of numerous groups, including Hobza, Sherrill, Truhlar, Herbert, Grimme, Karton, and Martin”. It comprises 84 data sets containing 4,986 data for NC interactions, IEs, TC and BHs. NC interactions are especially well represented.
- The GMTKN55 database of Goerigk et al.^4^ for general main-group TC, kinetics and NC interactions.
- The transition-metal chemistry database TMC34, developed by Chan et al.^7^ as representative of a much larger database of metal–organic reaction energies, dissociation energies of diatomic transition-metal species and reaction barriers involving complexes of second- and third-row transition metals. It is divided into the TC data sets TMD10 and MOR13 and the BH data set TMB11.
- The CUAGAU42 database of Chan^6^ for small copper, silver and gold compounds. It contains two data sets: CUAGAU_TC27 for TC and CUAGAU_IE15 for IEs.

Data sets from various databases have some degree of overlap. The MGCDB84 database includes the GMTKN30 (ref. ^40^) database (a predecessor of GMTKN55 that is partially represented and partially updated in GMTKN55) and previous Minnesota databases, and the GMTKN55 database also has some overlap with previous Minnesota databases. The overlapping data of MDB2019, GMTKN55 and MGCDB84 are shown in Fig. 1b (see Supplementary Table 2 for more details on these overlaps and how they were resolved to create the consolidated database).

We used the entire DDB22 to compare the performance of the CF22D functional with selected other functionals, but only a portion of it was used for the training and validation steps. For some of the discussion, to better understand the validation and testing tests, we divide DDB22 into four sub-databases:

- Ground-state energies (sub-database GSE6075, with energies in kcal mol^−1^) that consists of 6,075 data of ground-state energetic data from 13 data sets of BHs, 44 NC interaction energy data sets, 30 IEs data sets and 55 TC data sets (this sub-database contains 6,057 relative energies and 18 absolute atomic energies)
- Excitation energies (EE157, with electronic excitation energies in eV), consisting of 157 data of excitation energetic data from ten data sets
- Molecular structures (MS261, with interatomic distances in Å) consisting of 261 data from five molecular structure data sets
- Dipole moments (DM79, with dipole moments in Debye) consisting of 79 data from one database of dipole moments

These classifications are specified in detail in Supplementary Data 1.

### Training

Our learning scheme involves performance-triggered iterative supervised training. For brevity, we call this supervised learning. Our supervised learning scheme differs from the active learning schemes that were developed for labelling problems. In those cases, the machine queries the supervisor about troublesome unlabelled data, and the supervisor labels the data^41^. Our application is in the regression and prediction area rather than the labelling area. Our supervised learning scheme is closer to the active learning method developed by Zhang et al.^42^ for neural net modelling of force fields, but with some differences because we group data into data sets of related data and because we do not use a neural net. Our method also differs from machine learning schemes that divide the data randomly among the training and validation sets in that we divide the data in a more organized fashion using the data sets. The three steps in our supervised learning, following the development of the initial model with an initial training set, are as follows: (1) wider testing in a step that replaces the conventional validation step with one that uses the current model to explore additional data sets spanning a broader domain than had been used to develop the existing model and identifies poorly fit data sets; (2) augmentation, in which we add the troublesome data sets to the training set; (3) retraining. The machine develops a model based on the augmented data. We then repeat these steps until convergence is reached. An active learning scheme with this kind of sequence was presented by Schmidt et al.^43^. They described their active learning schemes as follows: “(i) A surrogate model has to be developed; (ii) Based on the prediction of the surrogate model, optimal infill points have to be chosen in order to retrain the surrogate model and finally find the optimum.”.

Our workflow to implement the above supervised learning method is summarized schematically in Fig. 1a. Here we provide a detailed description:

1. We select 79 data sets (data sets 1–79 from AME418 and MGCDB84, listed in Supplementary Data 5) with a total of 1,886 data as the initial training set. The initial inverse weight of each data set in AME418 is the same as the one utilized in the final optimization of the M06-SX functional^39^. The initial inverse weight of each selected data set in MGCDB84 is chosen as the average MUE for that data set as averaged over 200 exchange–correlation functionals (previously published and developed by many different groups) as given in the original MGCDB84 article^5^. Note that Supplementary Data 5 shows 92 data sets with 3,694 data. Data sets 80–92 with 1,808 data constitute the initial validation set. We also initialize the *s*_*r,*6_ parameter in the damped dispersion. Using the standard notation, data sets 1–79 are training data and data sets 80–92 are initially validation data, but some of them are converted to training data by the supervised learning procedure of step 6. The testing data are described in Supplementary Data 1, including test sets in the DDB22 database and three additional testing data sets (ExL7 (ref. 25), ROST61 (ref. 28) and CUAGAU-2 (ref. 29).
2. The electron densities of all systems in the training set are calculated by using the MN15 functional and applied as the initial densities.
3. Each descriptor in the CF22D functional described by equation (1) is calculated for all the systems in the training set based on the electron densities generated by the functional of the previous step (step 2 in the first iteration and step 6 in subsequent ones). The *R*_*n*_ value of each data set in equation (8) can be expressed as a function of *s*_*r,*6_ in equation (7) and the coefficients in the density functional term, namely *X* in equation (2), *a*_*ijk*_ in equation (3) and *b*_*i*_ and *c*_*i*_ in equation (4). Thus, the loss function of equation (8) is a function of those variables and the *I*_*n*_ of each subset.
4. The loss function of equation (8) is minimized using the generalized reduced gradient nonlinear algorithm for a given *s*_*r,*6_ value, and the *s*_*r,6*_ value in equation (7) is varied to minimize the MUE of the training set. (Initially we vary the value of *s*_*r,*6_ from 1.2 to 2.1 Å with an initial interval of 0.1 Å, but the interval is gradually reduced.) This yields a new value of *s*_*r,*6_, and a new set of density functional coefficients is obtained.
5. Using the trial functional obtained from step 4, the energies of all systems in the training and validation sets are calculated, and the MUE for each data set in the training and validation sets is calculated.
6. This is the supervised learning step. If the MUE of the trial functional for one data set in the validation set is 30% higher than the average MUE of the top five functionals for this data set based on the results from ref. ^5^, then this data set is moved to the training set with the inverse weight determined by the same method as used in step 1. We then modify selected inverse weights (both the ones inherited from previous steps and the new ones) to improve, if possible, the performance on the various sub-databases where we wish to reduce the error to obtain small errors across the board. The final selection of inverse weights in this step is determined by substantial trial and error to try to obtain uniformly good performance across the full collection of data sets.
7. If a validation data set is moved into the training set in step 3, the electron densities of all the systems in the training set are recalculated, and we return to step 3. If no new data set is moved in step 6, we compare the MUE of the training set with the value in the previous iteration. If the MUE is not converged, the electron densities of all systems in the training set are recalculated, and we return to step 3. If the MUE of the training database is converged, we proceed to step 8.
8. At convergence, the results of CF22D for all the training and test data sets are calculated and compared with other density functionals.

After five rounds of iteration and validation, the supervised learning added ten data sets containing 1,033 data (data sets 80–89 in Supplementary Data 5). The data sets added by supervised learning are all from the BH76 group (BH76RC and DBH24) and the W4-11 group (HAT707MR, HAT707nonMR, BDE99MR, BDE99nonMR, TAE140MR, TAE140nonMR, ISOMERIZATION20 and SN13) of the MGCDB84 database.

In the final iteration, *s*_*r,*6_ = 1.53 Å, with which the overall MUE of the selected data sets was the lowest (Supplementary Section 1). The optimized parameters of the CF22D functional are given in Supplementary Table 1.

### Computational details

The CF22D calculations were performed using a locally modified version of Gaussian 16 revision A.03 (ref. ^44^), while all the calculations with the other functionals in this work were performed using the unmodified Gaussian 16, revision A.03.

The basis sets, molecular geometries and quadrature grids for the calculations on MDB2019 (refs. ^3,22,39^) were the same as those employed in our previous works^22,39,45^ and can be found in Supplementary Table 21 of ref. ^22^. For the calculations on the GMTKN55 (ref. ^4^) database, MGCDB84 (ref. ^5^) database, transition-metal data sets TMC34 (ref. ^7^) and CUAGAU42 (ref. ^6^), the settings were the same as those employed in the original papers. The basis set is mainly def2-QZVP for GMTKN55 (diffuse functions were applied to some atoms in some of the data sets, and core electrons of heavy elements in some molecules of HEAVYSB11, HAL59 and HEAVY28 were replaced by the def2-ECP effective core potentials). The basis set is def2-QZVPPD for MGCDB84. The basis sets are def2-QZVPP for CUAGAU42, CUAGAU-2 and ROST61, def2-TZVP for TMC34, and cc-pVTZ for ExL7.

A (99, 590) grid (99 radial shells with 590 grid points per shell) was used for all of the data sets, except AE18 and RG10, for which a (500, 974) grid was used.

### Additional data and references

Additional data from this study and additional references are provided in the Supplementary Information.

### Supplementary information

Supplementary Information Supplementary Sects. 1–3, Tables 1–22 and Figs. 1–4.

Supplementary Data 1. Composition of the combined database DDB22.

Supplementary Data 2. WTMAD-2 (kcal mol^−1^) for the GMTKN55 database.

Supplementary Data 3. MUEs (kcal mol^−1^) of selected functionals for the 55 data sets of the GMTKN55 database.

Supplementary Data 4. The MUEs (kcal mol^−1^) for AME418.

Supplementary Data 5. Description of the training sets and validation data sets and the final inverse weights.

Supplementary Data 6. MUEs (kcal mol^−1^) of selected functionals for the 26 energetic data sets of AME418.

Supplementary Data 7. The MUEs (kcal mol^−1^) of the selected functionals for the 84 data sets of MGCDB84 database.

### Source data

Statistical Source Data for Fig. 2.

Statistical Source Data for Fig. 3.

Statistical Source Data for Fig. 4.

Statistical Source Data for Fig. 5.

## Data Availability

The optimized parameters of the CF22D functional are available in Supplementary Table 1. The MUE results for all the data sets discussed in this work can be obtained from Zenodo^46^. Source data for Figs. 2–5 are available with this manuscript.

## Code availability

The Gaussian 16 program (revision A.03) used in this work is commercially available at http://www.gaussian.com/. The Fortran source codes for the CF22D energy functional can be obtained from Zenodo^46^.

## References

[1] Kohn W, Sham LJ (1965). Self-consistent equations including exchange and correlation effects. Phys. Rev..

[2] Hohenberg P, Kohn W (1964). Inhomogeneous electron gas. Phys. Rev..

[3] Verma P, Truhlar DG (2019). Data from “Geometries for Minnesota Database 2019”. Data Repos. Univ. Minn..

[4] Goerigk L (2017). A look at the density functional theory zoo with the advanced GMTKN55 database for general main group thermochemistry, kinetics and noncovalent interactions. Phys. Chem. Chem. Phys..

[5] Mardirossian N, Head-Gordon M (2017). Thirty years of density functional theory in computational chemistry: an overview and extensive assessment of 200 density functionals. Mol. Phys..

[6] Chan B (2019). The CUAGAU set of coupled-cluster reference data for small copper, silver, and gold compounds and assessment of DFT methods. J. Phys. Chem. A.

[7] Chan B, Gill PMW, Kimura M (2019). Assessment of DFT methods for transition metals with the TMC151 compilation of data sets and comparison with accuracies for main-group chemistry. J. Chem. Theory Comput..

[8] Kirkpatrick J (2021). Pushing the frontiers of density functionals by solving the fractional electron problem. Science.

[9] Chen Y, Zhang L, Wang H, E W (2021). DeePKS: A comprehensive data-driven approach toward chemically accurate density functional theory. J. Chem. Theory Comput..

[10] Verma P, Truhlar DG (2020). Status and challenges of density functional theory. Trends Chem..

[11] Goerigk L, Grimme S (2011). A thorough benchmark of density functional methods for general main group thermochemistry, kinetics, and noncovalent interactions. Phys. Chem. Chem. Phys..

[12] Kozuch S, Gruzman D, Martin JML (2010). DSD-BLYP: a general purpose double hybrid density functional including spin component scaling and dispersion correction. J. Phys. Chem. C.

[13] Kozuch S, Martin JML (2011). DSD-PBEP86: in search of the best double-hybrid DFT with spin-component scaled MP2 and dispersion corrections. Phys. Chem. Chem. Phys..

[14] Karton A, Tarnopolsky A, Lamère J-F, Schatz GC, Martin JML (2008). Highly accurate first-principles benchmark data sets for the parametrization and validation of density functional and other approximate methods. derivation of a robust, generally applicable, double-hybrid functional for thermochemistry and thermochemical kinetics. J. Phys. Chem. A.

[15] Yu HS, He X, Li SL, Truhlar DG (2016). MN15: A Kohn-Sham global-hybrid exchange-correlation density functional with broad accuracy for multi-reference and single-reference systems and noncovalent interactions. Chem. Sci..

[16] Zhao Y, Lynch BJ, Truhlar DG (2004). Doubly Hybrid Meta DFT: New multi-coefficient correlation and density functional methods for thermochemistry and thermochemical kinetics. J. Phys. Chem. A.

[17] Schwabe T, Grimme S (2006). Towards chemical accuracy for the thermodynamics of large molecules: new hybrid density functionals including non-local correlation effects. Phys. Chem. Chem. Phys..

[18] Morgante P, Peverati R (2019). ACCDB: A collection of chemistry databases for broad computational purposes. J. Comput. Chem..

[19] Janesko BG, Verma P, Scalmani G, Frisch MJ, Truhlar DG (2020). M11plus, a range-separated hybrid meta functional incorporating nonlocal rung-3.5 correlation, exhibits broad accuracy on diverse databases. J. Phys. Chem. Lett..

[20] Goerigk L, Grimme S (2010). A general database for main group thermochemistry, kinetics, and noncovalent interactions − assessment of common and reparameterized (meta-)GGA density functionals. J. Chem. Theory Comput..

[21] Korth M, Grimme S (2009). “Mindless” DFT benchmarking. J. Chem. Theory Comput..

[22] Wang Y, Verma P, Jin X, Truhlar DG, He X (2018). Revised M06 density functional for main-group and transition-metal chemistry. Proc. Natl Acad. Sci. USA.

[23] Mardirossian N, Head-Gordon M (2016). ωB97M-V: a combinatorially optimized, range-separated hybrid, meta-GGA density functional with VV10 nonlocal correlation. J. Chem. Phys..

[24] Truhlar DG (2019). Dispersion forces: Neither fluctuating nor dispersing. J. Chem. Educ..

[25] Wu D, Truhlar DG (2021). How accurate are approximate density functionals for noncovalent interaction of very large molecular systems?. J. Chem. Theory Comput..

[26] Zhao Y, Truhlar DG (2011). Applications and validations of the Minnesota density functionals. Chem. Phys. Lett..

[27] Crittenden DL (2009). A systematic CCSD(T) study of long-range and noncovalent interactions between benzene and a series of first- and second-row hydrides and rare gas atoms. J. Phys. Chem. A.

[28] Maurer LR, Bursch M, Grimme S, Hansen A (2021). Assessing density functional theory for chemically relevant open-shell transition metal reactions. J. Chem. Theory Comput..

[29] Chan B (2021). Assessment and development of DFT with the expanded CUAGAU-2 set of group-11 cluster systems. Int. J. Quantum Chem..

[30] Paier J (2006). Screened hybrid density functionals applied to solids. J. Chem. Phys..

[31] Ashcroft, N. W. & Mermin, N. D. *Solid State Physics* (Saunders College, 1976).

[32] Marder, M.P. *Condensed Matter Physics* (Wiley, 2000).

[33] Yu HS, Li SL, Truhlar DG (2016). Perspective: Kohn–Sham density functional theory descending a staircase. J. Chem. Phys..

[34] Chandrashekar G, Sahin F (2014). A survey on feature selection methods. Comput. Electr. Eng..

[35] Grimme S, Antony J, Ehrlich S, Krieg H (2010). A consistent and accurate ab initio parametrization of density functional dispersion correction (DFT-D) for the 94 elements H-Pu. J. Chem. Phys..

[36] Yu HS, Zhang W, Verma P, He X, Truhlar DG (2015). Nonseparable exchange–correlation functional for molecules, including homogeneous catalysis involving transition metals. Phys. Chem. Chem. Phys..

[37] Yu HS, He X, Truhlar DG (2016). MN15-L: A new local exchange-correlation functional for Kohn-Sham density functional theory with broad accuracy for atoms, molecules, and solids. J. Chem. Theory Comput..

[38] Verma P, Wang Y, Ghosh S, He X, Truhlar DG (2019). Revised M11 exchange–correlation functional for electronic excitation energies and ground-state properties. J. Phys. Chem. A.

[39] Wang Y (2020). M06-SX screened-exchange density functional for chemistry and solid-state physics. Proc. Natl Acad. Sci. USA.

[40] Goerigk L, Grimme S (2011). Efficient and accurate double-hybrid-meta-GGA density functionals—Evaluation with the extended GMTKN30 database for general main group thermochemistry, kinetics, and noncovalent interactions. J. Chem. Theory Comput..

[41] Settles B (2012). Active learning. Synth. Lectures Artif. Intell. Mach. Learn..

[42] Zhang L, Lin D-Y, Wang H, Car R, E W (2019). Active learning of uniformly accurate interatomic potentials for materials simulation. Phys. Rev. Mater..

[43] Schmidt J, Marques MRG, Botti S, Marques MAL (2019). Recent advances and applications of machine learning in solid-state materials science. NPJ Comput. Mater..

[44] Frisch, M.J. et al. Gaussian 16 revsion A.03 software. *Gaussian Inc.* https://gaussian.com/ (2016).

[45] Wang Y, Jin X, Yu HS, Truhlar DG, He X (2017). Revised M06-L functional for improved accuracy on chemical reaction barrier heights, noncovalent interactions, and solid-state physics. Proc. Natl Acad. Sci. USA.

[46] Liu, Y. et al. Supervised learning of a chemistry functional with damped dispersion. *Zenodo*10.5281/zenodo.7306137 (2022). 10.1038/s43588-022-00371-5 PMC10766516 38177952

