# Supplementary material for: Supervised learning of a chemistry functional with damped dispersion
Source: Nat Comput Sci. 2022 Dec 23;3(1):48–58. doi: 10.1038/s43588-022-00371-5 (PMC10766516; doi:10.1038/s43588-022-00371-5)
Supplement: Supplementary file 1 — Supplementary Sects. 1–3, Tables 1–22 and Figs. 1–4. [file 43588_2022_371_MOESM1_ESM.pdf]

nature computational science

Article https://doi.org/10.1038/s43588-022-00371-5

Supervised learning of a chemistry functional with damped dispersion

In the format provided by the authors and unedited

Contents

1. Additional details and references S-1
1.1 Optimization of the dispersion parameter $s_{r,6}$. S-1
1.2 Additional references S-2
2. Data sets details and results S-3
2.1 Dataset details and functional information S-4
2.2 Results for the GMTKN55 database S-11
2.3 Results for Minnesota Database 2019 S-19
2.4 Results for the MGCDB84 database S-20
2.5 Results for the DDB22 database S-24
2.5.1 Results for the GSE6075 in the DDB22 database S-25
2.5.2 Results for the EE157 dataset in the DDB22 database S-43
2.5.3 Results for the DM79 dataset in the DDB22 database S-46
2.5.4 Results for the MS261 dataset in the DDB22 database S-48
2.6 Results for the basis set superposition errors and grid errors S-50
2.7 Results for the ExL7, ROST61 and CUAGAU-2 datasets S-53
3. References cited in this file S-58

1. Additional details and references

1.1 Optimization of the dispersion parameter $s_{r,6}$

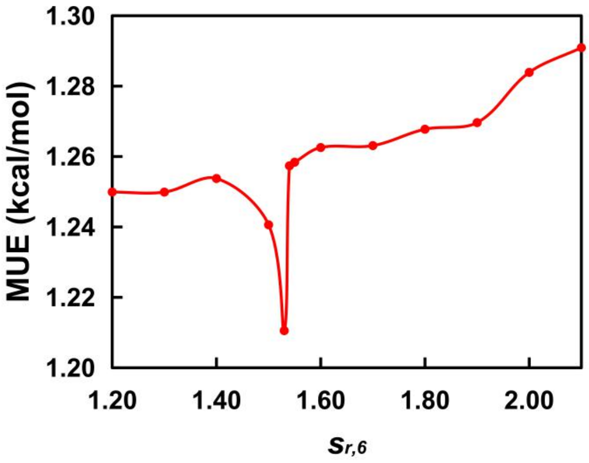

Supplementary Figure 1. Optimization of $s_{r,6}$ in CF22D.

The addition of a damped dispersion term constitutes a merger of density functional theory with molecular mechanics. Molecular mechanics has been used for more than 70 years, and one may cite many pioneering developers.[1-8] The ordinate of Supplementary Figure 1 represents the MUE (in kcal/mol) of the CF22D functional on the reference training dataset as a function of the single new parameter in our molecular mechanics term. Here, the reference training dataset includes 2501 data

points from the training dataset, namely, A21x12, A24, AlkAtom19, AlkIsomer11, AlkIsod14, Bauza30, Butanediol65, BzDC215, CT20, DIE60, DS14, EIE22, FmH2O10, ACONF, CYCONF, G21EA, G21IP, NBPRC, WATER27, H2O6Bind8, HB15, HW30, HW6Cl, HW6F, NC15, Pentane14, Shields38, SW49Rel345, SW49Bind345, SW49Rel6, SW49Bind6, TA13, BHPERI26, CR20, CRBH20, AlkBind12, HB49, Ionic43, H2O20Bind4, H2O20Rel4, H2O20Bind10, H2O20Rel10, H2O16Rel5, BSR36, HNBrBDE18, PlatonicTAE6, PlatonicIG6, PlatonicID6, PlatonicHD6, PX13, CE20, WCPT27, WCPT6, HAT707MR, HAT707nonMR, TAE140MR, TAE140nonMR, BDE99MR, BDE99nonMR, ISOMERIZATION20, SN13, BH76RC and DBH24. The x axis of Supplementary Figure 1 represents the parameter $s_{r,6}$ in the DFT-D3(0) form. We tested the values from 1.2 to 2.1 with an interval of 0.1, and we found that there was a significant drop in MUE between 1.5 and 1.6. Therefore, we further optimized the second decimal place of $s_{r,6}$ between 1.5 and 1.6 with an interval of 0.01, and finally the optimal value ($s_{r,6}$ = 1.53) was obtained, when it reached the lowest MUE for the reference training sets.

1.2 Additional references

Previous work provides background to the present work includes using big data and machine learning to improve energy functionals, either of the molecular mechanics type[9-20] or the density functional type[21-31]. The addition of molecular mechanics terms to density functionals was pioneered in older work,[32-34] but has been advanced in more recent work.[35, 36]

2. Data sets details and results

Supplementary tables and figures	Description	Page Number
**Sect. 2.1**	**Data sets details and functional information**	**S-4**
Table 1	Optimized parameters of CF22D.	S-6
Table 2	The superposition of databases.	S-7
Table 3	Functionals compared in this work.	S-10
**Sect. 2.2**	**Results for the GMTKN55 database**	**S-11**
Table 4	The MUEs (kcal/mol) for the GMTKN55 database.	S-14
Table 5	MUEs (kcal/mol) of selected representative functionals for subdatabases of the GMTKN55 Database	S-16
Table 6	WTMAD-1 (kcal/mol) for the GMTKN55 database.	S-17
Table 7	Mean of the absolute error (MoM in kcal/mol) for the GMTKN55 database.	S-18
**Sect. 2.3**	**Results for Minnesota Database 2019**	**S-19**
**Sect. 2.4**	**Results for the MGCDB84 database**	**S-20**
Table 8	MUEs (kcal/mol) of subdatabases of the MGCDB84 database	S-22
**Sect. 2.5**	**Results for the DDB22 database**	**S-24**
Table 9	MUEs (kcal/mol) of the GSE6075 subdatabase of the DDB22 database.	S-26
Table 10	MUEs (kcal/mol) for GSE6075 classified by four chemical properties: barrier heights (BH), noncovalent interactions (NC), isomerization energies (IE), and thermochemistry properties (TC).	S-28
Table 11	MUEs (kcal/mol) for main-group systems (MG) and systems containing transition metals (TM) of GSE6075 from the DDB22 database.	S-31
Table 12	MUEs (kcal/mol) for four transition metal test sets (functionals are ordered by the MUEs on TM_test107).	S-34
Table 13	MUEs (kcal/mol) of ωB97M-V, ωB97X-V and some doubly-hybrid functionals for transition metal test sets of CUAGAU42.	S-36
Table 14	MUEs (kcal/mol) for 'complex' and 'simple' datasets of GSE6075 from the DDB22 database.	S-37
Figure 2	The absolute error (kcal/mol) for the isomerization energy of C_8H_8 from the complex DC13 test set of the GMTKN55 database.	S-39
Table 15	MUEs (kcal/mol) for potential energy curves (PECs) in GSE6075 from the DDB22 database.	S-40
Table 16	MUEs (eV) for excitation energies with functionals sorted in order of increasing MUE on EE128.	S-43
Table 17	The MUEs (Debye) for the database of 79 dipole moments (DM79) and its two subdatabases.	S-46
Table 18	The MUEs (Å) of molecular structure database MS261 and its datasets.	S-48
**Sect. 2.6**	**Results for the basis set superposition errors (BSSEs) and grid errors**	**S-50**
Table 19	The average basis set superposition errors (BSSEs) of functionals (kcal/mol).	S-50
Figure 3	The average basis set superposition errors of functionals.	S-51
Figure 4	The grid errors of functionals.	S-52
**Sect. 2.7**	**Results for the ExL7, ROST61, and CUAGAU-2 datasets**	**S-53**
Table 20	Deviation of the binding energies of the ExL7 dataset for DFT Calculations.	S-53
Table 21	The MUEs (kcal/mol) for the ROST61 dataset.	S-55
Table 22	The MUEs (kcal/mol) for the CUAGAU-2 dataset	S-56

2.1 Data sets details and functional information

Data set details are given in the Supplementary Data 1 and 5.

Notes on Supplementary Data 1. This provides the composition of the combined database DDB22. MDB2019 denotes Minnesota database 2019. NC means noncovalent interactions; IE means isomerization energies; TC means thermochemistry; BH means barrier heights; EE means excitation energies; MS means molecular structures.

Training*: The datasets under this classification were used for training and belong to the ‘complete overlap’ portion defined in Supplementary Table 2. The datasets TAE140, G21EA, G21IP, NBPRC, BH76RC, BSR36, NHTBH38, HTBH38, BHPERI26, PX13, WATER27, H2O20Bind4, Ionic43, ACONF, Butanediol165 from MGCDB84 database were used for training, but the datasets W4-11, G21EA, G21IP, NBPRC, BH76RC, BSR36, BH76, BHPERI, PX13, WATER27, AHB21, CHB6, IL16, ACONF and BUT14DIOL from GMTKN55 database which are completely overlapped with the corresponding datasets from MGCDB84 were chosen in DDB22. Therefore, these datasets are classified into the training set of DDB22.

Training**: Only AE18 is included in this classification. AE17 in the Minnesota database 2019 was used for training, but in DDB22 we selected AE18 from MGCDB84, and this has one more datum than AE17. Thus, AE18 is also classified into the training set of DDB22.

Test*: The datasets under this classification belong to the ‘subset’ portion defined in Supplementary Table 2, in which most of the data points were not used for training. This category includes PA26 (26 data) and ISOL24 (24 data) from the GMTKN55 database and RG10 (569 data) from the MGCDB84 database. The PA8 (8 data), IsoL6/11 (6 data), and NGD21/18 (21 data) subdatabases of Minnesota database 2019 which were used for training are subsets of the PA26, ISOL24, and RG10, respectively. But they account for only a small portion of the PA26, ISOL24, and RG10 subdatabases, and thus the PA26, ISOL24 and RG10 are classified as Test* in this study.

VT denotes the datasets used for validation and training, including the W4-11, BH76RC, DBH24, ISOMERIZATION20, BDE99MR, BDE99nonMR, HAT707MR, HAT707nonMR, and SN13.

V denotes the datasets used for validation, including the S22, RG10, and NBC10.

Notes on Supplementary Data 5. Description of the training sets and validation datasets and the final inverse weights. Datasets 1 to 79 comprise the initial training set; datasets 80 to 89 are the sets added to the training set by supervised learning; datasets 90 to 92 are used only for testing. MDB2019 denotes Minnesota Database 2019; BH means barrier heights; NC means noncovalent interactions; IE means isomerization energies; TC means thermochemistry; TM means transition metal; EE means excitation energies.

Note about the differences between Supplementary Data 1 and 5. There are some slight differences between the databases and datasets in Supplementary Data 1 and 5. The training and validation databases in Supplementary Data 5 are from MDB2019 and MGCDB84, whereas the databases and datasets in Supplementary Data 1 constitute the entire DDB22 database.

The datasets in Supplementary Data 1 are reorganized from MDB2019, MGCDB84, GMTKN55, TMC34, and CUAGAU42. There is some overlap of data in MGCDB84, GMTKN55, and MDB2019 (see Supplementary Table 2), and some of the overlapped data differs in terms of reference energies and/or basis sets. Because the reference values are more accurate in GMTKN55, we selected the datasets in GMTKN55 in the final DDB22 database. The reader is referred to Supplementary Table 2 and the explanations in Methods Section of the article for more information about handling the overlap.

Another difference of Supplementary Data 1 and 5 is that in Supplementary Data 1, we grouped the datasets in four categories, namely, MDB2019, MGCDB84, GMTKN55, and others.

The papers cited in Supplementary Data 1 and 5 are consistent with this Supplementary Information.

Supplementary Table 1. Optimized parameters of CF22D. The parameters shown in the table correspond to the variables of equations 2, 3, 4 and 7 in the main text (namely, X in equation (2), a_{ijk} in equation (3), b_i and c_i in equation (4), and $s_{r,6}$ in equation (7)).

Nonseparable				Correlation	
a000	0.244161168	a102	1.646506623	b0	0.873863376
a001	-0.389728151	a103	-3.504641550	b1	0.078066142
a002	-1.829675858	a104	-3.922228074	b2	6.576550257
a003	1.396044771	a110	0.843718076	b3	-1.126030147
a004	2.315047133	a111	10.779373313	b4	-3.244797887
a005	0.397552547	a112	2.293612669	b5	-2.186090839
a010	1.082144406	a113	7.088363286	b6	-3.489135041
a011	-7.894560034	a120	2.598770741	b7	3.090689716
a012	-3.656253030	a121	-0.088522116	b8	3.866592474
a013	2.574496508	a122	7.180809030		
a014	4.031038406	a200	-1.017514009	c0	0.828203832
a020	-3.931389433	a201	1.735020310	c1	-2.518707202
a021	0.333519075	a202	3.499241561	c2	10.436806314
a022	-3.032270318	a203	0.922224945	c3	3.588267084
a023	3.673752289	a210	-2.212903920	c4	-5.789404145
a030	3.005997956	a211	0.243080429	c5	3.353560215
a031	-6.463733874	a212	17.306321840	c6	-2.432384384
a032	-4.596755225	a300	0.311402396	c7	-1.147183331
a100	0.964839180	a301	-3.257126009	c8	2.991316045
a101	0.363791944	a302	-3.372399742		
HF exchange				Damped dispersion	
HFX	0.462806			$s_{r,6}$	1.53

Supplementary Table 2. The superposition of databases. The number in parentheses to the right of the dataset represents the number of data points in the dataset.

	**GMTKN55**[133]	**MGCDB84**[179]	**MDB2019**[180]
		**complete overlap**	
**1**	**W4-11**(140)[103]	TAE140(140)	
**2**	**G21EA**(25)[84]	G21EA(25)	
**3**	**G21IP**(36)[37, 84]	G21IP(36)	
**4**	**NBPRC**(12)[37, 85, 86, 133]	NBPRC(12)	
**5**	**BH76RC**(30)[37, 133]	BH76RC(30)	
**6**	**BSR36**(36)[133]	BSR36(36)	
**7**	**BH76**(76)[37, 44, 45, 133]	HTBH38(38), NHTBH38(38)	HTBH38/18(38), NHTBH38/18(38)
**8**	**BHPERI**(26)[37, 38, 154-156]	BHPERI26(26)	
**9**	**AHB21**(21)[71], **CHB6**(6)[71], **IL16**(16)[71]	Ionic43(43)	
**10**	**ACONF**(15)[80]	ACONF(15)	
**11**	**PX13**(13)[41, 133]	PX13(13)	
**12**	**S22**(22)[50, 107]	S22(22)	
**13**	**S66**(66)[158]	S66(66)	
**14**	**MCONF**(51)[133, 168]	Melatonin52(52)	
**15**	**But14diol**(64)[77, 133]	Butanediol165(65)	
**16**	**WATER27**(27)[61, 181]	WATER27(23), H2O20Bind4(4)	
**17**		**EA13**(13)[140]	EA13/03(13)
		**subset**	
**18**	Amino20x4(80)	**YMPJ519**(519)[171]	
**19**	**PA26**(26)		PA8(8)
**20**	**ISOL24(24)**[133, 162]		IsoL6/11(6)
**21**		IP13(13)	**IP23**(23)[46, 95]
**22**		**AE18**(18)[176]	AE17(17)
**23**		**RG10**(569)[51]	NGD21/18(21)
**24**	**HAL59**(59)[133, 160, 161]	XB51(20)[160]	
		**partial overlap**	
**25**	**S22**(22)[50, 107]	S22(22)	**NCCE30/18**(30)[46-50]
**26**	**HAL59**(59)[133, 160, 161]	**X40**(31)[161], **XB18**(8)[160]	
**27**	**CDIE20**(20)[87, 133, 153]	**DIE60**(60)[78]	
**28**	**DC13**(13)[37, 133-144]	**C20C24**(8)[144], **Styrene45**(45)[141]	**DC9/19**(9)[46]

Notes on Supplementary Table 2: The datasets of DDB22 database come from the Minnesota database 2019, MGCDB84 database, GMTKN55 database, and transition metal datasets TMC34 and CUAGAU42. There are some overlaps between the three datasets, namely, the Minnesota database 2019, MGCDB84 database, GMTKN55 database. The degree of overlap of datasets are classified into three categories: ‘complete overlap’, ‘subset’ and ‘partial overlap’. As datasets from different sources may use different basis set, reference values and structural

coordinate information, dataset sources in boldface were chosen in DDB22.

Lines 1~17 denote the 'complete overlap' category. Data sets from different databases describe exactly the same structural properties. In this category, datasets from the GMTKN55 database are selected. The coordinates, basis set and reference energies are the same as those in the GMTKN55 database.

Lines 18~24 denote the 'subset'. Some datasets in one database are subsets of other datasets from another database. For different databases, datasets with more data points are selected into the DDB22 database, with boldface. The coordinates, basis sets, and reference energies used in calculations came from those of the source database to which the dataset belongs. In line 24, XB51 from the GMTKN55 database is the subset of HAL59 from MGCDB84 database, except the $Li+Br_2 \rightarrow LiBr_2$ in XB51. However, there is only one exception. We still classify XB51 as a subset of HAL59 in this study.

Lines 25~28 denote the 'partial overlap'. Some datasets from one database have only a few data points overlapping with the other datasets from another database, so all of them are included in the DDB22 database in this study. The calculations were performed based on the coordinates, basis sets and reference energies from the respective original databases.

Line 25, NCCE30/18 from the Minnesota database 2019 includes 30 data points, 8 of which are the same as those from S22 in GMTKN55 database and MGCDB84 database, namely, T-shaped $(C_6H_6)_2$, $(NH_3)_2$, $(H_2O)_2$, $(HCOOH)_2$, $(HCONH_2)_2$, $(CH_4)_2$, $(C_2H_2)_2$, parallel-displaced $(C_6H_6)_2$; Line 26, X40 and XB18 from the MGCDB84 database include the same data points overlapped with the HAL59 from GMTKN55, namely, XB18: $FBr+HCN \rightarrow FBrNCH$, $Br_2+H_2CO \rightarrow Br_2OCH_2$; X40: $C_6H_3F_3+C_6H_6 \rightarrow C_6H_3F_3\cdots C_6H_6$, $C_6F_6+C_6H_6 \rightarrow C_6F_6\cdots C_6H_6$, $C_6H_5Cl+CH_3COCH_3 \rightarrow C_6H_5Cl\cdots CH_3COCH_3$, $C_6H_5Br+CH_3COCH_3 \rightarrow C_6H_5B\cdots CH_3COCH_3$, $C_6H_5Cl+N(CH_3)_3 \rightarrow C_6H_5Cl\cdots N(CH_3)_3$, $C_6H_5Br+N(CH_3)_3 \rightarrow C_6H_5Br\cdots N(CH_3)_3$, $C_6H_5Br+HS\text{-}CH_3 \rightarrow C_6H_5Br\cdots HS\text{-}CH_3$, $C_6H_6+CH_3\text{-}Br \rightarrow C_6H_6\cdots CH_3\text{-}Br$, $C_6H_6+CF_3\text{-}Br$

→$C_6H_6\cdots CF_3$-Br; Line 27, there are 16 reactions in CDIE20 from the GMTKN55 database, which are the same as those in DIE60 from the MGCDB84 database. Overlapped reactions in CDIE20 are R20→P20, R22→P22, R25→P25, R26→P26, R40→P40, R43→P43, R44→P44, R45→P45, R46→P46, R47→P47, R48→P48, R49→P49, R51→P51, R52→P52, R56→P56, R60→P60; Line 28, DC13 from the GMTKN55 database includes 1 system, the same as that in DC9/19 from the Minnesota Database 2019, which is C_6H_6+6Cl_2→C_6Cl_6+6HCl, and other reactions are different; DC13 from GMTKN55 includes 1 system, the same as that in C20C24 from the MGCDB84 database, which is C20CageD2h→C20BowlC5v, and other reactions are different; DC13 from the GMTKN55 database includes 1 system, the same as that in Styrene45 from the MGCDB84 database, which is the 41st reaction in Karton and Martin's study on 45 isomerization energies[141], and other reactions are different.

Database overlap. When the datasets from different databases describe the same reaction or properties, i.e., when they are completely overlapped, the data from GMTKN55 database is chosen in DDB22 (which means the reference values, structures, basis sets and detailed calculation methods were used in the same way as those in GMTKN55). When a dataset is a subset of another dataset from another database, the dataset with more data points is included in the DDB22 database (the reference values, structures, basis sets and detailed calculation methods were used in the same way as those in their original database). When the datasets from different databases have only a few data points overlapping with one another, this is classified as partial overlap, and both datasets are included in the DDB22 database.

Supplementary Table 3. Functionals compared in this work.

Functional	Ref(s).	Type	Damped dispersion
**Local functionals**			
PBE	182	GGA	none
PBE-D3(BJ)	182	GGA	D3(BJ)[183]
TPSS	184	meta-GGA	none
TPSS-D3(BJ)	184	meta-GGA	D3(BJ)[183]
M06-L	185	meta-GGA	none
M06-L-D3(0)	185	meta-GGA	D3(0)[186]
MN15-L	187	meta-NGA	none
revM06-L	188	meta-GGA	none
SCAN-D3(0)	189	meta-GGA	D3(0)[190]
**Range-separated hybrid functionals**			
ωB97X-D	191	range-separated hybrid GGA	D2[192]
M11	193	range-separated hybrid meta-GGA	none
M11-D3(BJ)	193	range-separated hybrid meta-GGA	D3(BJ)[194]
revM11	195	range-separated hybrid meta-GGA	none
M11plus	196	range-separated hybrid meta-GGA	none
ωB97X-V	197	range-separated hybrid GGA	VV10[197]
ωB97M-V	198	range-separated hybrid meta-GGA	VV10[198]
M06-SX	199	range-separated hybrid meta-GGA	none
DM21	30	range-separated hybrid meta-GGA	D3(BJ)[183]
**Global hybrid functionals**			
B3LYP	200, 201	global hybrid GGA	none
B3LYP-D3(BJ)	200, 201	global hybrid GGA	D3(BJ)[183]
PBE0	202, 203	global hybrid GGA	none
PBE0-D3(BJ)	202, 203	global hybrid GGA	D3(BJ)[183]
M05-2X	204	global hybrid meta-GGA	none
M05-2X-D3(0)	204	global hybrid meta-GGA	D3(0)[186]
PW6B95-D3(BJ)	205	global hybrid meta-GGA	D3(BJ)[183]
M06-2X	143	global hybrid meta-GGA	none
M06-2X-D3(0)	143	global hybrid meta-GGA	D3(0)[186]
M06	143	global hybrid meta-GGA	none
M06-D3(0)	143	global hybrid meta-GGA	D3(0)[186]
M08-HX	206	global hybrid meta-GGA	none
MN15	207	global hybrid meta-NGA	none
MN15-D3(BJ)	207	global hybrid meta-NGA	D3(BJ)[133]
revM06	208	global hybrid meta-GGA	none
CF22D	present	global hybrid meta-NGA	D3(0)[35]
**Doubly hybrid functionals**			
DSD-BLYP-D3(BJ)	186, 209		D3(BJ)[186]
B2GPPLYP-D3(BJ)	102, 186		D3(BJ)[186]
B2PLYP-D3(BJ)	134, 186		D3(BJ)[186]
MPW2PLYP-D3(BJ)	133, 210		D3(BJ)[133]
PWPB95-D3(BJ)	86, 186		D3(BJ)[186]

2.2 Results for the GMTKN55 database

The results for the GMTKN55 database were analyzed by four schemes in this study. This work focused on the comparison of the MUE results, the same as the comparison for other databases. The WTMAD-1 and WTMAD-2 results were also utilized for comparison of the performance of functionals by adding a "difficulty factor" for each subdatabase in GMTKN55.[133] The MoM (the mean of the mean absolute errors for 55 subdatabases) results use the comparison metric defined in the DM21 paper.[30]

$$\mathrm{MUE} = \frac{1}{\sum_{i}^{55} n_i} \cdot \sum_{i}^{55} n_i \mathrm{MAD}_i$$

(Supplementary Equation 1)

$$\text{WTMAD-1} = \frac{1}{55} \cdot \sum_{i}^{55} w_i \mathrm{MAD}_i$$

(Supplementary Equation 2)

$$\text{WTMAD-2} = \frac{1}{\sum_{i}^{55} n_i} \cdot \sum_{i}^{55} n_i \cdot \frac{56.84\mathrm{kcal\ mol^{-1}}}{\overline{|\Delta E|}_i} \cdot \mathrm{MAD}_i$$

(Supplementary Equation 3)

$$\mathrm{MoM} = \frac{1}{55} \cdot \sum_{i}^{55} \mathrm{MAD}_i$$

(Supplementary Equation 4)

where n_i in Supplementary Equation 1 and 3 is the number of relative energies; MAD_i in Supplementary Equation 1 to 4 is the mean absolute error for each subdatabase; $\overline{|\Delta E|}_i$ in Supplementary Equation 3 is the average relative absolute energies given in Grimme and co-workers' work[133]; w_i in Supplementary Equation 2 is a weighting of 0.1 if $\overline{|\Delta E|}_i > 75$ kcal/mol, and 10 if $\overline{|\Delta E|}_i < 7.5$ kcal/mol.

For analyzing the results for the GMTKN55 database, the performance of 35 selected functionals was mainly compared (see Tables 1 and 2 for a list of these functionals). Results for CF22D and ωB97X-D functionals are newly calculated in this work. Results for M11plus are obtained from the ref. 211, results for ωB97M-V are obtained from the ref. 212, results of DM21 are obtained from the ref. 30, and

results for the doubly hybrid functionals and all the remaining functionals are obtained from the ref. 133.

The comparisons are in Supplementary Data 2, 3 and Supplementary Tables 4-7. The values highlighted in bold in those tables are in each case the five best-performing functionals but excluding for this purpose the doubly-hybrid functionals and DM21. Thus, these are the five best performing functionals of the other 29 functionals included in the GMTKN55 comparisons.

Supplementary Table 4 gives the mean unsigned errors (MUEs) for each of the seven subdatabases and for the full GMTKN55. (Errors measured in two other ways proposed by Goerigk et al. are given in Supplementary section 2.2; they lead to similar conclusions.) Supplementary Data 3 gives the MUEs on the 55 individual datasets.

Since the doubly hybrid functionals are more expensive than the other functionals and the recent deep learning functional DM21 is quite different from the other functionals, we first compare only the 29 other functionals in Supplementary Table 4; for brevity we call these ordinary functionals.

As shown in Supplementary Table 4, CF22D gives the best performance out of the ordinary functionals on the whole database with an MUE of 1.45 kcal/mol, which is 0.37 kcal/mol smaller than the MUE given by ωB97M-V (the second best). The B3LYP[201, 213-215] and PBE[182] DFAs are among the most widely used functionals for chemistry and physics, respectively, but their overall computational accuracy is lower than many more recently developed functionals. The MUEs of PBE and B3LYP for GMTKN55 are 5.16 and 5.08 kcal/mol, respectively. In stark contrast, the MUEs of the more recently developed ωB97M-V,[198] M08-HX,[206] and M06-2X[143] for GMTKN55 are all lower than 2 kcal/mol.

For the whole GMTKN55 database, MN15-D3(BJ) does not shows any improvement over MN15, which shows the insufficiency of adding a damped dispersion term without reoptimizing the rest of the functional. Supplementary Table 4 shows that M08-HX, having the third best MUE (1.91 kcal/mol), is the best performing ordinary functional without dispersion corrections; however, CF22D gives a lower MUE than M08-HX for 40 out of 55 datasets. Supplementary Table 4 shows

that, among the ordinary functionals, CF22D is the best functional for the ‘small’ (2.24 kcal/mol) and ‘large’ (2.73 kcal/mol) subdatabases, the second-best functional for the ‘BH’ (1.28 kcal/mol) and ‘intra-NC’ (0.27 kcal/mol) subdatabases, and the third best for the ‘inter-NC’ (0.46 kcal/mol) subdatabase. Whereas CF22D is in the top three functionals for all five of these subdatabases, no other ordinary functional is among the eight best-performing functionals for all of them; this demonstrates the high across-the-board accuracy CF22D for various chemical properties of systems containing main-group elements.

CF22D has the second-best performance for Radical7 and the best performance for Nonradical48, for an average rank of 1.5. No other functional has an average rank lower than 3.5. This demonstrates an unusually good ability of CF22D to perform well on both open-shell and closed-shell species.

The top five rows of Supplementary Table 4 show that adding nonlocal correlation based on unoccupied orbitals can improve the results, which is an example of the general phenomenon[211, 216] that adding ingredients is a powerful way to improve functionals. However, only one of the five doubly hybrid functionals has an MUE lower than CF22D by more than 0.07 kcal/mol, and two have higher MUEs. Supplementary Table 4 shows that the recent deep learning functional DM21 has performance even slightly better on average than CF22D. It is very encouraging that the deep learning and supervised learning functionals do so well in comparison to other state-of-the-art functionals.

Supplementary Table 4. The MUEs (kcal/mol) for the GMTKN55 database.

MUE	Small	Large	BH	Inter	Intra	Radical	Nonradical	<Rank>	Overall
**Doubly hybrid functionals**									
DSD-BLYP-D3(BJ)	1.98	2.57	1.13	0.36	0.19	2.01	1.21		1.29
PWPB95-D3(BJ)	2.07	2.38	1.21	0.63	0.33	2.36	1.26		1.38
B2GPPLYP-D3(BJ)	2.08	2.89	1.18	0.48	0.19	1.97	1.34		1.41
B2PLYP-D3(BJ)	2.16	4.60	1.81	0.45	0.23	2.31	1.73		1.79
MPW2PLYP-D3(BJ)	2.34	5.63	1.52	0.77	0.24	2.29	2.01		2.04
**Deep-learning functional**									
DM21	2.24	2.32	1.33	0.49	0.25	2.10	1.31		1.40
**Ordinary functionals**									
CF22D	**2.24**	**2.73**	**1.28**	**0.46**	**0.27**	**2.66**	**1.31**	**1.5**	**1.45**
ωB97M-V	2.98	**3.77**	**1.29**	**0.30**	**0.27**	3.35	**1.64**	**7.5**	**1.82**
M08-HX	**2.72**	4.23	**1.27**	0.58	0.49	**2.67**	**1.82**	**3.5**	**1.91**
PW6B95-D3(BJ)	2.87	**3.43**	2.30	**0.52**	0.40	3.61	**1.73**	10.5	**1.93**
M06-2X	**2.73**	**4.21**	1.89	0.62	0.36	2.95	**1.86**	**6**	**1.97**
M06-2X-D3(0)	**2.72**	**4.20**	1.89	0.67	0.40	**2.94**	1.88	**5.5**	1.99
MN15	**2.75**	4.70	**1.52**	**0.52**	0.59	3.05	1.92	9.25	2.04
MN15-D3(BJ)	**2.75**	4.71	**1.52**	0.53	0.59	3.04	1.92	8.75	2.04
M11-D3(BJ)	2.96	4.94	1.62	0.59	0.74	3.03	2.10	8.75	2.20
M11	2.99	5.66	1.65	0.84	0.64	3.03	2.28	9.25	2.36
M05-2X	3.16	5.83	1.89	0.90	**0.34**	**2.94**	2.36	8.25	2.43
M05-2X-D3(0)	3.16	5.88	1.92	0.92	**0.33**	**2.94**	2.38	9	2.44
ωB97X-D	3.00	7.14	**1.46**	0.53	0.38	3.36	2.36	12.75	2.46
ωB97X-V	2.96	7.56	1.56	**0.36**	**0.25**	3.14	2.39	13	2.47
M11plus	3.06	7.02	1.84	0.95	0.37	**2.40**	2.62	9	2.60
M06-D3(0)	3.29	7.22	1.94	**0.52**	0.58	3.40	2.58	15.75	2.67
PBE0-D3(BJ)	3.90	4.46	3.71	1.05	**0.34**	4.18	2.52	17.5	2.70
M06	3.32	7.86	1.98	0.74	0.40	3.40	2.72	16.75	2.79
B3LYP-D3(BJ)	3.73	6.74	3.24	0.75	0.36	4.14	2.75	19	2.90
PBE0	3.82	5.60	3.24	1.42	1.08	4.19	2.88	20.5	3.02
MN15-L	3.75	5.68	2.02	2.19	1.44	3.53	3.02	19.5	3.08
SCAN-D3(0)	4.83	4.48	5.43	1.61	0.41	6.22	3.00	25	3.35
TPSS-D3(BJ)	5.27	7.36	6.19	0.94	0.48	4.91	3.80	23.5	3.92
M06-L-D3(0)	4.71	14.43	2.52	0.54	0.64	4.98	4.29	24.75	4.37
M06-L	4.72	14.67	2.52	0.69	0.58	4.98	4.36	25.25	4.43
PBE-D3(BJ)	8.26	6.80	7.12	1.51	0.53	6.03	4.89	27	5.02
B3LYP	4.66	14.62	3.30	2.56	1.60	4.46	5.15	25.5	5.08
TPSS	5.26	12.75	5.47	2.13	1.39	4.90	5.14	25.5	5.11
PBE	8.07	7.75	6.51	1.53	1.18	5.93	5.07	27	5.16
Average	3.82	6.77	2.76	0.95	0.60	3.80	2.85		2.96

Notes on Supplementary Table 4. The 'Small' denotes the basic properties and reaction energies for small systems, which consist of W4-11, G21EA, G21IP, DIPCS10, PA26, SIE4x4, ALKBDE10, YBDE18, AL2X6, HEAVYSB11, NBPRC, ALK8, RC21, G2RC, BH76RC, FH51, TAUT15, DC13 datasets; The 'Large' denotes reaction energies for large systems and isomerization energies, which consist of MB16-43, DARC, RSE43, BSR36, CDIE20, ISO34, ISOL24, C60ISO, PArel datasets; The 'BH' denotes reaction barrier heights, which consist of BH76, BHPERI, BHDIV10, INV24, BHROT27, PX13, WCPT18 datasets; The 'Inter' denotes the intermolecular noncovalent interactions, which consist of RG18, ADIM6, S22, S66, HEAVY28, WATER27, CARBHB12, PNICO23, HAL59, AHB21, CHB6, IL16 datasets; The 'Intra' denotes the intramolecular noncovalent interactions, which consist of IDISP, ICONF, ACONF, AMINO20x4, PCONF21, MCONF, SCONF, UPU23, BUT14DIOL datasets; The 'Overall' denotes the overall performance on the 55 datasets from the GMTKN55 database.

The 'Radical7' denote the datasets emphasizing radical species, which consist of G21EA, G21IP, SIE4x4, ALKBDE10, HEAVYSB11, RC21, RSE43 datasets. This classification method was taken from the ref. 211; The 'Nonradical48' denotes the remaining 48 datasets. This classification method was taken from the ref. 211.

The first five functionals are doubly-hybrid functionals and the sixth is the recent deep learning DM21 functional. The values in bold represent the five best-performing functionals of the remaining 29 functionals, which are denoted for convenience as the ordinary functionals. The average value in the last row also excludes the first six functionals. The <rank> column gives the average rank of ordinary functionals on the two preceding columns. In computing ranks, if two or more functionals have the same MUE to three significant figures, their ranks are averaged.

Supplementary Table 5. MUEs (kcal/mol) of selected representative functionals for subdatabases of the GMTKN55 Database.

	Small	Large	BH	Inter-NC	Intra-NC	Overall
**CF22D**	2.24	2.73	1.28	0.46	0.27	1.45
**ωB97M-V**	2.98	3.77	1.29	0.30	0.27	1.82
**PW6B95-D3(BJ)**	2.87	3.43	2.30	0.52	0.40	1.93
**M06-2X**	2.73	4.21	1.89	0.62	0.36	1.97
**M06-2X-D3(0)**	2.72	4.20	1.89	0.67	0.40	1.99
**MN15**	2.75	4.70	1.52	0.52	0.59	2.04
**MN15-D3(BJ)**	2.75	4.71	1.52	0.53	0.59	2.04
**ωB97X-D**	3.00	7.14	1.46	0.53	0.38	2.46
**ωB97X-V**	2.96	7.56	1.56	0.36	0.25	2.47
**PBE0-D3(BJ)**	3.90	4.46	3.71	1.05	0.34	2.70
**B3LYP-D3(BJ)**	3.73	6.74	3.24	0.75	0.36	2.90
**SCAN-D3(0)**	4.83	4.48	5.43	1.61	0.41	3.35
**PBE-D3(BJ)**	8.26	6.80	7.12	1.51	0.53	5.02

Worst … *Best*

Note on Supplementary Table 5. These categories are similar to Supplementary Table 4.

Supplementary Table 6. WTMAD-1 (kcal/mol) for the GMTKN55 database. These categories are similar to Supplementary Table 4 but for WTMAD-1. The first five functionals are doubly-hybrid functionals and the sixth is the recent deep learning DM21 functional. The values in bold represent the five best-performing functionals of the remaining 29 functionals, which are denoted for convenience as the ordinary functionals.

WTMAD-1	Small	Large	BH	Inter	Intra	Radical	Nonradical	Overall
DSD-BLYP-D3(BJ)	1.63	2.22	1.45	1.94	1.87	1.81	1.82	1.81
B2GPPLYP-D3(BJ)	1.72	2.44	1.60	2.16	1.90	1.80	1.97	1.95
B2PLYP-D3(BJ)	2.24	3.36	2.02	1.86	2.20	2.19	2.31	2.30
MPW2PLYP-D3(BJ)	2.24	3.36	1.77	2.23	2.23	2.18	2.39	2.36
PWPB95-D3(BJ)	2.08	2.53	1.50	1.75	2.95	2.18	2.12	2.15
DM21	1.84	2.09	1.35	2.20	2.40	1.82	2.04	1.99
ωB97M-V	**2.25**	**2.16**	**1.54**	**1.33**	**2.60**	2.83	**1.88**	**2.00**
CF22D	**2.39**	**2.41**	**1.43**	**1.94**	**2.28**	**2.49**	**2.11**	**2.15**
ωB97X-V	**2.63**	3.20	**1.91**	**1.45**	**2.29**	2.85	**2.22**	**2.32**
M05-2X-D3(0)	2.82	**2.62**	2.97	2.46	**2.85**	2.57	**2.77**	**2.73**
M06-2X	**2.66**	3.04	2.41	2.38	3.64	3.10	2.86	**2.79**
M06-2X-D3(0)	**2.66**	2.96	2.40	**2.21**	4.03	3.08	2.87	2.80
PW6B95-D3(BJ)	3.00	3.99	2.47	**2.01**	3.29	3.38	**2.83**	2.93
ωB97X-D	2.98	**2.88**	**1.91**	2.99	3.66	3.16	2.89	2.94
M08-HX	**2.48**	2.90	**1.99**	3.22	4.76	**2.33**	3.12	3.02
M05-2X	2.84	**2.78**	2.96	3.63	3.21	2.59	3.16	3.08
M11plus	3.20	**2.88**	2.63	2.89	3.81	**1.85**	3.24	3.11
M06	3.23	4.33	2.67	3.29	3.55	3.16	3.41	3.41
MN15	3.00	3.31	2.21	3.21	5.50	3.06	3.49	3.41
MN15-D3(BJ)	3.00	3.31	2.21	3.27	5.50	3.06	3.51	3.42
B3LYP-D3(BJ)	3.85	5.02	3.12	2.75	**3.20**	4.03	3.53	3.60
M11-D3(BJ)	2.94	3.17	2.70	2.82	7.17	**2.48**	3.74	3.62
M06-D3(0)	3.15	4.02	2.59	3.64	5.55	3.17	3.76	3.72
M11	2.92	3.37	2.74	3.96	6.29	**2.48**	3.90	3.75
PBE0-D3(BJ)	3.74	4.26	4.42	3.86	3.30	3.75	3.83	3.87
SCAN-D3(0)	4.78	4.60	6.16	4.28	3.71	5.52	4.56	4.64
M06-L	4.46	7.92	3.31	3.45	5.22	4.45	4.79	4.78
M06-L-D3(0)	4.42	7.79	3.31	3.41	5.93	4.44	4.87	4.86
TPSS-D3(BJ)	4.76	6.15	6.28	4.15	4.74	4.84	5.02	5.04
PBE-D3(BJ)	5.29	5.93	7.62	5.40	5.04	5.93	5.60	5.67
PBE0	3.75	5.09	3.93	9.55	10.65	3.87	6.69	6.39
MN15-L	3.42	6.15	3.51	8.95	13.67	3.41	7.23	6.76
PBE	5.44	7.32	6.98	10.17	11.68	5.86	8.23	8.00
TPSS	5.17	8.32	5.56	13.20	13.59	4.91	9.34	8.87
B3LYP	5.32	7.84	3.23	15.55	15.27	4.65	9.97	9.33
Average	3.54	4.47	3.35	4.53	5.72	3.56	4.33	4.24

Supplementary Table 7. Mean of the absolute error (MoM in kcal/mol) for the GMTKN55 database. These categories are similar to Supplementary Table 4 but for MoM. The first five functionals are doubly-hybrid functionals and the sixth is the recent deep learning DM21 functional. The values in bold represent the five best-performing functionals of the remaining 29 functionals, which are denoted for convenience as the ordinary functionals.

MoM	Small	Large	BH	Inter	Intra	Radical	Nonradical	Overall
DSD-BLYP-D3(BJ)	2.02	2.69	8.10	0.47	2.60	2.42	1.25	1.40
B2GPPLYP-D3(BJ)	2.06	2.79	8.25	0.58	2.75	2.43	1.32	1.46
B2PLYP-D3(BJ)	2.51	4.21	11.79	0.51	3.74	2.89	1.76	1.90
MPW2PLYP-D3(BJ)	2.56	4.65	9.53	0.84	4.51	2.89	1.91	2.04
PWPB95-D3(BJ)	2.41	2.17	7.60	0.71	3.90	2.83	1.32	1.51
DM21	2.26	2.70	7.75	0.52	3.39	2.60	1.34	1.50
CF22D	**2.73**	**2.37**	**7.89**	**0.47**	**3.58**	**3.27**	**1.35**	**1.59**
ωB97M-V	3.04	**3.60**	**8.80**	**0.36**	**3.88**	3.97	**1.59**	**1.89**
PW6B95-D3(BJ)	3.29	**2.96**	12.25	0.63	5.57	4.25	**1.70**	**2.02**
M08-HX	**2.89**	3.71	**10.13**	0.76	6.97	**3.26**	1.85	**2.03**
MN15	3.13	**3.54**	11.15	**0.56**	8.17	3.78	1.83	**2.08**
MN15-D3(BJ)	3.13	**3.55**	11.15	**0.56**	8.19	3.78	1.83	**2.08**
M06-2X	**3.03**	3.72	13.60	0.70	5.00	4.05	**1.80**	2.09
M06-2X-D3(0)	**3.02**	3.70	13.58	0.74	5.49	4.04	**1.82**	2.10
M05-2X-D3(0)	3.09	4.87	16.65	1.01	**3.80**	3.58	2.23	2.40
M05-2X	3.11	4.89	16.50	1.01	4.70	3.60	2.25	2.42
M11-D3(BJ)	3.19	4.90	12.97	0.76	9.74	**3.50**	2.27	2.42
ωB97X-V	3.09	6.56	**10.55**	**0.43**	**4.39**	3.75	2.26	2.45
ωB97X-D	3.52	5.61	**9.87**	0.59	5.66	4.04	2.26	2.48
M11	3.17	5.40	13.23	0.93	8.67	**3.50**	2.38	2.52
M11plus	**2.95**	5.92	15.78	1.05	5.08	**2.69**	2.52	2.54
M06-D3(0)	3.71	5.36	12.12	0.64	8.67	4.01	2.41	2.61
M06	3.79	5.87	12.47	0.72	6.58	4.00	2.51	2.70
PBE0-D3(BJ)	4.12	3.63	25.71	1.10	**4.36**	4.95	2.40	2.73
B3LYP-D3(BJ)	4.22	5.55	18.16	0.80	6.09	5.05	2.59	2.90
PBE0	4.11	4.47	22.22	1.43	18.14	5.10	2.84	3.12
MN15-L	4.27	4.81	16.75	2.30	19.10	4.43	3.18	3.34
SCAN-D3(0)	5.72	3.90	35.72	1.54	5.54	8.59	2.87	3.60
TPSS-D3(BJ)	5.07	6.37	39.21	0.93	7.16	5.89	3.44	3.75
M06-L-D3(0)	5.11	10.98	14.35	0.67	11.03	5.74	3.83	4.08
M06-L	5.13	11.18	14.38	0.74	10.43	5.75	3.88	4.12
PBE-D3(BJ)	6.00	5.97	49.08	1.48	7.02	7.23	3.85	4.28
PBE	6.11	7.09	44.62	1.50	20.21	7.16	4.30	4.66
TPSS	5.44	10.51	34.04	2.07	23.80	5.96	4.86	5.00
B3LYP	5.73	11.40	19.04	2.52	28.52	5.69	5.08	5.15
Average	3.96	5.60	18.69	1.00	9.16	4.64	2.69	2.94

2.3 Results for Minnesota Database 2019

We use 29 functionals to assess the performance of functionals on Minnesota Database 2019. The MUEs for the AME418 subdatabase are given in Supplementary Data 4. The MUEs (kcal/mol) of selected functionals for the 26 energetic datasets of AME418 are given in Supplementary Data 6.

Results for CF22D, B3LYP-D3(BJ), M05-2X, M05-2X-D3(0), M06-D3(0), M06-2X-D3(0), M06-L-D3(0), M11-D3(BJ), MN15-D3(BJ), PBE-D3(BJ), PBE0-D3(BJ), and TPSS-D3(BJ) were calculated in this work. All other results were obtained from ref. 199.

The values highlighted in bold are the five smallest MUEs for each column.

The SR-MGM-BE8, SR-MGN-BE107, MR-MGM-BE4, and MR-MGN-BE17 databases constitute the MGBE136 subdatabase of main-group bond energies.

The SR-TML-BE11, MR-TML-BE12, SR-TMD-BE4, and MR-TMD-BE3 databases constitute the TMBE30 subdatabase of transition-metal bond energies.

The HTBH38/18 and NHTBH38/18 databases constitute the BH76/18 subdatabase of barrier heights. The NGD21/18 and NCCE30/18 databases constitute the NC51/18 subdatabase of noncovalent interaction energies.

The 3dEE8, 4dAEE5, and pAEE5 databases constitute the EE18 subdatabase of electronic excitation energies.

The 2pIsoE4, 4pIsoE4, and IsoL6/11 databases constitute the IsoE14 subdatabase of isomerization energies of large molecules.

The HC7/11 and πTC13 databases constitute the HCTC20 subdatabase of hydrocarbon energetics.

The EA13/03, PA8, IP23, AE17, SMAE3/19 and DC9/19 databases constitute the Misc73 subdatabase of miscellaneous data.

The 53 multi-reference systems as classified by the generalized B1 diagnostics constitute the MR53 subdatabase.[207, 208] The MR53 database contains all the systems in MR-MGM-BE4; MR-MGN-BE17; MR-TML-BE12; MR-TMD-BE3; it contains O + HCl → OH + Cl (fwd) in HTBH38/18 and H + N_2O → OH + N_2 (rev), H + F_2 →

HF + F (rev), CH_3 + FCl → CH_3F + Cl (rev) in NHTBH38/18; it contains E31-E1, DE (reaction c), and DE (reaction d) in HC7/11; it contains all systems except H_2S_2 in SMAE4; and it contains all systems except HCN···BF_3 → HCN + BF_3 in DC9/19.

The 297 single-reference systems defined by generalized B1 diagnostics constitute the SR297 subdatabase.[207, 208] It contains all the systems in subdatabases SR-MGM-BE8, SR-MGN-BE107, SR-TML-BE11, SR-TMD-BE4, 3dEE8, 4dAEE5, pAEE5, 4pIsoE4, 2pIsoE4, IsoL6/11, πTC13, EA13/03, PA8, IP23; it contains H_2S_2 in SMAE4; it contains HCN···BF_3 → HCN + BF_3 in DC9/19; it contains all systems except O + HCl → OH + Cl (fwd) in HTBH38/18; it contains all systems except H + N_2O → OH + N_2 (rev), H + F_2 → HF + F (rev), and CH_3 + FCl → CH_3F + Cl (rev) in NHTBH38/18; and it contains all systems except E31-E1, DE (reaction c), and DE (reaction d) in HC7/11.

2.4 Results for the MGCDB84 database

For assessing the performance of the functionals on the MGCDB84 database, 27 functionals are used for comparison (see Tables 1 and 2). For the MGCDB84 database, results of CF22D, MN15-D3(BJ) were newly calculated from this study, and all the results of other functionals for comparison were obtained from ref. 179. The basis set and integration grid were chosen as def2-QZVPPD and (99, 590), respectively, except for AE18 and RG10, for which the (500, 974) grid was used. Coordinates and reference values are taken from ref. 179, and all settings were consistent with ref. 179. The values highlighted in bold in the following table represent the five smallest overall MUEs on MGCDB84. The MUEs (kcal/mol) of the selected functionals for the 84 datasets of MGCDB84 database are given in Supplementary Data 7.

CF22D gives the best performance on the NCD, TCE, and TCD subdatabases with MUEs of 0.50, 1.74, and 3.36 kcal/mol, respectively, whereas ωB97M-V gives the second-best results for these three subdatabases, with the MUEs of 0.54, 1.75, and 3.39 kcal/mol, respectively. For the DIE and BH subdatabases, CF22D gives the second-best results, with MUEs of 1.44 and 1.37 kcal/mol, respectively; ωB97M-V is the best-performing functional for these two subdatabases, with MUEs of 1.14 and 1.24 kcal/mol, respectively. In addition, CF22D also gives the third-best result for EIE

and the fourth best results for the NCED and NCEC subdatabases. As shown in Supplementary Table 8, CF22D is among the five best performing functionals for all eight subdatabases, which indicates that CF22D achieves high accuracy for diverse chemical properties in the MGCDB84 database. The MN15-L,[187] revM06-L,[188] MN15, revM06,[208] and M06-SX[199] functionals were all developed with the goal of being universal functionals, that is, functionals with the broadest possible accuracy, and these DFAs are indeed able to achieve good accuracy (relative to many earlier functionals) for a broad range of properties, such as barrier heights and reaction energies for both main-group elements and transition metals. But none of these functionals has as broad an accuracy as CF22D. The ωB97M-V functional, despite its overall good behavior, also shows weak spots, for example, its performance for radicals in the GMTKN55 database is relatively poor with an MUE of 3.35 kcal/mol, and its MUE for the large-systems dataset C60ISO of relative energies between C_{60} isomers (C60ISO[163]) is 11.47 kcal/mol, while MN15,[207] PW6B95-D3(BJ),[183, 205] M06,[143] and B3LYP all give MUEs of around 2 kcal/mol for the same dataset. Taken together, the results in the Supplementary Data 3, 6, and 7 show that significant improvements in universality were indeed obtained by using more diverse training data and supervised learning.

Supplementary Table 8. MUEs (kcal/mol) of subdatabases of the MGCDB84 database.

	NCED[a]	NCEC[b]	NCD[c]	EIE[d]	DIE[e]	TCE[f]	TCD[g]	BH[h]	Overall[i]
ωB97M-V	**0.10**	**0.38**	**0.54**	**0.20**	**1.14**	**1.75**	**3.39**	**1.24**	**0.71**
CF22D	**0.22**	**0.85**	**0.50**	**0.25**	**1.44**	**1.74**	**3.36**	**1.37**	**0.80**
ωB97X-V	**0.13**	**0.47**	**0.64**	**0.20**	**1.49**	2.64	**4.06**	2.02	**1.00**
ωB97X-D	0.23	**0.73**	1.03	0.49	**1.45**	2.66	4.07	1.95	**1.08**
M06-2X-D3(0)	**0.22**	2.05	0.65	0.35	2.08	**2.39**	5.67	1.78	**1.14**
M06-2X	0.30	1.76	0.67	0.34	2.07	**2.41**	5.69	1.77	1.16
MN15	0.32	1.07	**0.58**	0.54	**1.73**	2.62	5.01	**1.46**	1.18
MN15-D3(BJ)	0.32	1.08	**0.58**	0.54	1.75	2.62	5.36	**1.46**	1.20
M08-HX	0.40	1.28	0.72	0.43	1.92	2.59	5.92	**1.44**	1.22
M11-D3(BJ)	0.29	1.17	0.79	0.62	2.32	2.67	6.08	2.00	1.27
M06-D3(0)	0.31	1.33	1.01	0.63	3.05	2.97	4.24	3.41	1.33
B3LYP-D3(BJ)	**0.19**	1.93	1.05	0.36	3.89	2.89	**3.79**	4.81	1.37
M05-2X-D3(0)	0.26	2.60	0.89	**0.29**	2.32	2.86	6.70	2.23	1.37
M11	0.48	1.84	0.94	0.54	2.35	2.74	6.22	2.04	1.39
M05-2X	0.44	1.75	0.91	**0.30**	2.30	2.92	6.73	2.17	1.41
M06	0.54	1.87	1.03	0.48	3.07	3.05	4.36	3.46	1.44
PW6B95-D3(BJ)	0.23	1.89	0.93	0.39	2.07	**2.47**	**4.04**	2.66	1.49
PBE0-D3(BJ)	0.27	2.90	1.37	0.42	2.26	3.76	4.49	3.98	1.66
M06-L-D3(0)	0.28	**0.98**	1.09	0.53	4.30	4.24	6.24	4.86	1.74
M06-L	0.43	1.47	1.10	0.51	4.29	4.27	6.27	4.88	1.82
TPSS-D3(BJ)	0.25	1.45	1.85	0.48	2.96	4.62	7.13	7.72	1.99
PBE0	1.25	2.57	1.18	0.76	2.31	3.77	4.66	3.20	2.02
MN15-L	0.93	7.74	1.04	1.17	3.39	3.38	4.39	3.11	2.04
B3LYP	2.02	6.25	1.24	1.02	4.50	3.75	5.63	4.81	2.61
TPSS	1.80	5.96	1.58	0.92	3.63	4.68	7.62	6.94	2.84
PBE-D3(BJ)	0.28	3.79	2.25	0.52	2.63	7.01	10.43	8.51	2.90
PBE	1.29	2.90	1.87	0.79	3.03	6.93	10.55	7.75	3.22
Average	0.51	2.22	1.04	0.52	2.58	3.35	5.63	3.45	1.61

Notes on Supplementary Table 8. The NCED denotes the noncovalent 'easy' dimers, which consist of 3B-69-DIM, A21x12, A24, AlkBind12, BzDC215, CO2Nitrogen16, DS14, HB15, HB49, HSG, HW30, Ionic43, NBC10, NC15, S22, S66, S66x8, X40; The NCEC denotes the noncovalent 'easy' clusters, which consist of 3B-69-TRIM, CE20, FmH2O10, H2O20Bind10, H2O20Bind4, H2O6Bind8, HW6Cl, HW6F, Shields38, SW49Bind345, SW49Bind6, WATER27; The NCD denotes the noncovalent 'difficult', which consist of Bauza30, CT20, TA13, XB18, XB51; The EIE denotes the isomerization energies 'easy', which consist of ACONF,

AlkIsomer11, Butanediol65, CYCONF, H2O16Rel5, H2O20Rel10, H2O20Rel4, Melatonin52, Pentane14, SW49Rel345, SW49Rel6, YMPJ519; The DIE denotes the isomerization energies 'difficult', which consist of C20C24, DIE60, EIE22, ISOMERIZATION20, Styrene45; The TCD denotes the thermo-chemistry 'difficult', which consist of AlkAtom19, AlkIsod14, BDE99nonMR, BH76RC, BSR36, EA13, G21EA, G21IP, HAT707nonMR, HNBrBDE18, IP13, NBPRC, SN13, TAE140nonMR, WCPT6; The TCE denotes the thermo-chemistry 'easy', which consist of BDE99MR, HAT707MR, PlatonicHD6, PlatonicID6, PlatonicIG6, PlatonicTAE6, TAE140MR; The BH denotes the barrier heights, which consist of BHPERI26, CR20, CRBH20, DBH24, HTBH38, NHTBH38, PX13, WCPT27. The overall MUE includes the NCED, NCEC, NCD, EIE, DIE, TCE, TCD and BH, and AE18 and RG10 subdatabases.

2.5 Results for the DDB22 database

We compare the performance of 25 functionals on the DDB22 database (see Tables 1 and 2). Since the DDB22 database is based on Minnesota database 2019, MGCDB84. and GMTKN55, CUAGAU42, and TMC34. We do not have results for ωB97M-V and ωB97X-V for Minnesota database 2019, so the performance of ωB97M-V and ωB97X-V is not assessed in this part of our study.

The DDB22 database includes four subdatabases: ground state energies (GSE6075, in kcal/mol), excitation energies (EE157, in eV), molecular structures (MS261, in Å), and dipole moments (DM79, in Debye). In our discussion, the performance of selected functionals is assessed on these four subdatabases.

For assessing the performance on the GSE6075 subdatabase, 25 functionals were selected for discussion (see Tables 1 and 2). For the PEC datasets, which are from MGCDB84, the performance of 27 functionals (see Tables 1 and 2) is compared, the same functionals as for MGCDB84 (Supplementary Table 15). For the discussion of EE157, DM79, and MS261 which are from Minnesota Database 2019 (Supplementary Tables 16, 17, and 18), the performance of 29 functionals (see Tables 1 and 2) is compared, the same functionals as for Minnesota Database 2019.

The values highlighted in bold in the following tables represent the five lowest MUEs in each column.

2.5.1 Results for the GSE6075 in the DDB22 database

Of the 6075 ground state energies in DDB22, 2866 were used for training, and 3209 were used only for testing.

GSE6075 is the main part of DDB22, and the performance on this subdatabase is analyzed in three ways:

1. based on the chemical properties: barrier heights (BH), noncovalent interactions (NC), isomerization energies (IE), and thermochemistry properties (TC);

2. based on convergence difficulty of the systems in *Gaussian16* calculations for both complex systems and simple systems;

3. based on the groups of the periodic table: systems containing transition metals (groups 3–12) and systems with only main-group elements (groups 1, 2, and 13-18).

In Supplementary Tables 10, 11, and 14, functionals are ordered by the MUEs on the full GSE6075 in Supplementary Table 9.

Supplementary Table 9. MUEs (kcal/mol) for the GSE6075 subdatabase of the DDB22 database

	GSE_training2866[a]	GSE_test3209[b]	GSE6075[c]
CF22D	**1.34**	**0.75**	**1.03**
MN15	**1.90**	**1.06**	**1.45**
MN15-D3(BJ)	**1.94**	**1.06**	**1.48**
ωB97X-D	**1.85**	1.23	**1.52**
M06-2X-D3(0)	**2.05**	**1.05**	**1.52**
M06-2X	2.07	1.10	1.56
M08-HX	2.11	1.15	1.60
M11-D3(BJ)	2.16	1.20	1.65
PW6B95-D3(BJ)	2.59	**0.88**	1.69
M06-D3(0)	2.15	1.40	1.75
M05-2X-D3(0)	2.44	1.15	1.76
M05-2X	2.35	1.26	1.77
M11	2.28	1.40	1.81
B3LYP-D3(BJ)	2.38	1.31	1.82
M06	2.24	1.54	1.87
PBE0-D3(BJ)	3.04	1.10	2.01
MN15-L	2.99	1.71	2.31
PBE0	2.91	1.83	2.34
M06-L-D3(0)	2.90	2.02	2.44
TPSS-D3(BJ)	3.52	1.49	2.45
M06-L	2.94	2.13	2.51
PBE-D3(BJ)	5.32	1.47	3.29
B3LYP	3.42	3.19	3.30
TPSS	3.95	2.89	3.39
PBE	5.03	2.20	3.53
Average	2.71	1.50	2.07

Notes on Supplementary Table 9. The GSE_training2866 includes NCCE30/18, 2pIsoE4, 4pIsoE4, SR-MGM-BE8, SR-MGN-BE107, MR-MGM-BE4, MR-MGN-BE17, SR-TML-BE11, SR-TMD-BE4, MR-TML-BE12, MR-TMD-BE3, πTC13, HC7/11, IP23, DC9/19, SMAE3/19, CR20, CRBH20, DBH24, WCPT27, DIE60, EIE22, NBPRC, ISOMERIZATION20, AlkIsomer11, CYCONF, H2O16Rel5, H2O20Rel10, H2O20Rel4, Pentane14, SW49Rel345, SW49Rel6, Bauza30, CT20, TA13, CE20, FmH2O10, H2O20Bind10, H2O6Bind8, HW6Cl, HW6F, Shields38, SW49Bind345, SW49Bind6, A21x12, A24, AlkBind12, BzDC215, WCPT6, DS14, HB15, HB49, HW30, NC15, BDE99MR, HAT707MR, PlatonicHD6, PlatonicID6, PlatonicIG6, PlatonicTAE6, AlkAtom19, AlkIsod14, BDE99nonMR, EA13,

HAT707nonMR, HNBrBDE18, SN13, AE18, W4-11, G21EA, G21IP, BH76RC, BSR36, BH76, BHPERI, PX13, WATER27, AHB21, CHB6, IL16, ACONF, BUT14DIOL.

The GSE_test3209 includes TMBH22, ASNC2, ABDE13, WCCR9, YMPJ519, 3B-69-TRIM, XB18, Styrene45, 3B-69-DIM, HSG, NBC10, S66x8, X40, RG10, DIPCS10, PA26, SIE4x4, ALKBDE10, YBDE18, C20C24, AL2X6, HEAVYSB11, ALK8, RC21, G2RC, FH51, TAUT15, DC13, MB16-43, DARC, RSE43, ISOL24, C60ISO, PArel, BHDIV10, INV24, BHROT27, RG18, ADIM6, S22, S66, HEAVY28, CO2Nitrogen16, CARBHB12, PNICO23, WCPT18, HAL59, CDIE20, ISO34, IDISP, ICONF, PCONF21, MCONF, SCONF, UPU23, CUAGAU42, TMD10, MOR13, TMB11.

The GSE6075 includes the GSE_training2866 and GSE_test3209.

Supplementary Table 10. MUEs (kcal/mol) for GSE6075 classified by four chemical properties: barrier heights (BH), noncovalent interactions (NC), isomerization energies (IE), and thermochemistry properties (TC)

	BH318	BH_training206	BH_test112	NC2805	NC_training936	NC_test1869	IE1119	IE_training293	IE_test826	TC1833	TC_training1431	TC_test402
CF22D	**1.31**	**1.41**	**1.14**	**0.27**	**0.35**	**0.22**	**0.54**	**0.39**	**0.60**	**2.44**	**2.17**	**3.40**
MN15	**1.52**	**1.46**	1.63	**0.35**	**0.50**	0.27	0.86	0.57	**0.96**	**3.50**	**3.15**	**4.75**
MN15-D3(BJ)	**1.52**	**1.46**	1.63	**0.35**	**0.50**	0.27	0.86	0.57	**0.96**	**3.57**	3.23	**4.76**
ωB97X-D	**1.69**	**1.82**	**1.43**	**0.29**	**0.40**	0.23	**0.79**	0.56	**0.88**	3.82	**3.06**	6.53
M06-2X-D3(0)	1.92	2.15	1.50	0.38	0.77	**0.19**	0.86	**0.43**	1.02	**3.60**	**3.19**	5.03
M06-2X	1.90	2.15	**1.45**	0.40	0.61	0.29	0.85	**0.45**	1.00	**3.70**	3.34	4.99
M08-HX	**1.50**	**1.39**	1.70	0.43	**0.50**	0.40	0.89	0.51	1.03	3.85	3.59	**4.76**
M11-D3(BJ)	1.75	1.98	**1.33**	**0.35**	0.56	0.25	1.15	0.60	1.34	3.94	3.55	5.32
PW6B95-D3(BJ)	2.40	3.01	**1.28**	0.39	0.77	**0.19**	**0.80**	0.70	**0.84**	4.10	4.12	**4.04**
M06-D3(0)	2.91	3.68	1.49	0.40	0.59	0.30	1.16	0.86	1.26	3.99	**3.21**	6.80
M05-2X-D3(0)	1.99	2.22	1.56	0.48	1.02	**0.21**	0.83	**0.40**	0.98	4.25	3.83	5.77
M05-2X	1.96	2.18	1.55	0.51	0.68	0.42	**0.82**	**0.40**	0.97	4.26	3.87	5.63
M11	1.96	2.03	1.85	0.55	0.67	0.50	1.09	0.59	1.27	4.16	3.72	5.73
B3LYP-D3(BJ)	3.73	4.94	1.50	0.38	0.78	**0.19**	1.14	0.80	1.25	4.09	3.38	6.62
M06	2.93	3.72	1.47	0.60	0.73	0.54	1.03	0.80	1.12	4.13	3.31	7.07
PBE0-D3(BJ)	3.46	4.16	2.17	0.55	1.15	0.24	**0.82**	0.81	**0.83**	4.73	4.56	5.32
MN15-L	2.43	2.79	1.77	1.41	2.68	0.78	1.74	1.45	1.85	4.01	3.54	5.69
PBE0	2.99	3.47	2.10	1.12	0.95	1.20	1.21	0.95	1.30	4.78	4.51	5.73
M06-L-D3(0)	3.73	4.94	1.49	**0.34**	**0.50**	0.27	1.44	1.21	1.51	6.03	4.53	11.38
TPSS-D3(BJ)	5.94	7.79	2.53	0.43	0.75	0.26	1.17	1.18	1.17	5.71	5.18	7.60
M06-L	3.91	4.95	2.00	0.49	0.65	0.42	1.42	1.23	1.48	6.03	4.50	11.47
PBE-D3(BJ)	6.62	8.45	3.25	0.71	1.56	0.29	1.11	1.19	1.08	7.98	8.17	7.29
B3LYP	3.94	5.10	1.81	2.01	2.25	1.88	1.93	1.08	2.24	6.01	4.43	11.63
TPSS	5.67	7.13	2.98	1.81	2.03	1.70	1.75	1.35	1.90	6.40	5.28	10.41
PBE	6.39	7.79	3.83	1.20	1.10	1.25	1.51	1.27	1.59	7.85	7.97	7.43
Average	3.04	3.69	1.86	0.65	0.92	0.51	1.11	0.81	1.22	4.68	4.14	6.61

Notes on Supplementary Table 10. Functionals are ordered by the MUEs on GSE6075 in Supplementary Table 9. The BH_training206 includes CR20, CRBH20, DBH24, WCPT27, BH76, BHPERI, PX13. The BH_test112 includes TMBH22, BHDIV10, INV24, BHROT27, WCPT18, TMB11.

The NC_training936 includes Bauza30, CT20, TA13, CE20, FmH2O10, H2O20Bind10, H2O6Bind8, HW6Cl, HW6F, Shields38, SW49Bind345, SW49Bind6,

WATER27, NCCE30/18, A21x12, A24, AlkBind12, BzDC215, DS14, HB15, HB49, HW30, NC15, AHB21, CHB6, IL16. The NC_test1869 includes XB18, PNICO23, HAL59, ASNC2, 3B-69-TRIM, 3B-69-DIM, HSG, NBC10, S66x8, X40, RG10, RG18, ADIM6, S22, S66, HEAVY28, CO2Nitrogen16, CARBHB12.
The IE_training293 includes DIE60, EIE22, ISOMERIZATION20, 2pIsoE4, 4pIsoE4, AlkIsomer11, CYCONF, H2O16Rel5, H2O20Rel10, H2O20Rel4, Pentane14, SW49Rel345, SW49Rel6, ACONF, BUT14DIOL. The IE_test826 includes Styrene45, C20C24, YMPJ519, ISOL24, C60ISO, PArel, CDIE20, ISO34, IDISP, ICONF, PCONF21, MCONF, SCONF, UPU23, CUAGAU_IE15.

The TC_training1431 includes SR-MGM-BE8, SR-MGN-BE107, MR-MGM-BE4, MR-MGN-BE17, SR-TML-BE11, SR-TMD-BE4, MR-TML-BE12, MR-TMD-BE3, πTC13, HC7/11, IP23, DC9/19, SMAE3/19, NBPRC, AE18, W4-11, G21EA, G21IP, BH76RC, BSR36, BDE99MR, HAT707MR, PlatonicHD6, PlatonicID6, PlatonicIG6, PlatonicTAE6, WCPT6, AlkAtom19, AlkIsod14, BDE99nonMR, EA13, HAT707nonMR, HNBrBDE18, SN13. The TC_test402 includes ABDE13, WCCR9, DIPCS10, PA26, SIE4x4, ALKBDE10, YBDE18, AL2X6, HEAVYSB11, ALK8, RC21, G2RC, FH51, TAUT15, DC13, MB16-43, DARC, RSE43, CUAGAU_TC27, TMD10, MOR13.

The data in GSE6075 are classified into four types of four chemical properties: barrier heights (BH), noncovalent interactions (NC), isomerization energies (IE), and thermochemistry (TC). Supplementary Table 10 shows that among the 25 functionals compared (see Tables 1 and 2), CF22D has the best performance for all four classes. For the IE1119 subdatabase, CF22D, ωB97X-D, and PW6B95-D3(BJ) give the three best performances, with MUEs of 0.54, 0.79 and 0.80 kcal/mol, respectively. For the TC1833 subdatabase, the lowest MUEs belong to CF22D, MN15, and MN15-D3(BJ) with MUEs of 2.44, 3.50 and 3.57 kcal/mol, respectively. For the BH318 subdatabase, the top functionals are CF22D, M08-HX, MN15, MN15-D3(BJ) and ωB97X-D with the CF22D having an MUE of 1.31 kcal/mol and the other four having MUEs in the range 1.50–1.69 kcal/mol. For the NC2805 category, top-performing functionals are CF22D and ωB97X-D with MUEs of 0.27 and 0.29 kcal/mol, respectively.

We can divide each of the four classes into training and testing subsets, and Supplementary Table 10 shows that CF22D has the best performance for both the training and testing subsets of the IE and TC subdatabases. The split into training and testing data yields 8 subdatabases, and Supplementary Table 10 shows that CF22D has the best performance for 6 of them (BH_test112, NC_training936, IE_training293, IE_test826, TC_training1431, TC_test402 categories), second best for BH_training206 category, and fifth best for NC_test1869 category. The results are particularly interesting for barrier heights; for the BH_training206 set, M08-HX gives the best result (1.39 kcal/mol), and CF22D gives the second-best performance with an MUE of 1.41 kcal/mol; however, for BH_test112, CF22D shows excellent transferability, and gives the best result with an MUE of 1.14 kcal/mol.

The comparisons in Supplementary Tables 9 and 10 show that the prediction accuracy of the CF22D functional is highly transferable to properties that are not in the training set.

Supplementary Table 11. MUEs (kcal/mol) for main-group systems (MG) and systems containing transition metals (TM) of GSE6075 from the DDB22 database

	MG5938	MG_training2836	MG_test3102	TM137	TM_training30	TM_test107
CF22D	**0.97**	**1.28**	**0.68**	**3.61**	6.61	**2.77**
MN15	**1.41**	**1.86**	0.99	**3.48**	5.37	**2.96**
MN15-D3(BJ)	1.43	**1.90**	0.99	**3.48**	**5.28**	**2.97**
ωB97X-D	1.44	**1.77**	1.13	5.15	8.90	4.10
M06-2X-D3(0)	**1.35**	1.93	**0.83**	8.79	12.98	7.61
M06-2X	**1.36**	**1.87**	**0.88**	10.33	20.27	7.54
M08-HX	**1.42**	1.93	**0.95**	9.50	18.69	6.93
M11-D3(BJ)	1.53	2.07	1.03	7.08	10.45	6.13
PW6B95-D3(BJ)	1.64	2.56	**0.80**	**3.89**	6.07	**3.28**
M06-D3(0)	1.69	2.11	1.31	4.45	5.71	4.09
M05-2X-D3(0)	1.64	2.35	1.00	6.84	11.77	5.46
M05-2X	1.66	2.25	1.11	6.83	11.73	5.46
M11	1.66	2.15	1.21	8.51	14.73	6.77
B3LYP-D3(BJ)	1.76	2.35	1.22	4.31	**5.24**	4.05
M06	1.81	2.19	1.45	4.55	6.53	4.00
PBE0-D3(BJ)	1.92	2.93	1.00	5.87	13.29	3.79
MN15-L	2.28	2.97	1.65	**3.77**	**4.85**	**3.47**
PBE0	2.26	2.83	1.73	5.85	10.41	4.57
M06-L-D3(0)	2.40	2.88	1.95	4.20	**4.75**	4.05
TPSS-D3(BJ)	2.40	3.49	1.41	4.47	6.31	3.95
M06-L	2.47	2.93	2.05	4.50	**4.25**	4.57
PBE-D3(BJ)	3.24	5.28	1.38	5.19	9.06	4.10
B3LYP	3.24	3.38	3.11	5.94	6.89	5.67
TPSS	3.36	3.93	2.84	4.63	5.82	4.30
PBE	3.48	4.99	2.11	5.70	8.63	4.88
Average	1.99	2.65	1.39	5.64	8.98	4.70

Notes on Supplementary Table 11. Functionals are ordered by the MUEs on GSE6075 in Supplementary Table 9.

The MG_training2836 includes CR20, CRBH20, DBH24, WCPT27, BH76, BHPERI, PX13, DIE60, EIE22, ISOMERIZATION20, 2pIsoE4, 4pIsoE4, AlkIsomer11, CYCONF, H2O16Rel5, H2O20Rel10, H2O20Rel4, Pentane14, SW49Rel345, SW49Rel6, ACONF, BUT14DIOL, Bauza30, CT20, TA13, CE20, FmH2O10, H2O20Bind10, H2O6Bind8, HW6Cl, HW6F, Shields38, SW49Bind345, SW49Bind6, WATER27, NCCE30/18, A21x12, A24, AlkBind12, BzDC215, DS14, HB15, HB49, HW30, NC15, AHB21, CHB6, IL16, SR-MGM-BE8, SR-MGN-

BE107, MR-MGM-BE4, MR-MGN-BE17, πTC13, HC7/11, IP23, DC9/19, SMAE3/19, NBPRC, AE18, W4-11, G21EA, G21IP, BH76RC, BSR36, BDE99MR, HAT707MR, PlatonicHD6, PlatonicID6, PlatonicIG6, PlatonicTAE6, WCPT6, AlkAtom19, AlkIsod14, BDE99nonMR, EA13, HAT707nonMR, HNBrBDE18, SN13. The MG_test3102 includes BHDIV10, INV24, BHROT27, WCPT18, Styrene45, C20C24, YMPJ519, ISOL24, C60ISO, PArel, CDIE20, ISO34, IDISP, ICONF, PCONF21, MCONF, SCONF, UPU23, XB18, PNICO23, HAL59, ASNC2, 3B-69-TRIM, 3B-69-DIM, HSG, NBC10, S66x8, X40, RG10, RG18, ADIM6, S22, S66, HEAVY28, CO2Nitrogen16, CARBHB12, ABDE13, DIPCS10, PA26, SIE4x4, ALKBDE10, YBDE18, AL2X6, HEAVYSB11, ALK8, RC21, G2RC, FH51, TAUT15, DC13, MB16-43, DARC, RSE43.

The TM_training30 includes SR-TML-BE11, SR-TMD-BE4, MR-TML-BE12, MR-TMD-BE3. The TM_test107 includes TMBH22, WCCR9, CUAGAU42, and TMC34, where TMC34 includes TMD10, MOR13, and TMB11. MUE(TMC34) is calculated based on the previous work[177]. Note that TMC34 is a collection of data from Chan et al.'s work.[177] It is a combination of TMD10, MOR13 and TMB11 that employs 34 data points to represent TMC151, which consists of TMD60, MOR41 and TMB50, respectively. The MUE for TMC34 is calculated by, MUE(TMC34)=(EMUE(TMD10)*60+EMUE(MOR13)*41+EMUE(TMB11)*50)/151, where EMUE denotes the estimated MUE defined in ref. 177. Another classification utilized in the DDB21 database is that we divide TMC34 into two categories: TMD10 and MOR13 belong to the TC category, and TMB11 belongs to the BH category, respectively. The MUE analysis here is calculated according to the actual number of data points. For example,

MUE(TC)=(EMUE(TMD10)*10+EMUE(MOR13)*13)/23;

MUE(BH)=(EMUE(TMB11)*11)/11.

Another classification for GSE6075 is to divide the data into systems containing only main-group elements (MG, 5938 data) and systems containing one or more transition-metal atoms (TM, 137 data). Supplementary Table 11 shows the MUEs of

both training and non-training data for the MG and TM subdatabases.

For MG5938, CF22D gives the best result with an MUE of 0.97 kcal/mol, with the second-fifth best being M06-2X-D3(0), M06-2X, MN15 and M08-HX, with MUEs in the range 1.35-1.42 kcal/mol. For both training and testing MG data, CF22D gives the best results with the MUEs of 1.28 and 0.68 kcal/mol, respectively. The second best MUEs for the training and testing subsets of the main-group data are 1.77 and 0.80 kcal/mol, respectively.

For the 137 TM data, MN15 and MN15-D3(BJ) give the best results with the MUEs of 3.48 kcal/mol, and CF22D gives the third-best result with an MUE of 3.61 kcal/mol. However, CF22D gives the best result for the 107 testing TM data (TM_test107). This is challenging test since the TM_training30 subdatabase includes only bond energies (see Notes in Supplementary Table 11), whereas the TM_test107 subdatabase also includes barrier heights. Furthermore, TM_test107 contains a variety of metals (Cu, Ag, Au, Ti, V, Cr, Mn, Fe, Co, Zn, W, Mo, Zr, Re, Ir, and Pd). The MUE of CF22D for the 107 TM testing data is 2.77 kcal/mol, followed by MN15 and MN15-D3(BJ) with MUEs of 2.96 and 2.97 kcal/mol, respectively.

The TM_test107 subdatabase in Supplementary Table 11 includes two barrier-height datasets, namely, TMBH22 and WCCR9. For the TMBH22[113-115] dataset, which describes 22 barrier heights for transition metal reactions, including Mo, W, Re, and Zr, CF22D gives the third best result with an MUE of 1.41 kcal/mol, and it improves significantly as compared to the MN15 and MN15-D3(BJ) functionals, the MUEs of which are both 1.92 kcal/mol. For dataset WCCR9, which includes large cationic transition metal complexes[111, 112], M06-SX is the best performing functional with an MUE of 4.07 kcal/mol (see Supplementary Table 12). CF22D gives an MUE of 6.19 kcal/mol, and it has almost equivalent performance for WCCR9 as MN15 and MN15-D3(BJ), which have MUEs of 6.07 and 6.23 kcal/mol, respectively.

Supplementary Table 12. MUEs (kcal/mol) for four transition metal test sets (functionals are ordered by the MUEs on TM_test107)

	TMBH22	WCCR9	CUAGAU42	TMC34
CF22D	**1.41**	6.19	**1.99**	**3.71**
MN15	1.92	6.07	**2.54**	**3.32**
MN15-D3(BJ)	1.92	6.23	**2.54**	**3.32**
PW6B95-D3(BJ)	**1.44**	5.32	**3.14**	**4.11**
MN15-L	1.93	**4.76**	3.41	**4.19**
revM06	1.98	**4.37**	3.28	4.48
M06-SX	1.65	**4.07**	4.31	4.27
PBE0-D3(BJ)	2.27	6.58	3.54	4.36
M06	**1.25**	**4.49**	3.62	6.11
B3LYP-D3(BJ)	**1.43**	7.19	3.67	5.39
M06-D3(0)	**1.38**	6.43	3.59	5.85
ωB97X-D	2.28	7.18	3.97	4.61
revM06-L	2.45	5.24	4.57	4.86
TPSS	2.77	7.37	**2.63**	6.55
M06-L	2.61	5.23	4.84	5.32
PBE0	2.29	6.15	4.17	6.13
M06-L-D3(0)	2.76	6.11	4.82	5.18
TPSS-D3(BJ)	3.33	8.46	3.34	6.06
PBE	3.48	7.12	3.18	7.28
PBE-D3(BJ)	3.87	7.24	3.63	6.51
M05-2X	2.18	4.84	5.38	7.84
M05-2X-D3(0)	2.20	6.22	5.35	7.50
B3LYP	3.15	7.50	5.25	7.34
M11-D3(BJ)	2.73	5.57	10.12	5.33
M11	2.68	4.81	10.49	5.32
M08-HX	3.15	**4.23**	10.58	5.56
M06-2X	2.48	5.19	11.13	7.00
M06-2X-D3(0)	2.69	5.91	11.11	6.93
Average	2.35	5.93	5.01	5.52

Notes on Supplementary Table 12. For TMBH22 and WCCR9 datasets, results of CF22D, B3LYP-D3(BJ), M05-2X, M05-2X-D3(0), M06-D3(0), M06-2X-D3(0), M06-L-D3(0), M11-D3(BJ), MN15-D3(BJ), PBE-D3(BJ), PBE0-D3(BJ) and TPSS-D3(BJ) are newly calculated from this study, while the results of the remaining functionals are obtained from ref. 199. There are no ωB97M-V and ωB97X-V results for TMBH22 and WCCR9, and thus the functionals listed above do not include ωB97M-V and ωB97X-V functionals. For CUAGAU42 and TMC34 datasets, the results of all functionals listed above are calculated from this study. The results of

ωB97M-V and ωB97X-V for CUAGAU42 and TMC34, obtained from refs. 177, 178, are listed in the main text (Fig. 4). TMC34 is composed of TMD10, MOR13 and TMB11. Among them, the TMB11 and TMBH22 datasets have 5 reactions in common. Since the number of overlapping reactions is small and all the overlaps belong to the test set, we keep both in the DDB22 database.

Supplementary Table 13. MUEs (kcal/mol) of ωB97M-V, ωB97X-V, and some doubly-hybrid functionals for transition metal test sets of CUAGAU42. All the result for CUAGAU42 dataset listed below are from the ref. 178.

MUE	CUAGAU42
Doubly hybrid	
B2PLYP-D3(BJ)	1.46
DSD-PBEP86	1.74
B2GPPLYP-D3(BJ)	1.98
Hybrid	
ωB97M-V	4.92
ωB97X-V	5.62

Note on Supplementary Table 13. B2PLYP-D3(BJ) is the best-performing functional for the CUAGAU42 dataset, with an MUE of 1.46 kcal/mol, followed by DSD-PBEP86 and B2GPPLYP-D3(BJ) with MUEs of 1.74 and 1.98 kcal/mol, respectively. These are all doubly hybrid functionals. The single hybrid CF22D has an MUE of 1.99 kcal/mol for the CUAGAU42 dataset; this is much better than the ωB97M-V and ωB97X-V functionals, which have MUEs of 4.92 and 5.62 kcal/mol, respectively.

Performance on the CUAGUA42 and TMC34 Databases. Two databases incorporated in DDB22 that are not in GMTKN55, MDB2019, or MGCDB84 are CUAGAU42 and TMC34. Results for these databases are given in Fig. 4, Supplementary Tables 12 and 13. Fig. 4 shows the results of 9 representative functionals for the CUAGAU42[178] and TMC34[177] databases from the work of Chan et al.. These datasets were not used for training. CUAGAU42 includes atomization energies, ionization energies, bond dissociation energies, and isomerization energies of small molecular systems containing Cu, Ag, and Au. For CUAGAU42, CF22D gives the best result (MUE of 1.99 kcal/mol), followed by MN15 (2.54 kcal/mol); ωB97M-V ranks seventh among the nine compared functionals in Fig. 4 (4.92 kcal/mol). TMC34 is a cost-effective representation of the TMC151 database consisting of the TMD60[217], MOR41[218] and TMB50[113-115, 219] datasets; it describes diatomic dissociation energies, reaction energies, and barriers for prototypical transition metal reactions. For TMC34, MN15 performs best (MUE = 3.32 kcal/mol),

followed by ωB97X-V and CF22D with MUEs of 3.68 and 3.71 kcal/mol, respectively; ωB97M-V gives an MUE of 3.94 kcal/mol for TMC34, and it is the fourth-ranked functional among the nine compared functionals in Fig. 4.

Combining CUAGAU42 and TMC34 yields a dataset with 76 data for transition-metal chemistry. CF22D is the best-performing functional (2.76 kcal/mol), and MN15 is the second-best (2.89 kcal/mol), followed in order by PW6B95-D3(BJ), ωB97X-D, B3LYP-D3(BJ), and ωB97M-V with MUEs in the range 3.57-4.48 kcal/mol.

Supplementary Table 14. MUEs (kcal/mol) for 'complex' and 'simple' datasets of GSE6075 from the DDB22 database. Functionals are ordered by the MUEs on GSE6075 in Supplementary Table 9

	complex886	complex_training470	complex_test416	simple5189	simple_training2396	simple_test2793
CF22D	**2.84**	**2.62**	**3.09**	**0.72**	**1.09**	**0.40**
MN15	**3.82**	3.43	**4.27**	1.05	**1.60**	0.58
MN15-D3(BJ)	**3.93**	3.63	**4.28**	1.06	1.61	0.58
ωB97X-D	4.50	**3.33**	5.82	**1.01**	**1.56**	0.54
M06-2X-D3(0)	4.75	4.30	5.25	**0.97**	**1.60**	**0.43**
M06-2X	4.97	4.77	5.19	**0.98**	**1.54**	**0.49**
M08-HX	4.95	4.85	5.06	**1.03**	**1.57**	0.57
M11-D3(BJ)	4.96	4.46	5.52	1.09	1.71	0.56
PW6B95-D3(BJ)	**3.32**	**3.19**	**3.46**	1.41	2.48	**0.49**
M06-D3(0)	4.74	**3.22**	6.47	1.24	1.94	0.65
M05-2X-D3(0)	5.33	4.79	5.95	1.15	1.98	**0.44**
M05-2X	5.30	4.81	5.86	1.17	1.87	0.57
M11	5.43	4.84	6.11	1.20	1.78	0.70
B3LYP-D3(BJ)	4.66	**3.15**	6.36	1.33	2.23	0.56
M06	4.90	3.35	6.64	1.35	2.02	0.78
PBE0-D3(BJ)	**4.25**	4.15	**4.36**	1.63	2.82	0.61
MN15-L	**4.25**	3.37	5.24	1.98	2.91	1.18
PBE0	4.50	3.98	5.08	1.97	2.70	1.34
M06-L-D3(0)	7.37	4.67	10.42	1.60	2.56	0.77
TPSS-D3(BJ)	5.70	5.17	6.30	1.89	3.19	0.78
M06-L	7.40	4.55	10.62	1.68	2.63	0.87
PBE-D3(BJ)	6.79	7.38	6.13	2.69	4.92	0.78
B3LYP	7.54	4.27	11.24	2.58	3.25	1.99
TPSS	7.21	5.29	9.39	2.73	3.69	1.92
PBE	6.82	6.97	6.65	2.97	4.65	1.54
Average	5.21	4.34	6.19	1.54	2.39	0.80

Notes on Supplementary Table 14. The 'complex886' includes multireference systems, systems that describe transition-metal chemistry, and some datasets for which the self-consistent-field calculations are hard to converge in Gaussian 16 calculations. In particular, it includes the following datasets: MR-MGM-BE4, MR-MGN-BE17, SR-TML-BE11, SR-TMD-BE4, MR-TML-BE12, MR-TMD-BE3, DC9/19, SMAE3/19 and TMBH22, WCCR9 datasets from Minnesota database 2019, HAT707MR, TAE140MR, BDE99MR, DIE60, EIE22, ISOMERIZATION20, Bauza30, CT20, TA13, PlatonicHD6, PlatonicID6, PlatonicIG6, PlatonicTAE6,

C20C24, Styrene45, XB18 in the MGCDB84 database, SIE4x4, DC13, MB16-43, CDIE20, ISOL24, C60ISO, PNICO23, HAL59, PCONF21, UPU23 in the GMTKN55 database, and the CUAGAU42 and TMC34 datasets that describe transition metals properties. The 'simple5189' subdatabase denotes the collection all the other datasets of GSE6075. The 'complex_training470' includes the MR-MGM-BE4, MR-MGN-BE17, SR-TML-BE11, SR-TMD-BE4, MR-TML-BE12, MR-TMD-BE3, DC9/19, SMAE3/19 from Minnesota database 2019 and HAT707MR, TAE140MR, BDE99MR, DIE60, EIE22, ISOMERIZATION20, Bauza30, CT20, TA13, PlatonicHD6, PlatonicID6, PlatonicIG6, PlatonicTAE6 in the MGCDB84 database. The 'complex_test416' includes TMBH22, WCCR9, C20C24, Styrene45, XB18, SIE4x4, DC13, MB16-43, CDIE20, ISOL24, C60ISO, PNICO23, HAL59, PCONF21, UPU23, CUAGAU42 and TMC34. The 'simple_training2396' includes all datasets in GSE_training2866 except the ones in 'complex_training470'. The 'simple_test2793' includes all datasets in GSE_test3209 except the ones in 'complex_test416'.

This table considers the division of the GSE6075 data into 'complex' and 'simple' subdatabases. The 'complex' cases include multireference systems, systems involving transition metals, and some datasets for which the self-consistent-field calculations are hard to converge in *Gaussian 16* calculations. The rest of the systems in GSE6075 are classified as 'simple' systems. Footnotes to the table give details of the classification.

There are 886 data included in the complex category, containing 470 training data and 416 non-training data. Supplementary Table 14 shows that CF22D gives the best results for both the complex886 subdatabase and the simple5189 subdatabase with MUEs of 2.84 and 0.72 kcal/mol, respectively. For the complex_training470 subdatabase, CF22D is the best performing functional with an MUE of 2.62 kcal/mol, and among the 25 compared functionals (see Tables 1 and 2), CF22D is the only functional whose MUE is lower than 3.15 kcal/mol. CF22D also gives the best result for the complex_test416 subdatabase with an MUE of 3.09 kcal/mol; the next two functionals in this case are PW6B95-D3(BJ) and MN15 with MUEs of 3.46 and 4.27 kcal/mol, respectively.

C_8H_8 isomerization energy. The complex_test416 contains some particularly challenging datasets such as DC13 from the GMTKN55 database,[37, 134-144] including the non-training C_8H_8 isomerization reaction. The isomerization energy of C_8H_8 (from Karton and Martin's study,[141] which is part of the DC13 dataset of the GMTKN55 database[133]) is an example of a particularly difficult case that was not used for training. Supplementary Figure 2 shows the reaction; CF22D gives the best prediction, with only 0.18 kcal/mol deviation from the reference value. PW6B95-D3(BJ) is the second best-performing functional shown in Supplementary Figure 2, with an MUE of 1.50 kcal/mol, and M06-2X is the third-best functional for this reaction with an MUE of 2.67 kcal/mol. This example clearly demonstrates CF22D's ability to treat difficult systems.

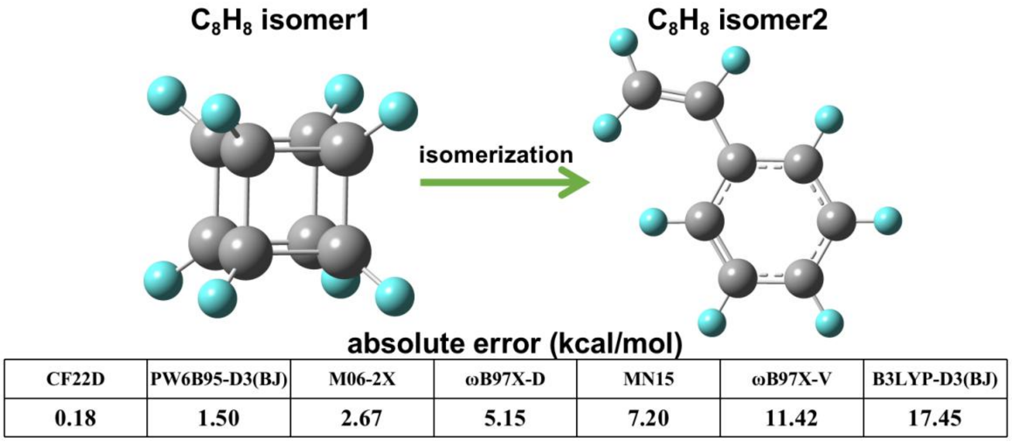

absolute error (kcal/mol)

CF22D	PW6B95-D3(BJ)	M06-2X	ωB97X-D	MN15	ωB97X-V	B3LYP-D3(BJ)
0.18	1.50	2.67	5.15	7.20	11.42	17.45

Supplementary Figure 2. The absolute error (kcal/mol) for the isomerization energy of C_8H_8 from the complex DC13 test set of the GMTKN55 database.[133, 141] For the C_8H_8 isomerization energy in DC13 from the GMTKN55 database, CF22D and ωB97X-D results were calculated from this study. Results of M06-2X, ωB97X-V, MN15, PW6B95-D3(BJ) and B3LYP-D3(BJ) were obtained from ref. 133. The single-point reaction energy of ωB97M-V is not given in ref. 133, and thus only the result of ωB97X-V is included for this comparison.

Supplementary Table 15. MUEs (kcal/mol) for potential energy curves (PECs) in GSE6075 from the DDB22 database.

	A21x12	BzDC215	NBC10	S66x8	RG10	PECs
ωB97M-V	**0.026**	**0.136**	**0.101**	**0.073**	0.045	**0.068**
ωB97X-V	**0.025**	**0.149**	**0.182**	**0.124**	**0.038**	**0.091**
B3LYP-D3(BJ)	**0.046**	**0.140**	0.192	**0.182**	**0.042**	**0.113**
CF22D	**0.052**	**0.161**	0.368	**0.199**	**0.039**	**0.139**
M06-2X-D3(0)	0.076	0.272	0.187	**0.204**	0.070	**0.149**
ωB97X-D	**0.054**	0.223	**0.171**	0.209	0.101	**0.149**
PW6B95-D3(BJ)	0.072	0.265	0.225	0.218	0.061	0.152
TPSS-D3(BJ)	0.082	0.218	0.255	0.241	0.058	0.157
PBE0-D3(BJ)	0.099	0.279	**0.147**	0.271	0.051	0.162
PBE-D3(BJ)	0.131	0.224	**0.145**	0.284	0.061	0.167
M05-2X-D3(0)	0.090	0.339	0.224	0.254	0.054	0.172
M06-L-D3(0)	0.107	**0.193**	0.206	0.287	0.076	0.173
M11-D3(BJ)	0.090	0.334	0.191	0.272	0.063	0.177
M06-2X	0.064	0.221	0.439	0.298	0.069	0.195
MN15-D3(BJ)	0.088	0.421	0.270	0.350	**0.043**	0.213
MN15	0.089	0.417	0.276	0.349	0.044	0.213
M06-D3(0)	0.107	0.226	0.371	0.369	0.101	0.227
M08-HX	0.093	0.350	0.554	0.428	0.072	0.267
M06-L	0.147	0.273	0.508	0.443	0.079	0.268
M11	0.114	0.209	0.729	0.473	0.071	0.285
M05-2X	0.082	0.245	0.889	0.450	0.062	0.292
M06	0.122	0.357	0.737	0.591	0.096	0.349
MN15-L	0.168	0.613	0.940	1.144	**0.043**	0.558
PBE0	0.144	0.810	2.367	1.498	0.070	0.845
PBE	0.126	0.892	2.350	1.557	0.064	0.866
TPSS	0.248	1.248	2.968	2.172	0.095	1.189
B3LYP	0.337	1.610	3.498	2.388	0.130	1.378
Average	0.107	0.401	0.722	0.568	0.067	0.334

Note on Supplementary Table 15. Results of CF22D, MN15-D3(BJ) are newly calculated from this work. Results of the remaining functionals are obtained from the ref. 179. There are 1748 data from potential energy curves in DDB22 (from MGCDB84), including training datasets A21x12[52] (252 data) and BzDC215[56] (215 data), and testing datasets NBC10[50, 104-106] (184 data), S66x8[158] (528 data), and RG10[51] (569 data). Because these datasets are all from the MGCDB84 database, the 27 functionals whose performance is compared for these data are the same as those used in the comparisons for the MGCDB84 database (see Tables 1 and 2).

Supplementary Table 15 shows the MUEs of potential-energy-curve data for these 27 functionals. The table shows that ωB97M-V, ωB97X-V, and B3LYP-D3(BJ) are the three best-performing functionals for potential energy curves with MUEs of 0.068, 0.091, and 0.113 kcal/mol, respectively. CF22D gives the fourth-best performance for potential energy curves with an MUE of 0.139 kcal/mol, which improves significantly from MN15 and MN15-D3(BJ), which both have MUEs of 0.213 kcal/mol and also improves on the average of 0.334 kcal/mol for the 27 compared functionals. Among the 27 compared functionals, the 13 best-performing functionals for potential energy curves are all dispersion-corrected functionals (corrected by including either a nonlocal correlation term or a molecular mechanics term). M06-2X is the best-performing functional (for potential energy curves) that does not have dispersion corrections, and it gives an MUE smaller than two dispersion-corrected functionals, namely, MN15-D3(BJ) and M06-D3(0).

A deficiency of many DFAs that is important for some applications is that they do not contain long-range dispersion effects. Since the dispersion interaction is defined as a correlation-driven interaction between charge distributions with negligible overlap, an approximate density functional with only local correlation terms does not contain long-range dispersion interactions.[220] Adding a molecular mechanics term to correct this can help, but it can yield mixed results for entire potential curves at either long distance or short distance and if the damped-dispersion term is not optimized simultaneously with the density functional term. This shows up better in Fig. 5 than in Supplementary Table 15. The right panel of Fig. 5 shows calculated potential energy curves of benzene-SiH_4. Near the equilibrium position, the binding energies predicted by CF22D for the benzene-SiH_4 complex are very accurate and agree well with the reference values, with deviation from the reference binding energy of less than 0.01 kcal/mol; ωB97M-V, which is the best-performing functional overall for the potential energy curves dataset, has an error of 0.13 kcal/mol in the binding energy, and MN15-D3(BJ) shows a substantially overestimated binding energy. In both cases CF22D predicts accurate long-range tails, whereas the interaction energies calculated by MN15-D3(BJ) quickly decay to nearly zero at long

range, even though it has a damped dispersion term. This illustrates the conclusion that the two terms in the energy expression should optimized simultaneously so the sum is accurate. B3LYP-D3(BJ) provides a reliable long-range van der Waals tail, but at the equilibrium position, it overestimates the benzene-SiH_4 binding energy by about 0.21 kcal/mol. Overall, CF22D provides reliable prediction on the noncovalent interactions not only for the binding energies near the equilibrium distance, but also for the weak interactions at long distance.

2.5.2 Results for the EE157 subdatabase in the DDB22 database

Supplementary Table 16. MUEs (eV) for excitation energies with functionals sorted in order of increasing MUE on EE128.

Functional	EEA11	AEE15	EEAroT5	EE69	EER5	LRCTEE11	EE23	EE139	EE128	EE157
revM06	**0.49**	**0.30**	0.89	**0.29**	0.28	3.60	**0.27**	0.58	**0.32**	**0.67**
MN15	0.55	0.35	0.79	**0.26**	**0.22**	3.47	0.31	0.57	**0.32**	0.69
M062X-D3(0)	0.55	0.34	0.53	0.31	**0.15**	2.78	**0.21**	**0.51**	**0.32**	0.72
M06-2X	0.60	0.34	0.53	**0.30**	**0.15**	**2.59**	**0.23**	**0.50**	**0.32**	**0.64**
ωB97X-D	0.54	**0.28**	**0.49**	**0.30**	0.45	**2.10**	0.30	**0.47**	**0.33**	**0.63**
MN15-D3(BJ)	0.55	0.44	0.74	**0.26**	**0.22**	3.85	0.37	0.61	0.34	0.72
CF22D	0.60	0.37	0.67	0.33	**0.19**	3.25	**0.26**	0.58	0.35	**0.63**
revM11	0.50	**0.30**	**0.38**	**0.30**	0.71	**0.39**	0.35	**0.35**	0.35	**0.47**
M06-SX	**0.48**	0.33	1.04	0.32	0.46	4.56	**0.29**	0.69	0.36	0.78
M052X-D3(0)	0.55	0.37	0.56	0.37	0.25	2.71	0.40	0.57	0.38	0.79
M052X	0.55	0.37	0.56	0.37	0.25	2.71	0.40	0.57	0.38	0.79
PW6B95-D3(BJ)	**0.49**	0.33	1.13	0.45	0.53	4.75	0.30	0.78	0.44	0.86
M11	0.60	0.35	**0.07**	0.49	0.60	**1.04**	0.34	**0.49**	0.44	0.69
PBE0-D3(BJ)	**0.45**	0.31	1.19	0.45	0.48	4.86	0.41	0.80	0.45	0.92
M11-D3(BJ)	0.60	0.39	**0.14**	0.49	0.56	**1.55**	0.33	0.54	0.45	0.83
M08-HX	0.60	0.41	**0.50**	0.46	**0.14**	3.00	0.61	0.68	0.48	0.74
PBE0	**0.45**	0.32	1.20	0.55	0.48	4.85	0.34	0.84	0.50	0.95
B3LYP	0.52	**0.21**	1.38	0.58	0.55	5.34	0.34	0.90	0.52	0.98
revM06-L	0.64	0.47	0.83	0.52	0.69	6.23	0.43	0.98	0.53	1.06
B3LYP-D3(BJ)	0.52	0.31	1.31	0.58	0.55	5.35	0.52	0.91	0.53	1.00
M06L-D3(0)	0.52	0.35	1.66	0.75	0.82	6.61	0.55	1.13	0.65	1.22
M06-L	0.52	0.36	1.66	0.75	0.82	6.61	0.39	1.13	0.66	1.20
MN15-L	0.63	0.36	1.50	0.84	0.70	6.03	0.35	1.12	0.70	1.09
M06-D3(0)	0.94	0.32	1.21	0.88	0.45	4.92	0.66	1.08	0.75	1.17
M06	0.94	0.32	1.21	0.88	0.45	4.92	0.49	1.08	0.75	1.17
TPSS-D3(BJ)	0.59	0.39	1.69	0.88	0.89	6.90	0.74	1.24	0.76	1.31
TPSS	0.59	**0.26**	1.76	1.03	0.89	6.90	0.43	1.30	0.82	1.33
PBE	0.77	0.38	1.79	0.97	0.95	7.12	0.51	1.33	0.83	1.36
PBE-D3(BJ)	0.77	0.49	1.73	1.00	0.95	7.12	0.87	1.36	0.87	1.42
Average	0.59	0.35	1.00	0.55	0.51	4.35	0.41	0.82	0.51	0.93

Notes on Supplementary Table 16. Results of CF22D, B3LYP-D3(BJ), M05-2X, M05-2X-D3(0), M06-D3(0), M06-2X-D3(0), M06-L-D3(0), M11-D3(BJ), MN15-D3(BJ), PBE-D3(BJ), PBE0-D3(BJ), and TPSS-D3(BJ) are newly calculated from this study. All other results were obtained from ref. 199. The EE139 subdatabase include 11 excitation energies of atoms (EEA11), 15 adiabatic excitation energies of molecules (AEE15), 5 excitation energies of Ar-TCNE complexes (EEAroT5), 69

excitation energies of 11 organic molecules (EE69), 5 excitation energies of retinal and dihydroretinal (EER5), 11 excitation energies of long-range charge transfer complexes (LRCTEE11), and 23 excitation energies of molecules (EE23). The EE128 denotes the EE139 subdatabase excluding the LRCTEE11 dataset. The EE157 denotes the merger of EE139 and EE18 (3dEE8, 4dAEE5, pAEE5).

Good performance for both ground states and excited electronic states is important for the application of density functionals to photochemistry, photocatalysis, and spectroscopy. Supplementary Table 16 shows the performance for excitation energies. The EE18 excitation-energy dataset of AME418 is part of the CF22D training set, and EE139 is a non-training test set; EE139 is a merger of the following datasets: 11 excitation energies of atoms (EEA11[116]), 15 adiabatic excitation energies of molecules (AEE15[117]), 5 excitation energies of Ar-TCNE complexes (EEAroT5[118]), 69 excitation energies of 11 organic molecules (EE69[119, 221]), 5 excitation energies of retinal and dihydroretinal (EER5[120, 121]), 23 diverse excitation energies of molecules (EE23[108, 118, 123-126]), and 11 excitation energies of long-range charge transfer complexes (LRCTEE11[122]). These datasets are from Minnesota Database 2019, and therefore (see Tables 1 and 2), 29 functionals are compared; the full comparison broken down by dataset is given in Supplementary Table 16. The typical error in the excitation energies of long-range charge transfer (LRCTEE11) dataset is much larger than the typical error of other datasets, and averaging these errors with the other errors would overshadow the performance on other kinds of excitations. Therefore, we divided EE139 into LRCTEE11 and the remaining excitation energies, which are grouped as EE128. It is instructive to consider EE128 and LRCTEE11 separately.

For EE128, CF22D gives the seventh-best result among the 29 compared functionals, with an MUE of 0.35 eV. The revM06, MN15, M06-2X-D3(0), M06-2X and ωB97X-D functionals are the five best-performing functionals, with MUEs in the range 0.32 - 0.33 eV. Furthermore, CF22D gives the third- and fourth-best results for EE23 and EER5 with MUEs of 0.26 and 0.19 eV, respectively. In previous work[119, 123], we have labeled an error of 0.36 eV or less for Kohn-Sham excitation energies as “moderately successful”, and by this criterion CF22D is moderately successful for

molecular excitation energies.

For LRCTEE11, revM11 gives the best results with an MUE of 0.39 eV, which is the only MUE for LRCTEE11 that is lower than 1.04 eV. The MUE of CF22D for LRCTEE11 is 3.25 eV, which is lower than the average MUE (4.35 eV) of the 29 selected functionals, but not very good. However, we know that getting long-range charge-transfer excitations correct requires a quite different kind of functional.[195, 222] Nevertheless we believe it is appropriate to include some weight on long-range charge transfer in evaluating a functional's universality, in part because some excitations that are not classed as long-range charge transfer excitations may have a small amount of long-range charge transfer character.

When we add all the excitation energies together (EE139 + EE18), CF22D has an MUE of 0.63 eV (high - like all the others - because of the inclusion of long-range charge transfer), which is tied for second best with ωB97X-D; only revM11 does better among the 29 compared functionals. Considering that revM11 and ωB97X-D were originally designed as functionals for calculating electronic excitation energies and that CF22D was trained mainly on ground-state energies, we conclude that CF22D gives acceptably good performance for electronic excitation energies.

2.5.3 Results for the DM79 dataset in the DDB22 database

Supplementary Table 17. The MUEs (Debye) for the database of 79 dipole moments (DM79) and its two subdatabases

	MR23	SR56	DM79
PBE0-D3(BJ)	**0.32**	**0.14**	**0.20**
TPSS-D3(BJ)	**0.33**	0.16	**0.21**
PW6B95-D3(BJ)	0.38	**0.13**	**0.21**
PBE0	0.36	**0.14**	**0.21**
B3LYP-D3(BJ)	**0.35**	0.16	**0.21**
M06-D3(0)	0.38	**0.15**	0.22
TPSS	0.39	0.16	0.22
M06-L-D3(0)	**0.28**	0.20	0.22
B3LYP	0.39	0.16	0.22
M06	0.43	**0.15**	0.23
CF22D	0.48	**0.14**	0.24
PBE-D3(BJ)	0.38	0.18	0.24
revM06	0.43	0.17	0.24
M06-L	0.36	0.20	0.24
PBE	0.40	0.18	0.24
M06-SX	0.44	0.16	0.25
revM06-L	**0.35**	0.20	0.25
MN15-D3(BJ)	0.46	0.16	0.25
ωB97X-D	0.48	0.17	0.26
MN15-L	0.40	0.21	0.26
MN15	0.51	0.16	0.27
revM11	0.62	0.17	0.30
M05-2X	0.50	0.22	0.30
M05-2X-D3(0)	0.50	0.22	0.30
M06-2X	0.58	0.21	0.32
M06-2X-D3(0)	0.61	0.21	0.32
M08-HX	0.67	0.26	0.37
M11-D3(BJ)	0.67	0.27	0.39
M11	0.68	0.27	0.39
Average	0.45	0.18	0.26

Note on Supplementary Table 17. Results of CF22D, B3LYP-D3(BJ), M05-2X, M05-2X-D3(0), M06-D3(0), M06-2X-D3(0), M06-L-D3(0), M11-D3(BJ), MN15-D3(BJ), PBE-D3(BJ), PBE0-D3(BJ), and TPSS-D3(BJ) are newly calculated from this work. All other results were obtained from the ref. 199. Each molecule was consistently optimized with each density functional; that is, each dipole moment is the one predicted by the tested functional at its own geometry. The molecules were divided into single-reference molecules (in the SR56 subset) and multi-reference molecules (in the MR23 subset) by using the B1[223] diagnostic. See ref. 132 for details.

For neutral systems, dipole moments are the first nonzero moment of the charge distribution, and thus they provide a test of how well density functionals predict the charge distribution. Density functionals are compared for their performance on the DM79[131, 132] dataset (from Minnesota Database 2019) of 79 dipole moments in Supplementary Table 17. Most density functionals do well for dipole moments, and all 29 compared functionals have MUEs in the range of 0.20 - 0.39 Debye; CF22D has an MUE of 0.24 Debye.

2.5.4 Results for the MS261 subdatabase in the DDB22 database

Supplementary Table 18. The MUEs (Å) of molecular structure database MS261 and its datasets. Results of CF22D, B3LYP-D3(BJ), M05-2X, M05-2X-D3(0), M06-D3(0), M06-2X-D3(0), M06-L-D3(0), M11-D3(BJ), MN15-D3(BJ), PBE-D3(BJ), PBE0-D3(BJ), and TPSS-D3(BJ) are newly calculated from this work. All other results were obtained from ref. 199.

	MGBL193	TSG48	TMDBL10	DGL6	DGH4	MS261
MN15	**0.0033**	0.021	0.070	0.005	**0.011**	**0.009**
MN15-D3(BJ)	**0.0034**	0.021	0.070	0.004	**0.011**	**0.009**
revM11	0.0057	**0.019**	0.078	0.009	**0.010**	**0.011**
M11-D3(BJ)	0.0060	**0.020**	0.088	0.007	0.017	**0.012**
ωB97X-D	0.0043	0.030	0.083	0.005	0.023	**0.012**
CF22D	0.0075	0.024	0.069	0.007	**0.010**	0.013
M11	0.0060	**0.020**	0.104	0.007	0.018	0.013
M08-HX	0.0060	**0.014**	0.131	0.005	0.047	0.013
M06-2X	0.0040	**0.017**	0.163	**0.004**	0.048	0.013
revM06	0.0054	0.028	0.094	0.008	0.018	0.013
PBE0	0.0047	0.036	0.083	**0.003**	0.014	0.014
M06-2X-D3(0)	**0.0040**	**0.017**	0.177	0.004	0.041	0.014
PW6B95-D3(BJ)	0.0050	0.034	0.091	0.007	0.015	0.014
M05-2X-D3(0)	**0.0040**	0.027	0.143	0.006	0.029	0.014
PBE0-D3(BJ)	0.0047	0.036	0.091	**0.003**	0.014	0.014
M05-2X	0.0040	0.027	0.143	0.006	0.029	0.014
M06-SX	0.0061	0.034	0.086	0.007	0.012	0.014
M06	0.0063	0.037	0.073	0.006	0.022	0.015
M06-D3(0)	0.0063	0.037	0.087	0.006	0.022	0.015
M06-L-D3(0)	0.0044	0.148	**0.058**	**0.002**	0.017	0.033
M06-L	0.0044	0.057	**0.059**	0.006	**0.009**	0.016
PBE-D3(BJ)	0.0102	0.036	**0.042**	0.012	0.018	0.016
MN15-L	0.0098	0.043	0.061	**0.004**	0.024	0.018
revM06-L	0.0076	0.052	0.069	0.009	**0.009**	0.018
B3LYP	**0.0037**	0.065	0.084	0.009	0.028	0.019
B3LYP-D3(BJ)	**0.0036**	0.070	0.095	0.009	0.020	0.020
TPSS-D3(BJ)	0.0077	0.122	**0.057**	0.011	0.015	0.031
TPSS	0.0078	0.122	0.078	0.010	0.014	0.032
PBE	0.0103	0.154	**0.043**	0.013	0.021	0.038
Average	0.0057	0.047	0.089	0.007	0.020	0.017

Note on Supplementary Table 18. The MS261 subdatabase is a merger of a set of 193 bond lengths for 47 organic molecules (MGBL193[129]), a set of 48 geometries of transition-metal compounds (TSG48[130]), a set of 10 transition metal dimer bond lengths (TMDBL10[127]), a set of 6 diatomic bond lengths of light atom molecules

(DGL6[46]), and a set of 4 diatomic bond lengths of molecules containing “heavy” atoms, in particular Zn, Br, and Ag (DGH4[127, 128]). It is a subdatabase in Minnesota Database 2019, and 29 functionals compared for MS261 are listed in Tables 1 and 2; the comparison is in Supplementary Table 18.

Supplementary Table 18 shows that CF22D gives good performance on average for molecular structure prediction. For the whole subdatabase, it is the sixth-best-performing functional with an MUE of 0.013 Å, which is better than the average MUE of 0.017 Å. MN15 and MN15-D3(BJ) are the two best-performing functionals, both with an MUE of 0.009 Å.

In general, the hybrid functionals perform better than local functionals on MS261, although the local functionals show better performance for the TMDBL10 subdatabase. For TMDBL10, the top-performing functionals are PBE-D3(BJ), PBE, TPSS-D3(BJ), M06-L-D3(0) and M06-L; CF22D gives the 7th-best result.

2.6 Results for the basis set superposition errors (BSSEs) and grid errors

Supplementary Table 19. The average basis set superposition errors (BSSEs) of functionals (kcal/mol). The results of MN12-L, MN12-SX, MN15-L, MN15, revM06-L, revM06, and CF22D are calculated in this work, others were taken from ref. 171. The average basis set superposition error of eight non-covalent interaction systems in NCCE31.[224] Basis sets: aDZ is aug-cc-pVDZ; aTZ r is aug-cc-pVTZ; aQZ is aug-cc-pVQZ; a5Z is aug-cc-pV5Z. noCP denotes the binding energies without counterpoise correction.

	average BSSE[b]				
functional	aDZ[c]	aTZ[c]	aQZ[c]	a5Z[c]	BSSE/noCP a5Z[c]
GVWN	0.34	0.10	**0.04**	**0.02**	**0.20%**
B3LYP	0.33	**0.06**	**0.03**	**0.01**	**0.20%**
PBE	0.34	**0.06**	**0.03**	**0.01**	**0.30%**
TPSS	0.35	0.09	**0.02**	**0.01**	**0.40%**
ωB97X	**0.31**	**0.06**	**0.04**	**0.02**	**0.40%**
VSXC	0.36	**0.07**	**0.03**	**0.02**	**0.40%**
M06-2X	**0.32**	0.10	**0.04**	0.04	0.80%
M05-2X	**0.32**	0.11	0.06	0.05	1.00%
CF22D	0.34	0.11	0.06	0.05	1.20%
M05	0.35	0.10	0.07	0.07	1.50%
revM06	**0.30**	0.09	0.09	0.07	1.60%
M08-SO	0.35	0.18	0.14	0.08	1.80%
M08-HX	0.39	0.24	0.13	0.09	2.00%
MN15	0.42	0.23	0.12	0.15	3.20%
M06	0.33	0.19	0.19	0.15	3.60%
M11	0.38	0.34	0.25	0.13	3.60%
revM06-L	**0.28**	**0.08**	0.15	0.15	3.60%
MN15-L	0.34	0.18	0.18	0.15	4.00%
MN12-SX	0.40	0.32	0.24	0.15	4.60%
M11-L	0.43	0.46	0.30	0.18	5.20%
MN12-L	0.44	0.30	0.24	0.19	5.80%
M06-L	**0.30**	0.25	0.34	0.28	6.40%
M06-HF	0.49	0.40	0.35	0.51	12.10%
Average	0.36	0.18	0.14	0.11	2.78%

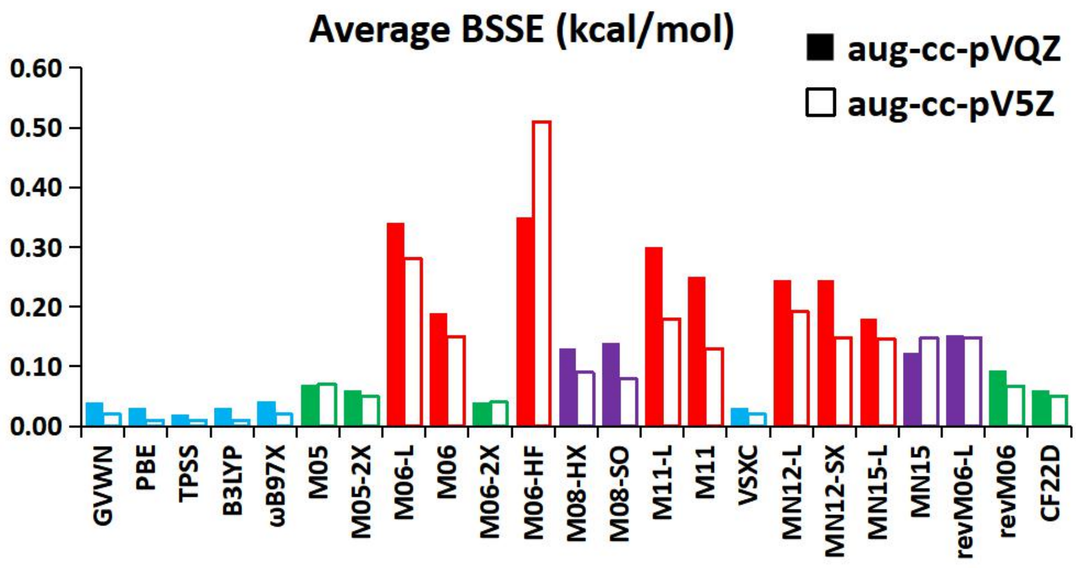

Supplementary Figure 3. The average basis set superposition errors of functionals.

Note on Supplementary Table 19 and Supplementary Figure 3.

Supplementary Table 19 shows the average basis set superposition errors (BSSEs) of functionals for eight noncovalent interaction systems in NCCE31. The results for MN12-L, MN12-SX, MN15-L, MN15, revM06-L, revM06, and CF22D are calculated in this work; the others were taken from ref. 224. Functionals were classified into four categories based on the magnitudes of their BSSEs with the aug-cc-pV5Z (a5Z) basis set, and colors were associated with these classes as follows:

1) subtle, blue (less than 0.5% of binding energies without counterpoise corrected (noCP) on average).

2) mild, green (between 0.5% and 1.5% of noCP on average).

3) moderate, purple (between 1.5% and 5% of noCP on average).

4) severe, red (greater than 5% of noCP on average).

Supplementary Figure 3 shows the average BSSEs with the aug-cc-pVQZ (aQZ) and a5Z basis sets, and it is coded with these colors. The average BSSE of CF22D with aug-cc-pV5Z is 0.05 kcal/mol, which ranks eighth in the 23 compared functionals. The BSSE/noCP of CF22D with a5Z is 1.2%, and thus the severity of its BSSE is mild.

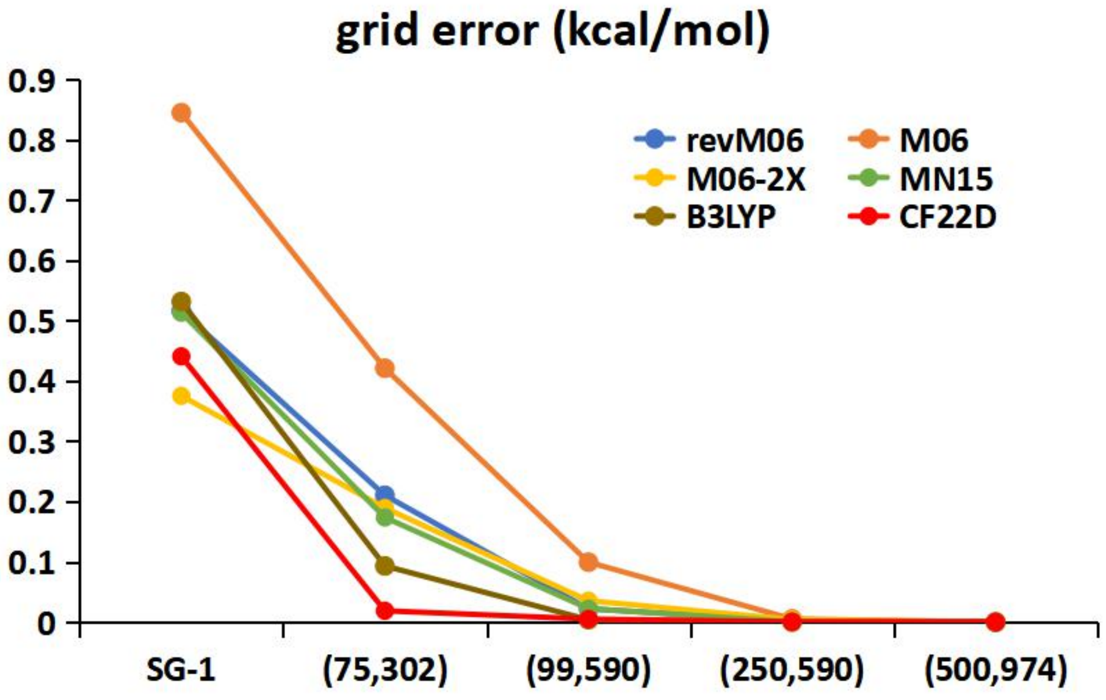

Supplementary Figure 4. The grid errors of functionals for the potential energy curves of Ar, Kr, and Ne dimers. The average root-mean-square deviations (RMSDs) (kcal/mol) of the results calculated with different grids are compared to the results with the (500, 974) grid.

Note on Supplementary Figure 4. It shows the average root-mean-square deviations (RMSDs) (kcal/mol) of the results calculated with different grids with reference to the results with the (500, 974) grid of the potential energy curves of Kr, Ar, and Ne dimers. The results with (75, 302) grid of CF22D are very closely to the results with the (500, 974) grid, and the RMSD is smaller than that of B3LYP. The results of CF22D are converged with the (99, 590) grid which is the default grid setting in the *Gaussian16* program. Therefore, the results of CF22D are not very sensitive to the integration grid.

2.7 Results for the ExL7, ROST61, and CUAGAU-2 datasets

Supplementary Table 20. Deviations of the binding energies for the ExL7 dataset from DFT calculations. Results of CF22D are newly calculated from this work. All other results were obtained from ref. 225. The geometry, basis set and reference energy of the dataset are all taken from refs. 225, 226. The functionals are listed in the ranking of mean percentage absolute deviation (MPAD) results. The definition of MPAD (%) is taken from ref. 225. The ExL7 consists of seven systems. The Systems 1, 2, 5 and 6 are the supramolecular systems. System 3 is a cluster model of ethanol absorbed in ZSM-5. System 4 is an alanine molecule adsorbed in a BN nanotube. System 7 is a DNA double helix. More details on the molecular structures are provided in refs. 225, 226.

Functionals	1	2	3	4	5	6	7	MPAD (%)
CF22D	**4.60**	1.99	**1.91**	**8.94**	**3.95**	**2.32**	10.96	**4.95**
PW6B95-D4-MBD	9.94	**0.36**	3.69	**6.34**	**1.32**	13.10	9.26	**6.29**
PW6B95-D4	9.79	**0.55**	3.58	**6.39**	**1.17**	13.29	9.35	**6.30**
PW6B95-D3(BJ)	**4.61**	2.70	9.00	15.09	**2.12**	**3.64**	8.40	**6.51**
revM11	10.29	**0.95**	**0.68**	**9.20**	7.10	8.06	13.54	**7.12**
M06-L	7.17	**0.00**	**0.03**	10.54	6.83	14.02	11.86	7.21
MN15	**2.88**	1.87	**2.79**	**5.38**	12.19	18.85	17.67	8.80
B97-D3(BJ)	**3.93**	**0.47**	19.15	28.32	**0.22**	9.78	2.63	9.22
B3LYP-D4	16.23	4.83	17.48	14.47	11.79	**3.24**	**0.44**	9.78
B3LYP-D4-MBD	16.72	5.29	17.78	14.64	12.19	**2.89**	**0.70**	10.03
M06-2X-D3(0)	16.25	14.06	7.91	12.84	11.81	6.68	5.73	10.75
ωB97X-D	**5.57**	12.61	12.67	33.71	9.72	**1.24**	**1.03**	10.94
revM06	10.11	6.30	3.20	15.14	11.56	17.61	18.15	11.73
B3LYP-D3(BJ)	13.16	10.14	19.89	21.93	13.18	5.09	**1.74**	12.16
M06-2X	10.22	7.09	**1.45**	17.33	9.89	24.48	14.71	12.17
M11	8.39	3.30	4.30	18.45	13.76	24.89	14.30	12.48
M06-SX	6.58	10.79	6.10	19.69	15.25	22.75	20.06	14.46
M06-L-D4	15.32	22.06	11.14	22.99	15.23	15.28	1.83	14.83
M06-L-D4-MBD	15.40	22.10	11.16	22.99	15.28	15.19	**1.81**	14.85
M06-L-D3(0)	13.60	22.63	10.12	22.04	16.22	19.09	2.34	15.15
PW6B95	12.05	42.17	18.25	54.18	37.33	60.43	28.36	36.11
B3LYP	23.15	73.95	32.20	100.17	56.67	93.78	36.67	59.51
B97	53.05	106.71	44.40	114.08	86.82	129.96	51.58	83.80
Average	12.57	16.21	11.26	25.86	16.16	22.85	12.31	16.75

Note on Supplementary Table 20. Recently Ni et al. presented accurate benchmark datasets for Extra-large molecules (ExL8) with CIM-DLPNO-CCSD(T) results as the reference data.[226] The ExL8 dataset includes eight weakly bonded systems with 200 to 1027 atoms each. The systems include four supramolecular

systems that are dominated by hydrogen-bonding interactions and σ−σ dispersion, ethanol adsorbed in a 76T zeolite cluster, alanine in a boron nitride nanotube, a DNA double helix, and a protein−ligand complex. This provides a means to testing the accuracy of noncovalent interactions for extra-large molecules, and the results can be used to evaluate the accuracies of wave function methods and density functionals. Wu and Truhlar evaluated some density functionals on a truncated version of this benchmark set called ExL7 (they omitted the protein-ligand complex of ExL8). They found that PW6B95-D4, PW6B95-D3(BJ), revM11, M06-L, and MN15 are the five most accurate functionals for ExL7 among the 22 functionals they tested.[225] Our results in Supplementary Table 20 show that CF22D has better performance than any of the functionals in ref. 225, and it has an MPAD of only 4.95%. Among the functionals without molecular mechanics terms, revM11, M06-L, and MN15 stand out with MPADs of 7.12%, 7.21%, and 8.80%, respectively, which are better than some functionals that have molecular mechanics terms, such as B3LYP-D4, ωB97X-D, and B3LYP-D3(BJ) with MPADs of 9.78%, 10.94%, and 12.16%, respectively.

Supplementary Table 21. The MUEs (kcal/mol) for the ROST61 dataset. Results of CF22D are newly calculated in this work. All other results in this table were obtained from ref. 227. The geometry, basis set, and reference energy of the dataset are all taken from ref. 227. The functionals are listed in the ranking order of the MUEs, which are given in kcal/mol. The “Average_1” MUE only takes the results of doubly-hybrid functionals and ordinary functionals with dispersion correction into account. The “Average_2” MUE only takes the results of non-doubly-hybrid functionals into account.

Doubly-hybrid functional			
PWPB95-D4	1.64	revDSD-PBEPBE-D4	2.31
PWPB95-D3	1.85	B2PLYP-D4	2.34
revDSD-PBEP86-D3	1.98	PBE0-DH-D4	2.51
B2GP-PLYP-D3	1.99	PBE0-DH-D3	2.97
revDOD-PBEP86-D3	2.02	PWPB95	3.08
revDSD-BLYP-D3	2.03	revDSD-PBEP86	3.56
revDSD-PBEPBE-D3	2.09	revDOD-PBEP86	3.71
mPW2PLYP-D3	2.19	B2GP-PLYP	3.72
revDSD-PBEP86-D4	2.19	revDSD-PBEPBE	3.83
revDOD-PBEP86-D4	2.23	revDSD-BLYP	4.17
mPW2PLYP-D4	2.29	PBE0-DH	4.27
B2PLYP-D3	2.30	mPW2PLYP	4.36
revDSD-BLYP-D4	2.30	B2PLYP	5.01
B2GP-PLYP-D4	2.31		
Ordinary functional with dispersion correction			
TPSS0-D3	2.28	TPSS-D4	3.72
TPSS0-D4	2.28	M06-D4	3.79
PW6B95-D4	2.54	ωB97X-D3(BJ)	3.83
PW6B95-D3	2.59	M06-L-D4	3.89
TPSSh-D4	2.61	M06-D3	3.90
TPSSh-D3	2.62	M06-L-D3	4.00
PBE0-D4	2.65	B97-3c	4.01
PBE0-D3	2.69	CF22D	4.03
ωB97X-V	2.80	PBE-D4	4.04
ωB97M-V	2.85	SCAN-D3	4.10
r2SCAN-3c	2.89	SCAN-D4	4.10
B3LYP-NL	3.00	revPBE-D4	4.18
B3LYP-D3(no ATM)	3.19	ωB97X-D4	4.18
B3LYP-D3	3.20	BP86-D4	4.19
B3LYP-D4	3.25	PBE-D3	4.22
r2SCAN-D3	3.33	revPBE-D3	4.31
r2SCAN-D4	3.38	BP86-D3	4.62
r2SCAN-VV10	3.40	BLYP-D4	5.11
B97M-V	3.42	B97-D3	5.12
B97M-D3	3.58	BLYP-D3	5.22
revTPSS-D4	3.63	M06-2X-D4	6.33
revTPSS-D3	3.64	M06-2X-D3	6.38
TPSS-D3	3.68		
Ordinary functional without dispersion correction			
MN15	3.99	M06-2X	6.38
M06-L	4.15	TPSS0	6.57
r2SCAN	4.22	TPSSh	6.75
revM06-L	4.37	PBE	7.12
M06	4.41	TPSS	7.51
SCAN	4.49	BP86	8.71
ωB97X	4.93	B3LYP	9.97
PW6B95	5.13	B3LYP/SVP	11.03
PBE0	6.17	revPBE	11.15
revTPSS	6.37	BLYP	11.65
Average_1	3.36	Average_2	4.64

Supplementary Table 22. The MUEs (kcal/mol) for the CUAGAU-2 dataset. Results of CF22D are newly calculated from this work. All other results were obtained from ref. 228 . The geometry, basis set and reference energy of the dataset are all taken from ref. 228. The functionals are listed in the ranking order of MUE (kcal/mol) results for the CUAGAU-2 dataset. The abbreviations for the four subsets of CUAGAU-2 in the table headings are: AE denotes atomization energy; IE denotes ionization energy; ISO denotes isomerization energy; BE denotes binding energy. The average MUE only takes the results of non-doubly-hybrid functionals into account.

(kcal/mol)	AE	IE	ISO	BE	CUAGAU-2
		Doubly-hybrid functionals			
revDSD-PBEP86	2.96	4.13	1.65	1.65	2.27
DSD-PBEP86	5.04	2.99	1.89	1.94	2.68
		Ordinary functionals			
CF22D	**4.09**	**3.23**	**5.83**	4.08	**4.60**
mBEEF	**4.35**	5.11	7.53	**3.01**	**5.23**
TPSS	7.65	**3.68**	6.50	**3.11**	**5.33**
TPSSh	**5.28**	5.40	7.41	**3.15**	**5.45**
MN15	**5.35**	**4.13**	**6.14**	5.64	**5.54**
B97M-rV	7.79	4.37	6.72	3.47	5.64
B97-D3BJ	**6.62**	6.41	6.79	**3.37**	5.69
PBE	7.84	4.59	6.24	4.68	5.86
PW6B95-D2	7.62	4.78	6.62	4.59	5.95
PBE0	10.95	4.95	6.33	4.83	6.60
M06	8.68	4.47	6.74	6.19	6.62
HSE-HJS	11.14	6.09	**6.05**	4.68	6.64
B97-1	9.73	6.48	6.84	4.80	6.76
SCAN0	10.35	5.16	6.86	5.54	6.91
M06-L	10.95	8.68	7.15	**3.30**	6.98
ωB97X	9.75	9.70	**6.21**	4.95	7.05
SCAN	9.70	4.90	8.44	4.85	7.12
ωB97M-V	9.46	11.97	6.45	4.95	7.41
GAM	12.60	**4.21**	7.60	5.45	7.46
MN15-L	7.60	12.07	8.15	4.61	7.58
ωB97X-V	12.55	13.12	6.31	4.80	8.08
MN12-SX	14.05	10.71	**5.50**	6.43	8.20
B3LYP	17.69	**3.11**	7.74	5.76	8.44
B97-3-D2	17.81	9.87	7.96	7.72	10.09
HSEB	19.81	10.47	6.93	8.27	10.35
M11	23.80	21.99	6.52	6.81	12.24
M06-2X	29.09	11.97	7.65	9.63	13.05
Average[b]	10.70	7.20	6.51	4.91	7.29

Note on Supplementary Tables 21 and 22. Maurer et al. presented reactions of open-shell single-reference transition metal complexes (ROST61) benchmark,[227] which includes a diverse collection of open-shell organometallic reactions, and performed coupled-cluster calculations to obtain best-estimate reference reaction energies. This dataset is composed of 61 reactions involving 150 molecules. As shown in Supplementary Table 21, PWPB95-D4 gives the best result with an MUE of 1.64 kcal/mol, and TPSS0-D3 gives the best result among the non-doubly-hybrid functionals, with an MUE of 2.28 kcal/mol. CF22D gives an MUE of 4.03 kcal/mol, which is better than the average MUE of non-doubly-hybrid functionals.

The CUAGAU-2 dataset[228] includes larger clusters and additional ligands as compared to the original CUAGAU set (CUAGAU42)[178]. It is based on 131 species, and it yields 123 data points of independent thermochemical quantities. Compared to CUAGAU42, the CUAGAU-2 set includes clusters as large as M_6, and the ligands that are newly added include O and CO. For the metal systems in CUAGAU-2, the lowest spin states are applied in all cases. The CUAGAU-2 dataset is classified in the same way as was done for the CUAGAU42 dataset: atomization energy (AE), ionization energy (IE), isomerization energy (ISO), and binding energy (BE). These four categories contain 24, 18, 45, and 36 data points, respectively. The CUAGAU-2 systems are very challenging for most of the density functionals. The doubly-hybrid functionals revDSD-PBEP86 and DSD-PBEP86 give the top two performances for the CUAGAU-2 dataset with MUEs of 2.27 and 2.68 kcal/mol, respectively. The MUE of CF22D is 4.60 kcal/mol, which is the best-performing functional among the selected 27 non-doubly-hybrid functionals. CF22D is also among the top five performing functionals in the AE, IE, and ISO categories. The performance of MN15 ranks fifth for CUAGAU-2 among the selected 27 ordinary functionals, with an MUE of 5.54 kcal/mol (see Supplementary Table 22). In contrast, the ωB97M-V and ωB97X-V functionals do not perform well on this dataset, with MUEs of 7.41 and 8.08 kcal/mol, respectively, which are both worse than the average MUE (7.29 kcal/mol) of the 27 ordinary functionals.

3. References

1. Hill, T.L. On Steric Effects. *J. Chem. Phys.* **14**, 465-465 (1946).
2. Westheimer, F.H. & Mayer, J.E. The Theory of the Racemization of Optically Active Derivatives of Diphenyl. *J. Chem. Phys.* **14**, 733-738 (1946).
3. Hendrickson, J.B. Molecular Geometry. I. Machine Computation of the Common Rings. *J. Am. Chem. Soc.* **83**, 4537-4547 (1961).
4. Allinger, N.L., Tribble, M.T., Miller, M.A. & Wertz, D.H. Conformational analysis. LXIX. Improved force field for the calculation of the structures and energies of hydrocarbons. *J. Am. Chem. Soc.* **93**, 1637-1648 (1971).
5. Briggs, J.M., Matsui, T. & Jorgensen, W.L. Monte Carlo simulations of liquid alkyl ethers with the OPLS potential functions. *J. Comp. Chem.* **11** (1990).
6. Smith, J.C. & Karplus, M. Empirical force field study of geometries and conformational transitions of some organic molecules. *J. Am. Chem. Soc.* **114**, 801-812 (1992).
7. Hagler, A.T. & Ewig, C.S. On the use of quantum energy surfaces in the derivation of molecular force fields. *Comput. Phys. Commun.* **84**, 131-155 (1994).
8. Cornell, W.D. et al. A Second Generation Force Field for the Simulation of Proteins, Nucleic Acids, and Organic Molecules. *J. Am. Chem. Soc.* **117**, 5179-5197 (1995).
9. Behler, J. & Parrinello, M. Generalized Neural-Network Representation of High-Dimensional Potential-Energy Surfaces. *Phys. Rev. Lett.* **98**, 146401 (2007).
10. Handley, C.M. & Popelier, P.L.A. Potential Energy Surfaces Fitted by Artificial Neural Networks. *J. Phys. Chem. A* **114**, 3371-3383 (2010).
11. Glielmo, A., Zeni, C. & De Vita, A. Efficient nonparametric n-body force fields from machine learning. *Phys. Rev. B* **97**, 184307 (2018).
12. Zeni, C., Rossi, K., Glielmo, A. & Baletto, F. On machine learning force fields for metallic nanoparticles. *Advances in Physics: X* **4**, 1654919 (2019).
13. Gkeka, P. et al. Machine Learning Force Fields and Coarse-Grained Variables in Molecular Dynamics: Application to Materials and Biological Systems. *J. Chem. Theory Comput.* **16**, 4757-4775 (2020).
14. Unke, O.T., Koner, D., Patra, S., Käser, S. & Meuwly, M. High-dimensional potential energy surfaces for molecular simulations: from empiricism to machine learning. *Mach. Learn.: Sci. Technol.* **1**, 013001 (2020).
15. Unke, O.T. et al. Machine Learning Force Fields. *Chem. Rev.* **121**, 10142-10186 (2021).
16. Kulichenko, M. et al. The Rise of Neural Networks for Materials and Chemical Dynamics. *J. Phys. Chem. Lett.* **12**, 6227-6243 (2021).
17. Poltavsky, I. & Tkatchenko, A. Machine Learning Force Fields: Recent Advances and Remaining Challenges. *J. Phys. Chem. Lett.* **12**, 6551-6564 (2021).
18. Miksch, A.M., Morawietz, T., Kästner, J., Urban, A. & Artrith, N. Strategies for the construction of machine-learning potentials for accurate and efficient atomic-scale simulations. *Mach. Learn.: Sci. Technol.* **2** (2021).
19. Gokcan, H. & Isayev, O. Learning molecular potentials with neural networks. *Wiley Interdiscip. Rev. Comput. Mol. Sci.* **12**, e1564 (2022).
20. Zhang, L., Lin, D.-Y., Wang, H., Car, R. & E, W. Active learning of uniformly accurate interatomic potentials for materials simulation. *Phys. Rev. Mater.* **3**, 023804 (2019).
21. Snyder, J.C., Rupp, M., Hansen, K., Müller, K.-R. & Burke, K. Finding Density Functionals

with Machine Learning. *Phys. Rev. Lett.* **108**, 253002 (2012).

22. Lei, X. & Medford, A.J. Design and analysis of machine learning exchange-correlation functionals via rotationally invariant convolutional descriptors. *Phys. Rev. Mater.* **3**, 063801 (2019).
23. Dick, S. & Fernandez-Serra, M. Machine learning accurate exchange and correlation functionals of the electronic density. *Nat. Commun.* **11**, 3509 (2020).
24. Nagai, R., Akashi, R. & Sugino, O. Completing density functional theory by machine learning hidden messages from molecules. *npj Comput. Mater.* **6**, 43 (2020).
25. Kalita, B., Li, L., McCarty, R.J. & Burke, K. Learning to Approximate Density Functionals. *Acc. Chem. Res.* **54**, 818-826 (2021).
26. Chen, Y., Zhang, L., Wang, H. & E, W. DeePKS: A Comprehensive Data-Driven Approach toward Chemically Accurate Density Functional Theory. *J. Chem. Theory Comput.* **17**, 170-181 (2021).
27. Margraf, J.T. & Reuter, K. Pure non-local machine-learned density functional theory for electron correlation. *Nat. Commun.* **12**, 344 (2021).
28. Kasim, M.F. & Vinko, S.M. Learning the Exchange-Correlation Functional from Nature with Fully Differentiable Density Functional Theory. *Phys. Rev. Lett.* **127**, 126403 (2021).
29. Bystrom, K. & Kozinsky, B. CIDER: An Expressive, Nonlocal Feature Set for Machine Learning Density Functionals with Exact Constraints. *J. Chem. Theory Comput.* **18**, 2180-2192 (2022).
30. Kirkpatrick, J. et al. Pushing the frontiers of density functionals by solving the fractional electron problem. *Science* **374**, 1385-1389 (2021).
31. King, D.S., Truhlar, D.G. & Gagliardi, L. Machine-Learned Energy Functionals for Multiconfigurational Wave Functions. *J. Phys. Chem. Lett.* **12**, 7761-7767 (2021).
32. Gianturco, F.A. et al. Computed and measured transport coefficients for CO-He mixtures: testing a density functional approach. *Mol. Phys.* **94**, 605-622 (1998).
33. Wu, Q. & Yang, W. Empirical correction to density functional theory for van der Waals interactions. *J. Chem. Phys.* **116**, 515-524 (2002).
34. Du, A.J. & Smith, S.C. Van der Waals-corrected density functional theory: benchmarking for hydrogen-nanotube and nanotube-nanotube interactions. *Nanotechnology* **16**, 2118-2123 (2005).
35. Grimme, S., Antony, J., Ehrlich, S. & Krieg, H. A consistent and accurate ab initio parametrization of density functional dispersion correction (DFT-D) for the 94 elements H-Pu. *J. Chem. Phys.* **132**, 154104 (2010).
36. Grimme, S. Density functional theory with London dispersion corrections. *Wiley Interdiscip. Rev. Comput. Mol. Sci.* **1**, 211-228 (2011).
37. Goerigk, L. & Grimme, S. A General Database for Main Group Thermochemistry, Kinetics, and Noncovalent Interactions − Assessment of Common and Reparameterized (meta-)GGA Density Functionals. *J. Chem. Theory Comput.* **6**, 107-126 (2010).
38. Karton, A. & Goerigk, L. Accurate reaction barrier heights of pericyclic reactions: Surprisingly large deviations for the CBS-QB3 composite method and their consequences in DFT benchmark studies. *J. Comput. Chem.* **36**, 622-632 (2015).
39. Yu, L.-J., Sarrami, F., O'Reilly, R.J. & Karton, A. Can DFT and ab initio methods describe all aspects of the potential energy surface of cycloreversion reactions? *Mol. Phys.* **114**, 21-

33 (2016).

40. Yu, L.-J., Sarrami, F., O'Reilly, R.J. & Karton, A. Reaction barrier heights for cycloreversion of heterocyclic rings: An Achilles' heel for DFT and standard ab initio procedures. *Chem. Phys.* **458**, 1-8 (2015).
41. Karton, A., O'Reilly, R.J., Chan, B. & Radom, L. Determination of Barrier Heights for Proton Exchange in Small Water, Ammonia, and Hydrogen Fluoride Clusters with G4(MP2)-Type, MPn, and SCS-MPn Procedures—A Caveat. *J. Chem. Theory Comput.* **8**, 3128-3136 (2012).
42. Chan, B., Gilbert, A.T.B., Gill, P.M.W. & Radom, L. Performance of Density Functional Theory Procedures for the Calculation of Proton-Exchange Barriers: Unusual Behavior of M06-Type Functionals. *J. Chem. Theory Comput.* **10**, 3777-3783 (2014).
43. Karton, A., O'Reilly, R.J. & Radom, L. Assessment of Theoretical Procedures for Calculating Barrier Heights for a Diverse Set of Water-Catalyzed Proton-Transfer Reactions. *J. Phys. Chem. A* **116**, 4211-4221 (2012).
44. Zhao, Y., Lynch, B.J. & Truhlar, D.G. Multi-coefficient extrapolated density functional theory for thermochemistry and thermochemical kinetics. *Phys. Chem. Chem. Phys.* **7**, 43-52 (2005).
45. Zhao, Y., González-García, N. & Truhlar, D.G. Benchmark Database of Barrier Heights for Heavy Atom Transfer, Nucleophilic Substitution, Association, and Unimolecular Reactions and Its Use to Test Theoretical Methods. *J. Phys. Chem. A* **109**, 2012-2018 (2005).
46. Peverati, R. & Truhlar, D.G. Quest for a universal density functional: the accuracy of density functionals across a broad spectrum of databases in chemistry and physics. *Philos. Trans. Royal Soc. A* **372**, 20120476 (2014).
47. Vydrov, O.A. & Van Voorhis, T. Benchmark Assessment of the Accuracy of Several van der Waals Density Functionals. *J. Chem. Theory Comput.* **8**, 1929-1934 (2012).
48. Lange, K.M.d. & Lane, J.R. Explicit correlation and intermolecular interactions: Investigating carbon dioxide complexes with the CCSD(T)-F12 method. *J. Chem. Phys.* **134**, 034301 (2011).
49. McMahon, J.D. & Lane, J.R. Explicit correlation and basis set superposition error: The structure and energy of carbon dioxide dimer. *J. Chem. Phys.* **135**, 154309 (2011).
50. Marshall, M.S., Burns, L.A. & Sherrill, C.D. Basis set convergence of the coupled-cluster correction, $\delta_{MP2}^{CCSD(T)}$: Best practices for benchmarking non-covalent interactions and the attendant revision of the S22, NBC10, HBC6, and HSG databases. *J. Chem. Phys.* **135**, 194102 (2011).
51. Tang, K.T. & Toennies, J.P. The van der Waals potentials between all the rare gas atoms from He to Rn. *J. Chem. Phys.* **118**, 4976-4983 (2003).
52. Witte, J., Goldey, M., Neaton, J.B. & Head-Gordon, M. Beyond Energies: Geometries of Nonbonded Molecular Complexes as Metrics for Assessing Electronic Structure Approaches. *J. Chem. Theory Comput.* **11**, 1481-1492 (2015).
53. Řezáč, J. & Hobza, P. Describing Noncovalent Interactions beyond the Common Approximations: How Accurate Is the "Gold Standard," CCSD(T) at the Complete Basis Set Limit? *J. Chem. Theory Comput.* **9**, 2151-2155 (2013).
54. Bauzá, A., Alkorta, I., Frontera, A. & Elguero, J. On the Reliability of Pure and Hybrid DFT Methods for the Evaluation of Halogen, Chalcogen, and Pnicogen Bonds Involving Anionic and Neutral Electron Donors. *J. Chem. Theory Comput.* **9**, 5201-5210 (2013).

55. Otero-de-la-Roza, A., Johnson, E.R. & DiLabio, G.A. Halogen Bonding from Dispersion-Corrected Density-Functional Theory: The Role of Delocalization Error. *J. Chem. Theory Comput.* **10**, 5436-5447 (2014).
56. Crittenden, D.L. A Systematic CCSD(T) Study of Long-Range and Noncovalent Interactions between Benzene and a Series of First- and Second-Row Hydrides and Rare Gas Atoms. *J. Phys. Chem. A* **113**, 1663-1669 (2009).
57. Steinmann, S.N., Piemontesi, C., Delachat, A. & Corminboeuf, C. Why are the Interaction Energies of Charge-Transfer Complexes Challenging for DFT? *J. Chem. Theory Comput.* **8**, 1629-1640 (2012).
58. Mintz, B.J. & Parks, J.M. Benchmark Interaction Energies for Biologically Relevant Noncovalent Complexes Containing Divalent Sulfur. *J. Phys. Chem. A* **116**, 1086-1092 (2012).
59. Lao, K.U. & Herbert, J.M. Accurate and Efficient Quantum Chemistry Calculations for Noncovalent Interactions in Many-Body Systems: The XSAPT Family of Methods. *J. Phys. Chem. A* **119**, 235-252 (2015).
60. Lao, K.U. & Herbert, J.M. An improved treatment of empirical dispersion and a many-body energy decomposition scheme for the explicit polarization plus symmetry-adapted perturbation theory (XSAPT) method. *J. Chem. Phys.* **139**, 034107 (2013).
61. Bryantsev, V.S., Diallo, M.S., van Duin, A.C.T. & Goddard, W.A. Evaluation of B3LYP, X3LYP, and M06-Class Density Functionals for Predicting the Binding Energies of Neutral, Protonated, and Deprotonated Water Clusters. *J. Chem. Theory Comput.* **5**, 1016-1026 (2009).
62. Řezáč, J. & Hobza, P. Advanced Corrections of Hydrogen Bonding and Dispersion for Semiempirical Quantum Mechanical Methods. *J. Chem. Theory Comput.* **8**, 141-151 (2012).
63. Copeland, K.L. & Tschumper, G.S. Hydrocarbon/Water Interactions: Encouraging Energetics and Structures from DFT but Disconcerting Discrepancies for Hessian Indices. *J. Chem. Theory Comput.* **8**, 1646-1656 (2012).
64. Temelso, B., Archer, K.A. & Shields, G.C. Benchmark Structures and Binding Energies of Small Water Clusters with Anharmonicity Corrections. *J. Phys. Chem. A* **115**, 12034-12046 (2011).
65. Mardirossian, N., Lambrecht, D.S., McCaslin, L., Xantheas, S.S. & Head-Gordon, M. The Performance of Density Functionals for Sulfate–Water Clusters. *J. Chem. Theory Comput.* **9**, 1368-1380 (2013).
66. Tentscher, P.R. & Arey, J.S. Binding in Radical-Solvent Binary Complexes: Benchmark Energies and Performance of Approximate Methods. *J. Chem. Theory Comput.* **9**, 1568-1579 (2013).
67. Granatier, J., Pitoňák, M. & Hobza, P. Accuracy of Several Wave Function and Density Functional Theory Methods for Description of Noncovalent Interaction of Saturated and Unsaturated Hydrocarbon Dimers. *J. Chem. Theory Comput.* **8**, 2282-2292 (2012).
68. Boese, A.D. Assessment of Coupled Cluster Theory and more Approximate Methods for Hydrogen Bonded Systems. *J. Chem. Theory Comput.* **9**, 4403-4413 (2013).
69. Boese, A.D. Basis set limit coupled-cluster studies of hydrogen-bonded systems. *Mol. Phys.* **113**, 1618-1629 (2015).
70. Boese, A.D. Density Functional Theory and Hydrogen Bonds: Are We There Yet? *Chem.*

Phys. Chem. **16**, 978-985 (2015).

71. Lao, K.U., Schäffer, R., Jansen, G. & Herbert, J.M. Accurate Description of Intermolecular Interactions Involving Ions Using Symmetry-Adapted Perturbation Theory. *J. Chem. Theory Comput.* **11**, 2473-2486 (2015).
72. Fanourgakis, G.S., Aprà, E. & Xantheas, S.S. High-level ab initio calculations for the four low-lying families of minima of $(H_2O)_{20}$. I. Estimates of MP2/CBS binding energies and comparison with empirical potentials. *J. Chem. Phys.* **121**, 2655-2663 (2004).
73. Anacker, T. & Friedrich, J. New accurate benchmark energies for large water clusters: DFT is better than expected. *J. Comput. Chem.* **35**, 634-643 (2014).
74. Smith, D.G.A., Jankowski, P., Slawik, M., Witek, H.A. & Patkowski, K. Basis Set Convergence of the Post-CCSD(T) Contribution to Noncovalent Interaction Energies. *J. Chem. Theory Comput.* **10**, 3140-3150 (2014).
75. Schwabe, T. An isomeric reaction benchmark set to test if the performance of state-of-the-art density functionals can be regarded as independent of the external potential. *Phys. Chem. Chem. Phys.* **16**, 14559-14567 (2014).
76. Karton, A., Gruzman, D. & Martin, J.M.L. Benchmark Thermochemistry of the C_nH_{2n+2} Alkane Isomers (n = 2−8) and Performance of DFT and Composite Ab Initio Methods for Dispersion-Driven Isomeric Equilibria. *J. Phys. Chem. A* **113**, 8434-8447 (2009).
77. Kozuch, S., Bachrach, S.M. & Martin, J.M.L. Conformational Equilibria in Butane-1,4-diol: A Benchmark of a Prototypical System with Strong Intramolecular H-bonds. *J. Phys. Chem. A* **118**, 293-303 (2014).
78. Yu, L.-J. & Karton, A. Assessment of theoretical procedures for a diverse set of isomerization reactions involving double-bond migration in conjugated dienes. *Chem. Phys.* **441**, 166-177 (2014).
79. Yu, L.-J., Sarrami, F., Karton, A. & O'Reilly, R.J. An assessment of theoretical procedures for π-conjugation stabilisation energies in enones. *Mol. Phys.* **113**, 1284-1296 (2015).
80. Gruzman, D., Karton, A. & Martin, J.M.L. Performance of Ab Initio and Density Functional Methods for Conformational Equilibria of C_nH2_{n+2} Alkane Isomers (n = 4−8). *J. Phys. Chem. A* **113**, 11974-11983 (2009).
81. Wilke, J.J., Lind, M.C., Schaefer, H.F., Császár, A.G. & Allen, W.D. Conformers of Gaseous Cysteine. *J. Chem. Theory Comput.* **5**, 1511-1523 (2009).
82. Martin, J.M.L. What Can We Learn about Dispersion from the Conformer Surface of n-Pentane? *J. Phys. Chem. A* **117**, 3118-3132 (2013).
83. Yoo, S., Aprà, E., Zeng, X.C. & Xantheas, S.S. High-Level Ab Initio Electronic Structure Calculations of Water Clusters $(H_2O)_{16}$ and $(H_2O)_{17}$: A New Global Minimum for $(H_2O)_{16}$. *J. Phys. Chem. Lett.* **1**, 3122-3127 (2010).
84. Curtiss, L.A., Raghavachari, K., Trucks, G.W. & Pople, J.A. Gaussian-2 theory for molecular energies of first- and second-row compounds. *J. Chem. Phys.* **94**, 7221-7230 (1991).
85. Grimme, S., Kruse, H., Goerigk, L. & Erker, G. The Mechanism of Dihydrogen Activation by Frustrated Lewis Pairs Revisited. *Angew. Chem. Int. Ed.* **49**, 1402-1405 (2010).
86. Goerigk, L. & Grimme, S. Efficient and Accurate Double-Hybrid-Meta-GGA Density Functionals—Evaluation with the Extended GMTKN30 Database for General Main Group Thermochemistry, Kinetics, and Noncovalent Interactions. *J. Chem. Theory Comput.* **7**,

291-309 (2011).
87. Krieg, H. & Grimme, S. Thermochemical benchmarking of hydrocarbon bond separation reaction energies: Jacob's ladder is not reversed! *Mol. Phys.* **108**, 2655-2666 (2010).
88. O'Reilly, R.J. & Karton, A. A dataset of highly accurate homolytic N-Br bond dissociation energies obtained by Means of W2 theory. *Int. J. Quantum Chem.* **116**, 52-60 (2016).
89. Karton, A., Schreiner, P.R. & Martin, J.M.L. Heats of formation of platonic hydrocarbon cages by means of high-level thermochemical procedures. *J. Comput. Chem.* **37**, 49-58 (2016).
90. Yu, H. & Truhlar, D.G. Components of the Bond Energy in Polar Diatomic Molecules, Radicals, and Ions Formed by Group-1 and Group-2 Metal Atoms. *J. Chem. Theory Comput.* **11**, 2968-2983 (2015).
91. Zhang, W., Truhlar, D.G. & Tang, M. Tests of Exchange-Correlation Functional Approximations Against Reliable Experimental Data for Average Bond Energies of 3d Transition Metal Compounds. *J. Chem. Theory Comput.* **9**, 3965-3977 (2013).
92. Averkiev, B.B., Zhao, Y. & Truhlar, D.G. Binding energy of d10 transition metals to alkenes by wave function theory and density functional theory. *J. Mol. Catal. A Chem.* **324**, 80-88 (2010).
93. Xu, X., Zhang, W., Tang, M. & Truhlar, D.G. Do Practical Standard Coupled Cluster Calculations Agree Better than Kohn–Sham Calculations with Currently Available Functionals When Compared to the Best Available Experimental Data for Dissociation Energies of Bonds to 3d Transition Metals? *J. Chem. Theory Comput.* **11**, 2036-2052 (2015).
94. Hoyer, C.E., Manni, G.L., Truhlar, D.G. & Gagliardi, L. Controversial electronic structures and energies of Fe_2, Fe_2^+, and Fe_2^- resolved by RASPT2 calculations. *J. Chem. Phys.* **141**, 204309 (2014).
95. Luo, S., Averkiev, B., Yang, K.R., Xu, X. & Truhlar, D.G. Density Functional Theory of Open-Shell Systems. The 3d-Series Transition-Metal Atoms and Their Cations. *J. Chem. Theory Comput.* **10**, 102-121 (2014).
96. National Institute of Standards and Technology Chemistry WebBook. http://webbook.nist.gov/cgi/cbook.cgi?ID=C7446119&Units=SI (accessed July 26, 2015).
97. National Institute of Standards and Technology Chemistry WebBook. http://webbook.nist.gov/cgi/cbook.cgi?ID=C63344865&Units=SI (accessed July 26, 2015).
98. National Institute of Standards and Technology Chemistry WebBook. http://webbook.nist.gov/cgi/cbook.cgi?ID=C7664939&Units=SI&Mask=1#Thermo-Gas (accessed July 26, 2015).
99. Luo, S. & Truhlar, D.G. How Evenly Can Approximate Density Functionals Treat the Different Multiplicities and Ionization States of 4d Transition Metal Atoms? *J. Chem. Theory Comput.* **8**, 4112-4126 (2012).
100. Yang, K., Peverati, R., Truhlar, D.G. & Valero, R. Density functional study of multiplicity-changing valence and Rydberg excitations of p-block elements: Delta self-consistent field, collinear spin-flip time-dependent density functional theory (DFT), and conventional time-dependent DFT. *J. Chem. Phys.* **135**, 044118 (2011).
101. Zheng, J., Zhao, Y. & Truhlar, D.G. Representative Benchmark Suites for Barrier Heights of Diverse Reaction Types and Assessment of Electronic Structure Methods for Thermochemical Kinetics. *J. Chem. Theory Comput.* **3**, 569-582 (2007).

102. Karton, A., Tarnopolsky, A., Lamère, J.-F., Schatz, G.C. & Martin, J.M.L. Highly Accurate First-Principles Benchmark Data Sets for the Parametrization and Validation of Density Functional and Other Approximate Methods. Derivation of a Robust, Generally Applicable, Double-Hybrid Functional for Thermochemistry and Thermochemical Kinetics. *J. Phys. Chem. A* **112**, 12868-12886 (2008).

103. Karton, A., Daon, S. & Martin, J.M.L. W4-11: A high-confidence benchmark dataset for computational thermochemistry derived from first-principles W4 data. *Chem. Phys. Lett.* **510**, 165-178 (2011).

104. Hohenstein, E.G. & Sherrill, C.D. Effects of Heteroatoms on Aromatic π−π Interactions: Benzene−Pyridine and Pyridine Dimer. *J. Phys. Chem. A* **113**, 878-886 (2009).

105. Sherrill, C.D., Takatani, T. & Hohenstein, E.G. An Assessment of Theoretical Methods for Nonbonded Interactions: Comparison to Complete Basis Set Limit Coupled-Cluster Potential Energy Curves for the Benzene Dimer, the Methane Dimer, Benzene−Methane, and Benzene−H2S. *J. Phys. Chem. A* **113**, 10146-10159 (2009).

106. Takatani, T. & David Sherrill, C. Performance of spin-component-scaled Møller–Plesset theory (SCS-MP2) for potential energy curves of noncovalent interactions. *Phys. Chem. Chem. Phys.* **9**, 6106-6114 (2007).

107. Jurečka, P., Šponer, J., Černý, J. & Hobza, P. Benchmark database of accurate (MP2 and CCSD(T) complete basis set limit) interaction energies of small model complexes, DNA base pairs, and amino acid pairs. *Phys. Chem. Chem. Phys.* **8**, 1985-1993 (2006).

108. Zhao, Y. & Truhlar, D.G. Benchmark Databases for Nonbonded Interactions and Their Use To Test Density Functional Theory. *J. Chem. Theory Comput.* **1**, 415-432 (2005).

109. Leverentz, H.R., Siepmann, J.I., Truhlar, D.G., Loukonen, V. & Vehkamäki, H. Energetics of Atmospherically Implicated Clusters Made of Sulfuric Acid, Ammonia, and Dimethyl Amine. *J. Phys. Chem. A* **117**, 3819-3825 (2013).

110. Li, X., Xu, X., You, X. & Truhlar, D.G. Benchmark Calculations for Bond Dissociation Enthalpies of Unsaturated Methyl Esters and the Bond Dissociation Enthalpies of Methyl Linolenate. *J. Phys. Chem. A* **120**, 4025-4036 (2016).

111. Husch, T., Freitag, L. & Reiher, M. Calculation of Ligand Dissociation Energies in Large Transition-Metal Complexes. *J. Chem. Theory Comput.* **14**, 2456-2468 (2018).

112. Weymuth, T., Couzijn, E.P.A., Chen, P. & Reiher, M. New Benchmark Set of Transition-Metal Coordination Reactions for the Assessment of Density Functionals. *J. Chem. Theory Comput.* **10**, 3092-3103 (2014).

113. Sun, Y. & Chen, H. Performance of Density Functionals for Activation Energies of Zr-Mediated Reactions. *J. Chem. Theory Comput.* **9**, 4735-4743 (2013).

114. Sun, Y. & Chen, H. Performance of Density Functionals for Activation Energies of Re-Catalyzed Organic Reactions. *J. Chem. Theory Comput.* **10**, 579-588 (2014).

115. Hu, L. & Chen, H. Assessment of DFT Methods for Computing Activation Energies of Mo/W-Mediated Reactions. *J. Chem. Theory Comput.* **11**, 4601-4614 (2015).

116. Hoyer, C.E., Gagliardi, L. & Truhlar, D.G. Multiconfiguration Pair-Density Functional Theory Spectral Calculations Are Stable to Adding Diffuse Basis Functions. *J. Phys. Chem. Lett.* **6**, 4184-4188 (2015).

117. Send, R., Kühn, M. & Furche, F. Assessing Excited State Methods by Adiabatic Excitation Energies. *J. Chem. Theory Comput.* **7**, 2376-2386 (2011).

118. Stein, T., Kronik, L. & Baer, R. Reliable Prediction of Charge Transfer Excitations in Molecular Complexes Using Time-Dependent Density Functional Theory. *J. Am. Chem. Soc.* **131**, 2818-2820 (2009).
119. Isegawa, M., Peverati, R. & Truhlar, D.G. Performance of recent and high-performance approximate density functionals for time-dependent density functional theory calculations of valence and Rydberg electronic transition energies. *J. Chem. Phys.* **137**, 244104 (2012).
120. Zaari, R.R. & Wong, S.Y.Y. Photoexcitation of 11-Z-cis-7,8-dihydro retinal and 11-Z-cis retinal: A comparative computational study. *Chem. Phys. Lett.* **469**, 224-228 (2009).
121. Li, R., Zheng, J. & Truhlar, D.G. Density functional approximations for charge transfer excitations with intermediate spatial overlap. *Phys. Chem. Chem. Phys.* **12**, 12697-12701 (2010).
122. Ghosh, S., Sonnenberger, A.L., Hoyer, C.E., Truhlar, D.G. & Gagliardi, L. Multiconfiguration Pair-Density Functional Theory Outperforms Kohn–Sham Density Functional Theory and Multireference Perturbation Theory for Ground-State and Excited-State Charge Transfer. *J. Chem. Theory Comput.* **11**, 3643-3649 (2015).
123. Isegawa, M. & Truhlar, D.G. Valence excitation energies of alkenes, carbonyl compounds, and azabenzenes by time-dependent density functional theory: Linear response of the ground state compared to collinear and noncollinear spin-flip TDDFT with the Tamm-Dancoff approximation. *J. Chem. Phys.* **138**, 134111 (2013).
124. Schreiber, M., Silva-Junior, M.R., Sauer, S.P.A. & Thiel, W. Benchmarks for electronically excited states: CASPT2, CC2, CCSD, and CC3. *J. Chem. Phys.* **128**, 134110 (2008).
125. Hoyer, C.E., Ghosh, S., Truhlar, D.G. & Gagliardi, L. Multiconfiguration Pair-Density Functional Theory Is as Accurate as CASPT2 for Electronic Excitation. *J. Phys. Chem. Lett.* **7**, 586-591 (2016).
126. Verma, P. & Truhlar, D.G. HLE16: A Local Kohn–Sham Gradient Approximation with Good Performance for Semiconductor Band Gaps and Molecular Excitation Energies. *J. Phys. Chem. Lett.* **8**, 380-387 (2017).
127. Posada-Borbón, A. & Posada-Amarillas, A. Theoretical DFT study of homonuclear and binary transition-metal dimers. *Chem. Phys. Lett.* **618**, 66-71 (2015).
128. National Institute of Standards and Technology Computational Chemistry Comparison and Benchmark Database. https://cccbdb.nist.gov/expbondlengths1.asp, accessed on Oct. 29, 2014.
129. Piccardo, M., Penocchio, E., Puzzarini, C., Biczysko, M. & Barone, V. Semi-Experimental Equilibrium Structure Determinations by Employing B3LYP/SNSD Anharmonic Force Fields: Validation and Application to Semirigid Organic Molecules. *J. Phys. Chem. A* **119**, 2058-2082 (2015).
130. Xu, X., Alecu, I.M. & Truhlar, D.G. How Well Can Modern Density Functionals Predict Internuclear Distances at Transition States? *J. Chem. Theory Comput.* **7**, 1667-1676 (2011).
131. Marenich, A.V., Jerome, S.V., Cramer, C.J. & Truhlar, D.G. Charge Model 5: An Extension of Hirshfeld Population Analysis for the Accurate Description of Molecular Interactions in Gaseous and Condensed Phases. *J. Chem. Theory Comput.* **8**, 527-541 (2012).
132. Verma, P. & Truhlar, D.G. Can Kohn–Sham density functional theory predict accurate charge distributions for both single-reference and multi-reference molecules? *Phys. Chem. Chem. Phys.* **19**, 12898-12912 (2017).

133. Goerigk, L. et al. A look at the density functional theory zoo with the advanced GMTKN55 database for general main group thermochemistry, kinetics and noncovalent interactions. *Phys. Chem. Chem. Phys.* **19**, 32184-32215 (2017).
134. Grimme, S. Semiempirical hybrid density functional with perturbative second-order correlation. *J. Chem. Phys.* **124**, 034108 (2006).
135. Grimme, S. et al. Consistent Theoretical Description of 1,3-Dipolar Cycloaddition Reactions. *J. Phys. Chem. A* **110**, 2583-2586 (2006).
136. Piacenza, M. & Grimme, S. Systematic quantum chemical study of DNA-base tautomers. *J. Comput. Chem.* **25**, 83-99 (2004).
137. Woodcock, H.L., Schaefer, H.F. & Schreiner, P.R. Problematic Energy Differences between Cumulenes and Poly-ynes: Does This Point to a Systematic Improvement of Density Functional Theory? *J. Phys. Chem. A* **106**, 11923-11931 (2002).
138. Schreiner, P.R., Fokin, A.A., Pascal, R.A. & de Meijere, A. Many Density Functional Theory Approaches Fail To Give Reliable Large Hydrocarbon Isomer Energy Differences. *Org. Lett.* **8**, 3635-3638 (2006).
139. Lepetit, C., Chermette, H., Gicquel, M., Heully, J.-L. & Chauvin, R. Description of Carbo-oxocarbons and Assessment of Exchange-Correlation Functionals for the DFT Description of Carbo-mers. *J. Phys. Chem. A* **111**, 136-149 (2007).
140. Lee, J.S. Accurate ab Initio Binding Energies of Alkaline Earth Metal Clusters. *J. Phys. Chem. A* **109**, 11927-11932 (2005).
141. Karton, A. & Martin, J.M.L. Explicitly correlated benchmark calculations on C_8H_8 isomer energy separations: how accurate are DFT, double-hybrid, and composite ab initio procedures? *Mol. Phys.* **110**, 2477-2491 (2012).
142. Zhao, Y. et al. Thermochemical Kinetics for Multireference Systems: Addition Reactions of Ozone. *J. Phys. Chem. A* **113**, 5786-5799 (2009).
143. Zhao, Y. & Truhlar, D.G. The M06 suite of density functionals for main group thermochemistry, thermochemical kinetics, noncovalent interactions, excited states, and transition elements: two new functionals and systematic testing of four M06-class functionals and 12 other functionals. *Theor. Chem. Acc.* **120**, 215-241 (2008).
144. Manna, D. & Martin, J.M.L. What Are the Ground State Structures of C_{20} and C_{24}? An Explicitly Correlated Ab Initio Approach. *J. Phys. Chem. A* **120**, 153-160 (2016).
145. Friedrich, J. & Hänchen, J. Incremental CCSD(T)(F12*)|MP2: A Black Box Method To Obtain Highly Accurate Reaction Energies. *J. Chem. Theory Comput.* **9**, 5381-5394 (2013).
146. Friedrich, J. Efficient Calculation of Accurate Reaction Energies—Assessment of Different Models in Electronic Structure Theory. *J. Chem. Theory Comput.* **11**, 3596-3609 (2015).
147. Neese, F., Schwabe, T., Kossmann, S., Schirmer, B. & Grimme, S. Assessment of Orbital-Optimized, Spin-Component Scaled Second-Order Many-Body Perturbation Theory for Thermochemistry and Kinetics. *J. Chem. Theory Comput.* **5**, 3060-3073 (2009).
148. Johnson, E.R., Mori-Sánchez, P., Cohen, A.J. & Yang, W. Delocalization errors in density functionals and implications for main-group thermochemistry. *J. Chem. Phys.* **129**, 204112 (2008).
149. Curtiss, L.A., Raghavachari, K., Redfern, P.C. & Pople, J.A. Assessment of Gaussian-2 and density functional theories for the computation of enthalpies of formation. *J. Chem. Phys.* **106**, 1063-1079 (1997).

150. Parthiban, S. & Martin, J.M.L. Assessment of W1 and W2 theories for the computation of electron affinities, ionization potentials, heats of formation, and proton affinities. *J. Chem. Phys.* **114**, 6014-6029 (2001).

151. Zhao, Y. & Truhlar, D.G. Assessment of Density Functionals for π Systems: Energy Differences between Cumulenes and Poly-ynes; Proton Affinities, Bond Length Alternation, and Torsional Potentials of Conjugated Polyenes; and Proton Affinities of Conjugated Shiff Bases. *J. Phys. Chem. A* **110**, 10478-10486 (2006).

152. Zhao, Y., Ng, H.T., Peverati, R. & Truhlar, D.G. Benchmark Database for Ylidic Bond Dissociation Energies and Its Use for Assessments of Electronic Structure Methods. *J. Chem. Theory Comput.* **8**, 2824-2834 (2012).

153. Steinmann, S.N., Csonka, G. & Corminboeuf, C. Unified Inter- and Intramolecular Dispersion Correction Formula for Generalized Gradient Approximation Density Functional Theory. *J. Chem. Theory Comput.* **5**, 2950-2958 (2009).

154. Guner, V. et al. A Standard Set of Pericyclic Reactions of Hydrocarbons for the Benchmarking of Computational Methods: The Performance of ab Initio, Density Functional, CASSCF, CASPT2, and CBS-QB3 Methods for the Prediction of Activation Barriers, Reaction Energetics, and Transition State Geometries. *J. Phys. Chem. A* **107**, 11445-11459 (2003).

155. Ess, D.H. & Houk, K.N. Activation Energies of Pericyclic Reactions: Performance of DFT, MP2, and CBS-QB3 Methods for the Prediction of Activation Barriers and Reaction Energetics of 1,3-Dipolar Cycloadditions, and Revised Activation Enthalpies for a Standard Set of Hydrocarbon Pericyclic Reactions. *J. Phys. Chem. A* **109**, 9542-9553 (2005).

156. Dinadayalane, T.C., Vijaya, R., Smitha, A. & Sastry, G.N. Diels−Alder Reactivity of Butadiene and Cyclic Five-Membered Dienes ($(CH)_4X$, X = CH_2, SiH_2, O, NH, PH, and S) with Ethylene: A Benchmark Study. *J. Phys. Chem. A* **106**, 1627-1633 (2002).

157. Goerigk, L. & Sharma, R. The INV24 test set: how well do quantum-chemical methods describe inversion and racemization barriers? *Can. J. Chem.* **94**, 1133-1143 (2016).

158. Řezáč, J., Riley, K.E. & Hobza, P. S66: A Well-balanced Database of Benchmark Interaction Energies Relevant to Biomolecular Structures. *J. Chem. Theory Comput.* **7**, 2427-2438 (2011).

159. Setiawan, D., Kraka, E. & Cremer, D. Strength of the Pnicogen Bond in Complexes Involving Group Va Elements N, P, and As. *J. Phys. Chem. A* **119**, 1642-1656 (2015).

160. Kozuch, S. & Martin, J.M.L. Halogen Bonds: Benchmarks and Theoretical Analysis. *J. Chem. Theory Comput.* **9**, 1918-1931 (2013).

161. Řezáč, J., Riley, K.E. & Hobza, P. Benchmark Calculations of Noncovalent Interactions of Halogenated Molecules. *J. Chem. Theory Comput.* **8**, 4285-4292 (2012).

162. Grimme, S., Steinmetz, M. & Korth, M. How to Compute Isomerization Energies of Organic Molecules with Quantum Chemical Methods. *J. Org. Chem.* **72**, 2118-2126 (2007).

163. Sure, R., Hansen, A., Schwerdtfeger, P. & Grimme, S. Comprehensive theoretical study of all 1812 C60 isomers. *Phys. Chem. Chem. Phys.* **19**, 14296-14305 (2017).

164. Schwabe, T. & Grimme, S. Double-hybrid density functionals with long-range dispersion corrections: higher accuracy and extended applicability. *Phys. Chem. Chem. Phys.* **9**, 3397-3406 (2007).

165. Grimme, S. Seemingly Simple Stereoelectronic Effects in Alkane Isomers and the

Implications for Kohn–Sham Density Functional Theory. *Angew. Chem. Int. Ed.* **45**, 4460-4464 (2006).

166. Řeha, D. et al. Structure and IR Spectrum of Phenylalanyl–Glycyl–Glycine Tripetide in the Gas-Phase: IR/UV Experiments, Ab Initio Quantum Chemical Calculations, and Molecular Dynamic Simulations. *Chem. Eur. J.* **11**, 6803-6817 (2005).
167. Goerigk, L., Karton, A., Martin, J.M.L. & Radom, L. Accurate quantum chemical energies for tetrapeptide conformations: why MP2 data with an insufficient basis set should be handled with caution. *Phys. Chem. Chem. Phys.* **15**, 7028-7031 (2013).
168. Fogueri, U.R., Kozuch, S., Karton, A. & Martin, J.M.L. The Melatonin Conformer Space: Benchmark and Assessment of Wave Function and DFT Methods for a Paradigmatic Biological and Pharmacological Molecule. *J. Phys. Chem. A* **117**, 2269-2277 (2013).
169. Csonka, G.I., French, A.D., Johnson, G.P. & Stortz, C.A. Evaluation of Density Functionals and Basis Sets for Carbohydrates. *J. Chem. Theory Comput.* **5**, 679-692 (2009).
170. Kruse, H. et al. Quantum Chemical Benchmark Study on 46 RNA Backbone Families Using a Dinucleotide Unit. *J. Chem. Theory Comput.* **11**, 4972-4991 (2015).
171. Kesharwani, M.K., Karton, A. & Martin, J.M.L. Benchmark ab Initio Conformational Energies for the Proteinogenic Amino Acids through Explicitly Correlated Methods. Assessment of Density Functional Methods. *J. Chem. Theory Comput.* **12**, 444-454 (2016).
172. Řezáč, J., Huang, Y., Hobza, P. & Beran, G.J.O. Benchmark Calculations of Three-Body Intermolecular Interactions and the Performance of Low-Cost Electronic Structure Methods. *J. Chem. Theory Comput.* **11**, 3065-3079 (2015).
173. Li, S., Smith, D.G.A. & Patkowski, K. An accurate benchmark description of the interactions between carbon dioxide and polyheterocyclic aromatic compounds containing nitrogen. *Phys. Chem. Chem. Phys.* **17**, 16560-16574 (2015).
174. Faver, J.C. et al. Formal Estimation of Errors in Computed Absolute Interaction Energies of Protein−Ligand Complexes. *J. Chem. Theory Comput.* **7**, 790-797 (2011).
175. Lynch, B.J., Zhao, Y. & Truhlar, D.G. Effectiveness of Diffuse Basis Functions for Calculating Relative Energies by Density Functional Theory. *J. Phys. Chem. A* **107**, 1384-1388 (2003).
176. Chakravorty, S.J., Gwaltney, S.R., Davidson, E.R., Parpia, F.A. & p Fischer, C.F. Ground-state correlation energies for atomic ions with 3 to 18 electrons. *Phys. Rev. A* **47**, 3649-3670 (1993).
177. Chan, B., Gill, P.M.W. & Kimura, M. Assessment of DFT Methods for Transition Metals with the TMC151 Compilation of Data Sets and Comparison with Accuracies for Main-Group Chemistry. *J. Chem. Theory Comput.* **15**, 3610-3622 (2019).
178. Chan, B. The CUAGAU Set of Coupled-Cluster Reference Data for Small Copper, Silver, and Gold Compounds and Assessment of DFT Methods. *J. Phys. Chem. A* **123**, 5781-5788 (2019).
179. Mardirossian, N. & Head-Gordon, M. Thirty years of density functional theory in computational chemistry: an overview and extensive assessment of 200 density functionals. *Mol. Phys.* **115**, 2315-2372 (2017).
180. P. Verma & Truhlar, D.G. Data from "Geometries for Minnesota Database 2019", Data Repository for the University of Minnesota. https://doi.org/10.13020/217y-8g32 (2019)
181. Manna, D., Kesharwani, M.K., Sylvetsky, N. & Martin, J.M.L. Conventional and Explicitly

Correlated ab Initio Benchmark Study on Water Clusters: Revision of the BEGDB and WATER27 Data Sets. *J. Chem. Theory Comput.* **13**, 3136-3152 (2017).

182. Perdew, J.P., Burke, K. & Ernzerhof, M. Generalized Gradient Approximation Made Simple *Phys. Rev. Lett.* **77**, 3865-3868 (1996).
183. Grimme, S., Ehrlich, S. & Goerigk, L. Effect of the damping function in dispersion corrected density functional theory. *J. Comput. Chem.* **32**, 1456-1465 (2011).
184. Tao, J., Perdew, J.P., Staroverov, V.N. & Scuseria, G.E. Climbing the density functional ladder: nonempirical meta-generalized gradient approximation designed for molecules and solids. *Phys. Rev. Lett.* **91**, 146401 (2003).
185. Zhao, Y. & Truhlar, D.G. A new local density functional for main-group thermochemistry, transition metal bonding, thermochemical kinetics, and noncovalent interactions. *J. Chem. Phys.* **125**, 194101 (2006).
186. Goerigk, L. & Grimme, S. A thorough benchmark of density functional methods for general main group thermochemistry, kinetics, and noncovalent interactions. *Phys. Chem. Chem. Phys.* **13**, 6670-6688 (2011).
187. Yu, H.S., He, X. & Truhlar, D.G. MN15-L: A New Local Exchange-Correlation Functional for Kohn-Sham Density Functional Theory with Broad Accuracy for Atoms, Molecules, and Solids. *J. Chem. Theory Comput.* **12**, 1280-1293 (2016).
188. Wang, Y., Jin, X., Yu, H.S., Truhlar, D.G. & He, X. Revised M06-L functional for improved accuracy on chemical reaction barrier heights, noncovalent interactions, and solid-state physics. *Proc. Natl. Acad. Sci. U.S.A.* **114**, 8487-8492 (2017).
189. Sun, J., Ruzsinszky, A. & Perdew, J.P. Strongly Constrained and Appropriately Normed Semilocal Density Functional. *Phys. Rev. Lett.* **115**, 036402 (2015).
190. Brandenburg, J.G., Bates, J.E., Sun, J. & Perdew, J.P. Benchmark tests of a strongly constrained semilocal functional with a long-range dispersion correction. *Phys. Rev. B* **94**, 115144 (2016).
191. Chai, J.D. & Head-Gordon, M. Long-range corrected hybrid density functionals with damped atom-atom dispersion corrections. *Phys. Chem. Chem. Phys.* **10**, 6615-6620 (2008).
192. Grimme, S. Semiempirical GGA-type density functional constructed with a long-range dispersion correction. *J. Comput. Chem.* **27**, 1787-1799 (2006).
193. Peverati, R. & Truhlar, D.G. Improving the Accuracy of Hybrid Meta-GGA Density Functionals by Range Separation. *J. Phys. Chem. Lett.* **2**, 2810-2817 (2011).
194. Goerigk, L. Treating London-Dispersion Effects with the Latest Minnesota Density Functionals: Problems and Possible Solutions. *J. Phys. Chem. Lett.* **6**, 3891-3896 (2015).
195. Verma, P., Wang, Y., Ghosh, S., He, X. & Truhlar, D.G. Revised M11 Exchange-Correlation Functional for Electronic Excitation Energies and Ground-State Properties. *J. Phys. Chem. A* **123**, 2966-2990 (2019).
196. Verma, P. et al. M11plus: A Range-Separated Hybrid Meta Functional with Both Local and Rung-3.5 Correlation Terms and High Across-the-Board Accuracy for Chemical Applications. *J. Chem. Theory Comput.* **15**, 4804-4815 (2019).
197. Mardirossian, N. & Head-Gordon, M. ωB97X-V: a 10-parameter, range-separated hybrid, generalized gradient approximation density functional with nonlocal correlation, designed by a survival-of-the-fittest strategy. *Phys. Chem. Chem. Phys.* **16**, 9904-9924 (2014).
198. Mardirossian, N. & Head-Gordon, M. ωB97M-V: A combinatorially optimized, range-

separated hybrid, meta-GGA density functional with VV10 nonlocal correlation. *J. Chem. Phys.* **144**, 214110 (2016).

199. Wang, Y. et al. M06-SX screened-exchange density functional for chemistry and solid-state physics. *Proc. Natl. Acad. Sci. U.S.A.* **117**, 2294-2301 (2020).
200. Becke, A.D. Density-functional thermochemistry. III. The role of exact exchange. *J. Chem. Phys.* **98**, 5648-5652 (1993).
201. Stephens, P.J., Devlin, F.J., Chabalowski, C.F. & Frisch, M.J. Ab Initio Calculation of Vibrational Absorption and Circular Dichroism Spectra Using Density Functional Force Fields. *J. Phys. Chem.* **98**, 247-257 (1994).
202. Ernzerhof, M. & Scuseria, G.E. Assessment of the Perdew–Burke–Ernzerhof exchange-correlation functional. *J. Chem. Phys.* **110**, 5029-5036 (1999).
203. Adamo, C. & Barone, V. Toward reliable density functional methods without adjustable parameters: The PBE0 model. *J. Chem. Phys.* **110**, 6158-6170 (1999).
204. Zhao, Y., Schultz, N.E. & Truhlar, D.G. Design of Density Functionals by Combining the Method of Constraint Satisfaction with Parametrization for Thermochemistry, Thermochemical Kinetics, and Noncovalent Interactions. *J. Chem. Theory Comput.* **2**, 364-382 (2006).
205. Zhao, Y. & Truhlar, D.G. Design of Density Functionals That Are Broadly Accurate for Thermochemistry, Thermochemical Kinetics, and Nonbonded Interactions. *J. Phys. Chem. A* **109**, 5656-5667 (2005).
206. Zhao, Y. & Truhlar, D.G. Exploring the Limit of Accuracy of the Global Hybrid Meta Density Functional for Main-Group Thermochemistry, Kinetics, and Noncovalent Interactions. *J. Chem. Theory Comput.* **4**, 1849-1868 (2008).
207. Yu, H.S., He, X., Li, S.L. & Truhlar, D.G. MN15: A Kohn-Sham global-hybrid exchange-correlation density functional with broad accuracy for multi-reference and single-reference systems and noncovalent interactions. *Chem. Sci.* **7**, 5032-5051 (2016).
208. Wang, Y., Verma, P., Jin, X., Truhlar, D.G. & He, X. Revised M06 density functional for main-group and transition-metal chemistry. *Proc. Natl. Acad. Sci. U.S.A.* **115**, 10257-10262 (2018).
209. Kozuch, S., Gruzman, D. & Martin, J.M.L. DSD-BLYP: A General Purpose Double Hybrid Density Functional Including Spin Component Scaling and Dispersion Correction. *J. Phys. Chem. C* **114**, 20801-20808 (2010).
210. Schwabe, T. & Grimme, S. Towards chemical accuracy for the thermodynamics of large molecules: new hybrid density functionals including non-local correlation effects. *Phys. Chem. Chem. Phys.* **8**, 4398-4401 (2006).
211. Janesko, B.G., Verma, P., Scalmani, G., Frisch, M.J. & Truhlar, D.G. M11plus, a Range-Separated Hybrid Meta Functional Incorporating Nonlocal Rung-3.5 Correlation, Exhibits Broad Accuracy on Diverse Databases. *J. Phys. Chem. Lett.* **11**, 3045-3050 (2020).
212. Najibi, A. & Goerigk, L. The Nonlocal Kernel in van der Waals Density Functionals as an Additive Correction: An Extensive Analysis with Special Emphasis on the B97M-V and ωB97M-V Approaches. *J. Chem. Theory Comput.* **14**, 5725-5738 (2018).
213. Becke, A.D. Density-functional thermochemistry. III. The role of exact exchange. *J. Chem. Phys.* **98**, 5648-5652 (1993).
214. Becke, A.D. Density-functional exchange-energy approximation with correct asymptotic

behavior. *Phys. Rev. A* **38**, 3098-3100 (1988).

215. Lee, C., Yang, W. & Parr, R.G. Development of the Colle-Salvetti correlation-energy formula into a functional of the electron density. *Phys. Rev. B* **37**, 785-789 (1988).
216. Verma, P. & Truhlar, D.G. Status and Challenges of Density Functional Theory. *Trends Chem.* **2**, 302-318 (2020).
217. Moltved, K.A. & Kepp, K.P. Chemical Bond Energies of 3d Transition Metals Studied by Density Functional Theory. *J. Chem. Theory Comput.* **14**, 3479-3492 (2018).
218. Dohm, S., Hansen, A., Steinmetz, M., Grimme, S. & Checinski, M.P. Comprehensive Thermochemical Benchmark Set of Realistic Closed-Shell Metal Organic Reactions. *J. Chem. Theory Comput.* **14**, 2596-2608 (2018).
219. Kang, R., Lai, W., Yao, J., Shaik, S. & Chen, H. How Accurate Can a Local Coupled Cluster Approach Be in Computing the Activation Energies of Late-Transition-Metal-Catalyzed Reactions with Au, Pt, and Ir? *J. Chem. Theory Comput.* **8**, 3119-3127 (2012).
220. Truhlar, D.G. Dispersion Forces: Neither Fluctuating Nor Dispersing. *J. Chem. Educ.* **96**, 1671-1675 (2019).
221. Caricato, M., Trucks, G.W., Frisch, M.J. & Wiberg, K.B. Electronic Transition Energies: A Study of the Performance of a Large Range of Single Reference Density Functional and Wave Function Methods on Valence and Rydberg States Compared to Experiment. *J. Chem. Theory Comput.* **6**, 370-383 (2010).
222. Zhao, Y. & Truhlar, D.G. Density functional for spectroscopy: no long-range self-interaction error, good performance for Rydberg and charge-transfer states, and better performance on average than B3LYP for ground states. *J. Phys. Chem. A* **110**, 13126-13130 (2006).
223. Schultz, N.E., Zhao, Y. & Truhlar, D.G. Density Functionals for Inorganometallic and Organometallic Chemistry. *J. Phys. Chem. A* **109**, 11127-11143 (2005).
224. Mardirossian, N. & Head-Gordon, M. Characterizing and Understanding the Remarkably Slow Basis Set Convergence of Several Minnesota Density Functionals for Intermolecular Interaction Energies. *J. Chem. Theory Comput.* **9**, 4453-4461 (2013).
225. Wu, D. & Truhlar, D.G. How Accurate Are Approximate Density Functionals for Noncovalent Interaction of Very Large Molecular Systems? *J. Chem. Theory Comput.* **17**, 3967-3973 (2021).
226. Ni, Z., Guo, Y., Neese, F., Li, W. & Li, S. Cluster-in-Molecule Local Correlation Method with an Accurate Distant Pair Correction for Large Systems. *J. Chem. Theory Comput.* **17**, 756-766 (2021).
227. Maurer, L.R., Bursch, M., Grimme, S. & Hansen, A. Assessing Density Functional Theory for Chemically Relevant Open-Shell Transition Metal Reactions. *J. Chem. Theory Comput.* **17**, 6134-6151 (2021).
228. Chan, B. Assessment and development of DFT with the expanded CUAGAU-2 set of group-11 cluster systems. *Int. J. Quantum Chem.* **121**, e26453 (2021).
